# HIV risk behaviour, viraemia, and transmission across HIV cascade stages including low-level viremia: Analysis of 14 cross-sectional population-based HIV Impact Assessment surveys in sub-Saharan Africa

**DOI:** 10.1371/journal.pgph.0003030

**Published:** 2024-04-04

**Authors:** Olanrewaju Edun, Lucy Okell, Helen Chun, Anne-Cecile Z. Bissek, Clement B. Ndongmo, Judith D. Shang, Hermann Brou, Eboi Ehui, Alexandre K. Ekra, Harriet Nuwagaba-Biribonwoha, Sindisiwe S. Dlamini, Choice Ginindza, Frehywot Eshetu, Yimam G. Misganie, Sileshi Lulseged Desta, Thomas N. O. Achia, Appolonia Aoko, Sasi Jonnalagadda, Rose Wafula, Fred M. Asiimwe, Shirley Lecher, Kondwani Nkanaunena, Mtemwa K. Nyangulu, Rose Nyirenda, Anita Beukes, Johannes O. Klemens, Negussie Taffa, Andrew A. Abutu, Matthias Alagi, Man E. Charurat, Ibrahim Dalhatu, Gambo Aliyu, Collins Kamanzi, Celestine Nyagatare, Gallican N. Rwibasira, Mohamed F. Jalloh, Werner M. Maokola, George S. Mgomella, Wilford L. Kirungi, Christina Mwangi, Jennifer A. Nel, Peter A. Minchella, Gloria Gonese, Melodie A. Nasr, Stephane Bodika, Elisabeth Mungai, Hetal K. Patel, Katrina Sleeman, Kyle Milligan, Emilio Dirlikov, Andrew C. Voetsch, Ray W. Shiraishi, Jeffrey W. Imai-Eaton

**Affiliations:** 1 MRC Centre for Global Infectious Disease Analysis, School of Public Health, Imperial College London, London, United Kingdom; 2 U.S. Centers for Disease Control and Prevention - Division of Global HIV/TB, Center for Global Health, Atlanta, Georgia, United States of America; 3 Division of Health Operations Research, Ministry of Public Health, Yaoundé, Cameroon; 4 Faculty of Medicine and Biomedical Sciences, University of Yaoundé, Yaoundé, Cameroon; 5 U.S. Centers for Disease Control and Prevention - Division of Global HIV/TB, Center for Global Health, Yaoundé, Cameroon; 6 ICAP, Columbia University, Abidjan, Côte d’Ivoire; 7 National AIDS Control Programme, Ministry of Health, Public Hygiene and Universal Health Coverage, Abidjan, Côte d’Ivoire; 8 U.S. Centers for Disease Control and Prevention - Division of Global HIV/TB, Center for Global Health, Abidjan, Côte d’Ivoire; 9 ICAP at Columbia University and Department of Epidemiology, Mailman School of Public Health, Mbabane, Eswatini; 10 Ministry of Health, Mbabane, Eswatini; 11 Central Statistical Office, Mbabane, Eswatini; 12 U.S. Centers for Disease Control and Prevention Division of Global HIV/TB, Center for Global Health, Addis Ababa, Ethiopia; 13 Ethiopian Public Health Institute, HIV/AIDS and TB Research Directorate, Addis Ababa, Ethiopia; 14 School of Medicine, Zhejiang University, Hangzhou, China; 15 ICAP in Ethiopia, Mailman School of Public Health, Columbia University, Addis Ababa, Ethiopia; 16 U.S. Centers for Disease Control and Prevention - Division of Global HIV/TB, Center for Global Health, Nairobi, Kenya; 17 National AIDS and STI Control Programme, Ministry of Health, Nairobi, Kenya; 18 U.S. Centers for Disease Control and Prevention - Division of Global HIV/TB, Center for Global Health, Maseru, Lesotho; 19 U.S. Centers for Disease Control and Prevention - Division of Global HIV/TB, Center for Global Health, Lilongwe, Malawi; 20 Department of HIV/AIDS, Ministry of Health, Lilongwe, Malawi; 21 U.S. Centers for Disease Control and Prevention - Division of Global HIV/TB, Center for Global Health, Windhoek, Namibia; 22 Namibia Institute of Pathology Limited, Windhoek, Namibia; 23 Ministry of Health and Social Services, Windhoek, Namibia; 24 U.S. Centers for Disease Control and Prevention - Division of Global HIV/TB, Center for Global Health, Abuja, Nigeria; 25 Center for International Health, Education, and Biosecurity (Ciheb), University of Maryland School of Medicine, Baltimore, Maryland, United States of America; 26 Institute of Human Virology, University of Maryland School of Medicine, Baltimore, Maryland, United States of America; 27 National Agency for the Control of AIDS, Abuja, Nigeria; 28 ICAP, Columbia University, Kigali, Rwanda; 29 U.S. Centers for Disease Control and Prevention - Division of Global HIV/TB, Center for Global Health, Kigali, Rwanda; 30 HIV, STIs, Viral Hepatitis & OVDC Department, Rwanda Biomedical Center, Kigali, Rwanda; 31 U.S. Centers for Disease Control and Prevention - Division of Global HIV/TB, Center for Global Health, Dar es Salaam, Tanzania; 32 National AIDS Control Programme, Tanzania Ministry of Health, Dar es Salaam, Tanzania; 33 Ministry of Health, AIDS Control Programme, Kampala, Uganda; 34 U.S. Centers for Disease Control and Prevention - Division of Global HIV/TB, Center for Global Health, Kampala, Uganda; 35 U.S. Centers for Disease Control and Prevention Division of Global HIV/TB, Center for Global Health, Lusaka, Zambia; 36 Zimbabwe Technical Assistance, Training and Education Center for Health (Zim-TTECH), Harare, Zimbabwe; 37 U.S. Centers for Disease Control and Prevention - Division of Global HIV/TB, Center for Global Health, Harare, Zimbabwe; 38 Public Health Institute, Global Health Fellowship Program, United States of America; 39 eTeam, Somerset, New Jersey, United States of America; 40 Peraton, Herndon, Virginia, United States of America; 41 Department of Epidemiology, Center for Communicable Disease Dynamics, Harvard T.H. Chan School of Public Health, Boston, Massachusetts, United States of America; AORN Ospedali dei Colli, ITALY

## Abstract

As antiretroviral treatment (ART) coverage for people living with HIV (PLHIV) increases, HIV programmes require up-to-date information about evolving HIV risk behaviour and transmission risk, including those with low-level viremia (LLV; >50 to ≤1000 copies/mL), to guide prevention priorities. We aimed to assess differences in sexual risk behaviours, distribution of viral load (VL) and proportion of transmission across PLHIV subgroups. We analysed data from Population-based HIV Impact Assessment surveys in 14 sub-Saharan African countries during 2015–2019. We estimated adjusted prevalence ratios (aPR) of self-reported HIV high-risk behaviour (multiple partners and condomless sex) across cascade stages via generalised estimation equations. We modelled the proportions of transmission from each subgroup using relative self-reported sexual risk, a Hill function for transmission rate by VL, and proportions within cascade stages from surveys and UNAIDS country estimates for 2010–2020. Compared to PLHIV with undetectable VL (≤50 copies/mL), undiagnosed PLHIV (aPR women: 1.28 [95% CI: 1.08–1.52]; men: 1.61 [1.33–1.95]) and men diagnosed but untreated (2.06 [1.52–2.78]) were more likely to self-report high-risk sex. High-risk behaviour was not significantly associated with LLV. Mean VL was similar among undiagnosed, diagnosed but untreated, and on ART but non-suppressed sub-groups. Across surveys, undiagnosed and diagnosed but untreated contributed most to transmission (40–91% and 1–41%, respectively), with less than 1% from those with LLV. Between 2010 and 2020, the proportion of transmission from individuals on ART but non-suppressed increased. In settings with high ART coverage, effective HIV testing, ART linkage, and retention remain priorities to reduce HIV transmission. Persons with LLV are an increasing share of PLHIV but their contribution to HIV transmission was small. Improving suppression among PLHIV on ART with VL ≥1000 copies/mL will become increasingly important.

## Introduction

Over the past decade, countries most-affected by HIV in sub-Saharan Africa (SSA) have aggressively expanded HIV testing and antiretroviral treatment (ART) as part of combination HIV prevention. These efforts have increased ART coverage among adults with HIV from 25% in 2010 to 80% in 2021, and contributed to a 56% reduction in AIDS deaths and 42% decline in new HIV infections in the region [1]. Despite this progress, an estimated 730,000 adults acquired HIV in 2021, representing 56% of the 1.3 million global new adult HIV infections [1].

Understanding the relative contributions of people living with HIV (PLHIV) subgroups to ongoing HIV transmission informs prevention interventions. ‘Test-and-treat’ strategies have focused on reaching PLHIV who are undiagnosed and untreated to reduce HIV transmission [2]. However, as ART coverage reaches high levels, PLHIV on ART with detectable viremia, including low-level viremia (LLV), defined as viral load (VL) >50 but ≤1,000 copies/mL [3], represent an increasing share of PLHIV. Low-level viremia among PLHIV on treatment can be due to poor ART adherence, poor drug absorption, or drug resistance [4]. Compared to those with undetectable VL (≤50 copies/mL), PLHIV with LLV have increased risk of virological failure, acquiring drug resistance mutations, and onward HIV transmission [5–7].

Sexual HIV transmission risk correlates directly to the HIV VL in the HIV infected partner [8–10], with those with undetectable VL being non-infectious [11]. While observational data quantifying HIV infectiousness at low viraemia levels are limited, some have raised concern that PLHIV with LLV could become an increasingly important population with HIV transmission risk as this population grows and the undiagnosed and untreated population shrinks [5, 12]. This could particularly be the case if PLHIV with LLV engage in HIV risk behaviour due to a perception of being non-infectious. Studies have consistently found lower reported sexual risk among those aware of their HIV-positive status [13–16] and on ART [17, 18] compared to undiagnosed or untreated PLHIV. However, data on the association between HIV risk behaviour and virologic non-suppression (>1,000 copies/mL) or LLV are sparse, with disparate findings and limited data from SSA settings [19–23].

Since the 2000s, national household health surveys with HIV serological testing have provided evidence about the relationship between HIV status, HIV testing, and sexual risk [24]. The Population-based HIV Impact Assessment (PHIA) surveys, conducted in high HIV burden SSA countries since 2015, additionally measured VL and ART usage among PLHIV [25]. These surveys provide unique data about the determinants of HIV transmission—sexual risk behaviours and distribution of VL—across stages of the HIV testing and treatment cascade following implementation of universal ART eligibility. To guide understanding of potential HIV transmission risk following rapid ART scale-up and guide future HIV programme priorities, we (i) assessed the association between HIV sexual risk behaviour and subgroups of PLHIV defined by HIV awareness, treatment status, and virologic status; (ii) characterised the distribution of viremia and consequent risk of transmission among subgroups; and (iii) quantified the expected proportion of HIV transmission across subgroups over time.

## Methods

### Study design and participants

We analysed data from 14 PHIA surveys conducted during 2015–2019 in West/Central Africa: Cameroon (June 2017–February 2018), Côte d’Ivoire (August 2017–March 2018), Nigeria (July–December 2018); Eastern Africa: Ethiopia (October 2017–April 2018), Kenya (May 2018–March 2019), Malawi (November 2015–August 2016), Rwanda (October 2018–March 2019), Uganda (August 2016–March 2017), Tanzania (October 2016–June 2017), Zambia (March–August 2016); and Southern Africa: Eswatini (August 2016–March 2017), Lesotho (November 2016–May 2017), Namibia (June–December 2017), Zimbabwe (October 2015–August 2016) [26, 27]. For the purpose of this study, the PHIA survey data were accessed in November 2021, except the Kenya PHIA data which were accessed in September 2022. PHIAs were cross-sectional household surveys that collected standardised data across countries and assessed HIV-related health indicators. Details of survey design, sampling, and procedures are described elsewhere [28, 29]. Briefly, surveys used a stratified multistage sampling design, with households randomly selected within sampled enumeration areas. Adult household members were eligible for the survey if they slept in the household the night before the interview and were aged 15–64 years, except in Zambia and Lesotho (15–59 years), and in Tanzania, Eswatini, and Zimbabwe (≥15 years). Consenting participants completed a structured individual questionnaire, including sociodemographic information, marital and pregnancy status, and HIV testing, care, and treatment history. Participants’ also self-reported their last three sexual relationships in the past 12-months. Following interviews, HIV status was determined through home-based serological testing using country-specific HIV diagnostic algorithms with immediate return of results. HIV-positive blood samples underwent laboratory-based confirmatory testing using Geenius HIV-1/2 Confirmatory Assay (Bio-Rad, Hercules, CA), HIV RNA VL testing, and qualitative screening for selected antiretrovirals (ARVs) to determine ART usage. PHIA biomarker testing methodology has previously been described [28, 29].

All surveys received ethical approval from institutional review boards in the respective countries, the U.S. Centers for Disease Control and Prevention (CDC), and Columbia University or the University of Maryland at Baltimore (Nigeria survey). Written informed consent was obtained from adult participants; assent and parental permission was obtained for participants under the age of consent (typically 15–17 years). Secondary analysis reported here received ethical approval from the Imperial College Research Governance and Integrity Team (20IC6451).

### Study measures

Table 1 describes the sexual risk outcomes analysed. The primary risk outcome, “HIV high-risk behaviour”, was defined as self-reporting ≥2 sexual partners and condomless sex with a non-spousal partner in the past 12 months. Alternative measures were condomless sex with a casual partner, transactional partnership, condomless last sex (with any partner), and self-reporting ≥2 sexual partners with condomless last sex (with any partner).

**Table 1:** Definitions of sexual risk and exposure measures of this study.

**Primary sexual risk measure**	
**HIV high-risk behaviour**	Self-reporting both ≥2 sexual partners and condomless sex with a non-spousal partner (i.e., ex-spouse, friend, sex-worker, sex-worker client, stranger, or other) in the past 12 months
**Alternative sexual risk measures**	
**Condomless casual partnership**	Self-reporting condomless sex with a non-marital and non-cohabiting partner (i.e., friend, sex worker, sex-worker client, stranger, or other) in the past 12 months
**Transactional partnership**	Self-reporting engaging in a sexual relationship because a partner provided or was expected to provide material or other support (e.g., money, food, or shelter) in the past 12 months
**Condomless last sex (with any partner)**	Self-reporting condomless sex at last sex in the past 12 months
**Multiple sexual partnership and condomless last sex (with any partner)**	Self-reporting both ≥2 sexual partners and condomless sex in the past 12 months
**Exposure measure (defined population subgroups)**	
**HIV negative**	HIV serostatus negative
**Undiagnosed**	HIV seropositive and reported not being aware of their HIV-positive status and had no ARVs detected on measurement
**Diagnosed but untreated**	HIV seropositive and reported knowing their infection status but reported not currently taking ART and had no ARVs detected on measurement
**On ART and non-suppressed**	HIV seropositive and had ARVs detected on measurement or self-reported being on ART and had viral load >1,000 copies/mL
**On ART with low-level viremia**	HIV seropositive and had ARVs detected on measurement or self-reported being on ART and had viral load >50 to ≤1000 copies/mL
**On ART and undetectable**	HIV seropositive and had ARVs detected on measurement or self-reported being on ART and had viral load ≤50 copies/mL

The main exposure of interest was the population subgroup defined by HIV infection, diagnosis, and viremia category among those on treatment (Table 1). Participants were excluded if their VL was censored at a lower limit of quantification (LLOQ) within the LLV category (e.g., <LLOQ: 839 copies/mL) or had undetectable VL (≤50 copies/mL) but ARVs were not detected and they self-reported not taking ART (S2 Fig).

Covariates for regression analysis were age, education level, wealth quintile, marital status, pregnancy status, and urban/rural residence. For the Ethiopia survey, which was restricted to an urban-only sampling frame, the urban area size (small: <50,000; large: ≥50,000 people) was included. All other surveys were nationally representative.

### Data analyses

Analysis was restricted to sexually active adults with results for HIV status, ART usage, and VL, and who reported sexual activity. All analyses were sex-stratified. We summarised participant characteristics for each survey using medians, interquartile ranges (IQR), and survey weighted proportions using the PHIA blood test weights (PHIA weights). We calculated weighted proportions with HIV risk behaviour and proportions of PLHIV in the defined cascade subgroups for each survey, with associated design-based 95% confidence intervals. We also summarised characteristics of the pooled sample (unweighted) from all 14 surveys.

Second, to identify the association between HIV high-risk behaviour and HIV subgroup, we pooled data from the 14 surveys and used generalized estimating equations (GEE) with log-binomial distribution and exchangeable correlation structure. The GEE model incorporated survey cluster random effects and country-level fixed effects. Regression models were sex-stratified and adjusted for age, wealth quintile, education level, urban/rural residence, marital status, and pregnancy status (for women), identified from our conceptual framework (S1 Fig). To allow a non-linear relationship between age and HIV risk behaviour, age was modelled using a cubic spline with knots at ages 30 and 50 (S9 Fig). Survey weights were not used in the regression model because the model adjusted for factors determining weights. In sensitivity analysis, we repeated the regression incorporating the normalized survey weights.

Third, to characterise the distribution of viremia among PLHIV subgroups, we calculated the mean log_10_ VL among the PLHIV subgroups and plotted kernel density estimates of the log_10_-transformed VL distribution for each survey. We used a Hill function parameterised by Fraser *et al*. [30] to model the annual HIV transmission rate in a discordant partnership as a function of VL (S11 Fig). The Hill function was applied to survey-weighted individual-level observations in each PLHIV subgroup to estimate average transmission rates by sex, country, and cascade subgroup.

Finally, we combined the determinants of HIV force of infection—the relative rate of partner change (using the relative self-reported sexual risk as a proxy), per-partnership risk of transmission (transmission probability from the Hill function), and size of each PLHIV subgroup—to estimate the proportion of transmission from each subgroup using the risk equation:

$$Proportion\;of\;transmission=\frac{{pd}_{i}\;\times \;{PR}_{i}\;\times \;\beta (V_{i})}{\sum_{i=1}^{n}{\;pd}_{i}\times \;{PR}_{i}\times \;\beta (V_{i})}$$

where *pd*_*i*_ represents the proportion of PLHIV in the *i* th exposure category, *PR*_*i*_ is the prevalence ratio of HIV high-risk behaviour for the *i* th exposure category relative to the unexposed category, and *β(V_i_)* is the average transmission rate as a function of VL. Given the differential distribution of covariates adjusted for in the pooled model across the survey countries, we obtained country-specific prevalence ratios(*PR*_*i*_) by using the pooled model to predict the probability of self-reporting HIV high-risk sex for each PLHIV subgroup, accounting for the survey-weighted distribution of covariates in each group.

To explore how proportions changed over time as HIV awareness, ART coverage, and viral suppression increased, we obtained UNAIDS estimates of the proportion of PLHIV in each subgroup from 2010 to 2020 [1]. Assuming the proportion of LLV among PLHIV with VL <1,000 copies/mL and ‘HIV high-risk behaviour’ prevalence ratios were constant over time at values estimated from the surveys, we calculated the proportion of transmission attributable to each PLHIV subgroup from 2010 to 2020. Statistical significance level assessed as p <0.05. Analyses were conducted in R version 4.1.2.

Sensitivity analyses conducted includes repeating the analyses excluding the Nigeria survey, which contributed 38% of all participants, to explore the influence of the survey. We also stratified the analyses by West/Central, Eastern, and Southern Africa regions. We repeated analyses using alternative outcome measures of relative HIV transmission-risk behaviour (Table 1), and hypothetically assumed: (i) the same HIV high-risk behaviour across all groups (i.e., aPR = 1) and (ii) higher HIV high-risk behaviour among individuals on ART relative those not on ART (i.e., aPR = 1 among those on ART and aPR = 0.5 among untreated).

Lastly, to explore sensitivity to the Hill function for the relationship between viremia and infectiousness, we repeated analyses using a linear function estimated by Wilson *et al*. [31]. This function, which has been shown to overestimate the risk of transmission among people on ART [32], is likely an upper-bound risk of transmission among PLHIV on ART with LLV.

### Role of the funding source

Funders had no role in the study design, data analysis, decision to publish or preparation of the manuscript.

## Results

Among 378,166 participants (≥15 years) in the 14 surveys who reported ever having had sex, our analyses included 368,373 (97%) participants with complete data (214,305 [58%] women; 154,068 [42%] men (Table 2 and S1 Table); see S2 Fig for details of participants excluded).

**Table 2:** Summary of participant characteristics (unweighted) across all 14 surveys.

Characteristics	Pooled surveys (N = 368,373)	
	Women (n = 214,305)	Men (n = 154,068)
**Survey by region, n (%)**		
*West/Central Africa*		
Cameroon 2017–2018 (15–64y)*	12,215 (5.7)	9,641 (6.3)
Côte d’Ivoire 2017–2018 (15–64y)	8,083 (3.8)	7,610 (4.9)
Nigeria 2018 (15–64y)	81,992 (38.3)	57,860 (37.6)
*Eastern Africa*		
Ethiopia 2017–2018 (15–64y)	7,860 (3.7)	4,945 (3.2)
Kenya 2018–2019 (15–64y)	13,189 (6.2)	9,017 (5.9)
Malawi 2015–2016 (15–64y)	8,755 (4.1)	6,089 (4.0)
Rwanda 2018–2019 (15–64y)	12,938 (6.0)	10,356 (6.7)
Tanzania 2016–2017 (15–80y)	15,554 (7.3)	11,632 (7.5)
Uganda 2016–2017 (15–64y)	14,623 (6.8)	10,459 (6.8)
Zambia 2016 (15–59y)	9,110 (4.3)	6,438 (4.2)
*Southern Africa*		
Eswatini 2016–2017 (15–80y)	5,385 (2.5)	3,306 (2.1)
Lesotho 2016–2017 (15–59y)	5,962 (2.8)	3,985 (2.6)
Namibia 2017 (15–64y)	7,560 (3.5)	5,561 (3.6)
Zimbabwe 2015–2016 (15–80y)	11,079 (5.2)	7,169 (4.7)
**HIV, ART, and viremia status, n (%)**		
HIV negative	196,958 (91.9)	146,361 (95.0)
On ART undetectable	9,811 (4.6)	3,622 (2.4)
On ART low-level viremia	1,365 (0.6)	754 (0.5)
On ART non-suppressed	1,423 (0.7)	645 (0.4)
Diagnosed but untreated	998 (0.5)	469 (0.3)
Undiagnosed	3,750 (1.8)	2,217 (1.4)
**HIV high-risk behaviour, n (%)**		
No	209,214 (97.6)	140,790 (91.4)
Yes	5,091 (2.4)	13,278 (8.6)
**Condomless casual partnerships, n (%)**		
No	203,852 (95.1)	137,300 (89.1)
Yes	10,453 (4.9)	16,768 (10.9)
**Transactional partnerships, n (%)**		
No	194,800 (90.9)	147,257 (95.5)
Yes	18,528 (8.6)	5,763 (3.8)
Missing	977 (0.5)	1,048 (0.7)
**Condomless last sex**		
No	67,952 (31.7)	51,934 (33.1)
Yes	144,766 (67.6)	97,627 (63.4)
Missing	1,587 (0.7)	4,507 (2.9)
**Multiple sexual partnership and condomless last sex**		
No	207,165 (96.7)	125,999 (81.8)
Yes	5,553 (2.6)	23,562 (15.3)
Missing	1,587 (0.7)	4,507 (2.9)
**Urban/large urban dwelling, n (%)**	81,276 (38.0)	56,820 (36.9)
**Wealth quintile, n (%)**		
Lowest	44,631 (20.8)	30,582 (19.8)
Second	42,744 (19.9)	30,606 (19.9)
Middle	44,268 (20.7)	32,042 (20.8)
Fourth	41,985 (19.6)	30,633 (19.9)
Highest	40,603 (19.0)	30,147 (19.6)
Missing	72 (<0.1)	58 (<0.1)
**Level of education, n (%)**		
None	50,534 (23.6)	21847 (14.2)
Primary	79,447 (37.1)	54,413 (35.3)
Secondary	62,986 (29.4)	52,448 (34.0)
More than secondary	20,121 (9.4)	23,981 (15.6)
Missing	1,217 (0.6)	1,379 (0.9)
**Marital status, n (%)**		
Currently married	144,650 (67.5)	102,354 (66.4)
Never married	33,937 (15.8)	42,448 (27.6)
Divorced/separated	16,895 (7.9)	7,188 (4.7)
Widower/widow	18,480 (8.6)	1,862 (1.2)
Missing	343 (0.2)	216 (0.1)
**Pregnancy status, n (%)**		
Pregnant	16,794 (7.8)	-
Not pregnant	194,883 (90.9)	-
Missing	2,628 (1.3)	-

In all surveys, a higher proportion of men reported HIV high-risk behaviour (2.1–17.0% across surveys), condomless casual partnerships (1.1–21.6%), and multiple partnerships with condomless last sex (3.0–22.1%) than women (0.8–5.7%, 0.6–11.1%, and 1.1–5.3%, respectively). More women reported transactional partnerships (2.9–16.0%) than men (1.7–5.7%), with similar proportions reporting condomless last sex with any partner (S3 Fig).

In Western/Central Africa, undiagnosed individuals were the largest group of PLHIV. In Eastern and Southern African surveys, individuals on ART with undetectable VL were the largest proportion. The proportion of PLHIV with LLV varied across surveys from 5.0% (Malawi, 2015–2016) to 12.1% (Uganda, 2016–2017) among women, and 4.6% (Malawi, 2015–2016) to 18.8% (Ethiopia, 2017–2018) among men.

### Association between HIV high-risk behaviour and HIV infection, diagnosis, and treatment population subgroups

**Fig 1:**
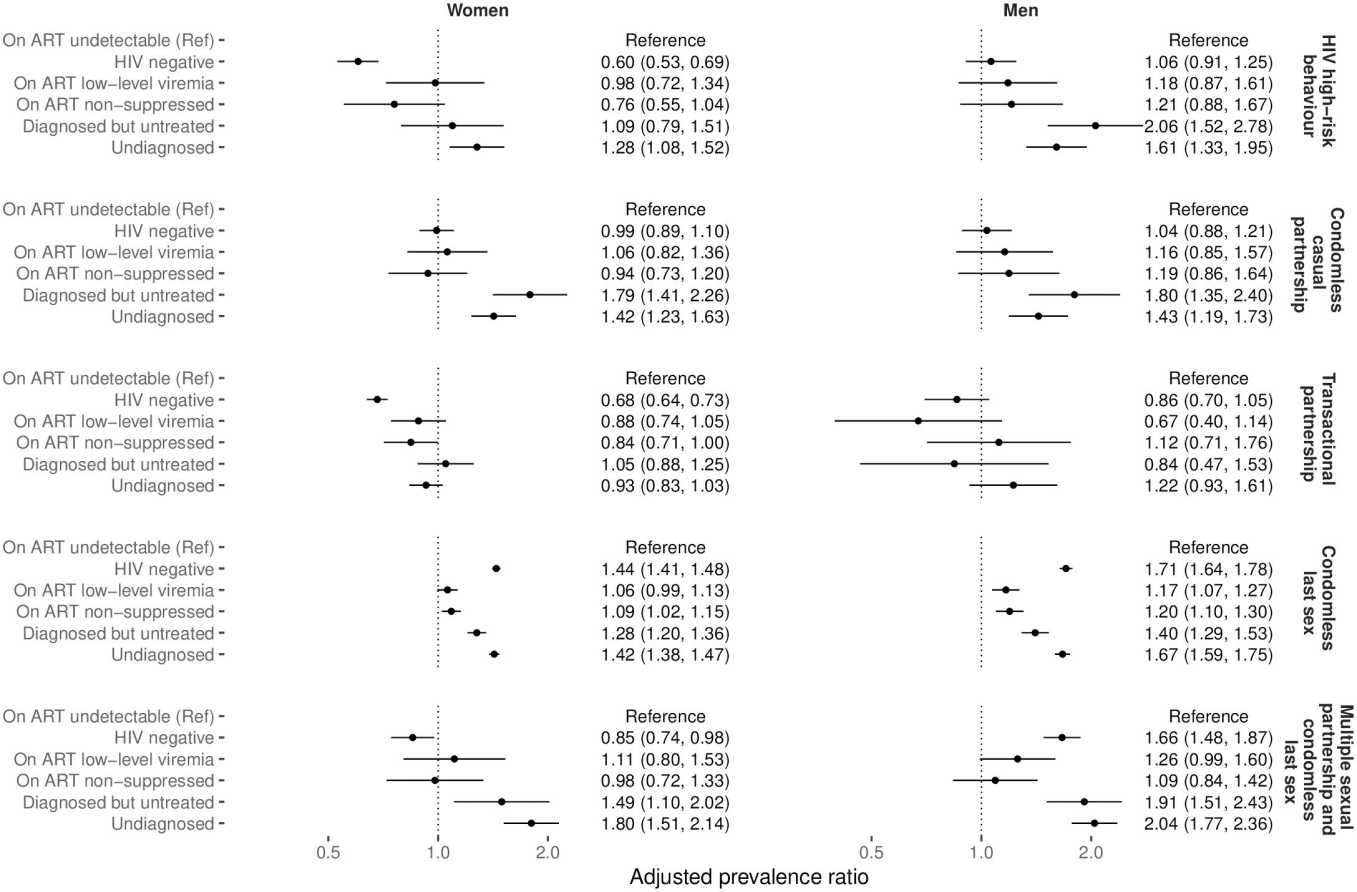
Adjusted prevalence ratio of self-reported HIV high-risk behaviour, condomless casual partnerships, transactional partnerships, condomless last sex and multiple sexual partnerships with condomless last sex for our defined subgroups. Results are stratified by sex and models were adjusted for age, education level, wealth quintile, marital status, urban/rural dwelling or urbanicity size and pregnancy status in women. Note: x-axis is log_10_ scaled. Full regression results are reported in S2–S6 Tables. Country-specific predicted prevalence ratios are reported in S4–S8 Figs, S7–S11 Tables.

Compared to women on ART with undetectable VL, undiagnosed women were more likely to report HIV high-risk behaviour (adjusted prevalence ratio [aPR]: 1.28, 95% confidence interval [95% CI]: 1.08–1.52), condomless casual partnership (aPR: 1.42; 1.23–1.63), condomless last sex (aPR: 1.42, 1.38–1.47) and multiple sexual partnership with condomless last sex (aPR: 1.80, 1.51–2.14). Women who were diagnosed but untreated were more likely to report condomless casual partnership (aPR: 1.79; 1.41–2.26), condomless last sex (aPR: 1.28, 1.20–1.36) and multiple sexual partnerships with condomless last sex (aPR: 1.49, 1.11–2.02; Fig 1; S2–S6 Tables).

Similarly, compared to men with undetectable VL, undiagnosed men were more likely to report HIV high-risk behaviour (aPR: 1.61; 1.33–1.95), condomless casual partnership (aPR: 1.43; 1.19–1.73), condomless last sex (aPR: 1.67; 1.59–1.75) and multiple sexual partnership with condomless last sex (aPR: 2.04, 1.77–2.36). Men who were diagnosed but untreated were more likely to report HIV high-risk behaviour (aPR: 2.06; 1.52–2.78), condomless casual partnership (aPR: 1.80; 1.35–2.40), condomless last sex (aPR: 1.40; 1.29–1.53) and multiple sexual partnerships with condomless last sex (aPR: 1.91; 1.51–2.43).

Among both men and women, those with LLV did not report significantly different HIV high-risk behaviour, condomless casual partnerships, transactional partnerships, or multiple sexual partnerships with condomless last sex compared to those with undetectable VL. HIV negative women were less likely to report HIV high-risk behaviour (aPR: 0.60; 0.53–0.69), transactional partnerships (aPR: 0.68; 0.64–0.73), and multiple sexual partnerships with condomless last sex. Condomless last sex was more common among all untreated PLHIV subgroups and among HIV negative men and women than those with undetectable VL. Regression results were similar in the weighted and unweighted analysis (S14 Fig).

### Distribution of viremia and transmission rates across PLHIV subgroups

**Fig 2:**
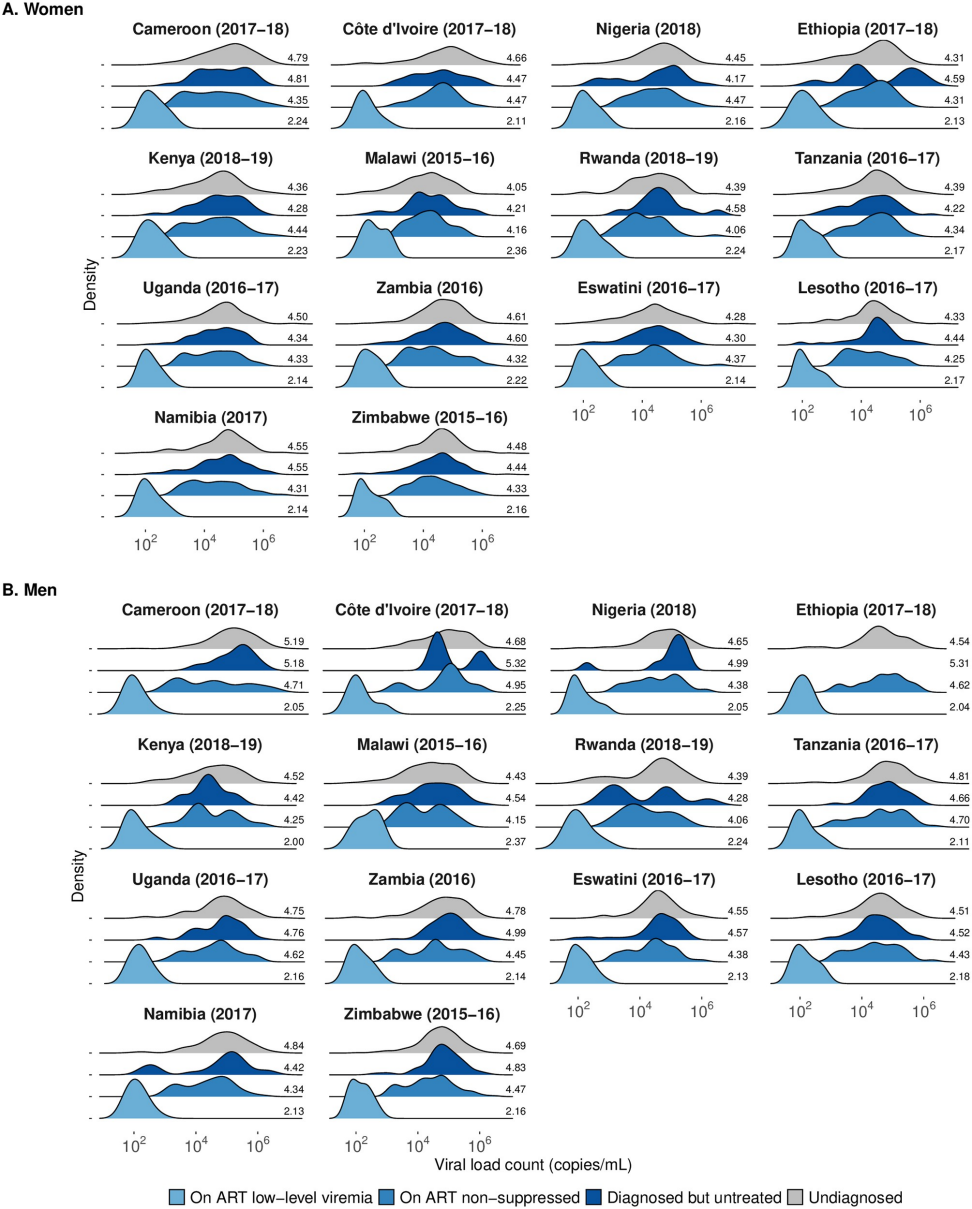
Distribution of plasma HIV RNA and mean log_10_ VL among (a) women and (b) men with LLV, non-suppressed VL and those diagnosed but untreated and undiagnosed across 14 PHIA surveys. Note: unable to reliably estimate distribution due to small number of men diagnosed but untreated in the Ethiopia PHIA survey.

Mean log_10_ VL was similar among those undiagnosed, untreated, and on treatment with non-suppressed VL (Fig 2), ranging from 4.05 log_10_ copies/mL (Malawi 2015–2016, undiagnosed women) to 5.32 log_10_ copies/mL (Côte d’Ivoire 2017–2018, diagnosed but untreated men). Among PLHIV on treatment with LLV, mean log_10_ VL ranged from 2.00 (IQR: 1.78, 2.03; Kenya 2018–2019, men) to 2.37 log_10_ copies/mL (IQR: 2.04, 2.64; Malawi 2015–2016, men).

Across all countries, the predicted mean transmission rate using the Hill function was lowest among PLHIV on treatment with LLV, ranging between 0.003 to 0.007 per year. Transmission rates were similar across the other categories (undiagnosed: 0.15 to 0.24 per year; diagnosed but untreated: 0.16 to 0.24 per year; those on treatment with viral non-suppression: 0.14 to 0.26 per year) reflecting the similarity of the VL distributions (S10 and S11 Figs, S12 Table).

### Contribution of PLHIV subgroups to HIV transmission

**Fig 3:**
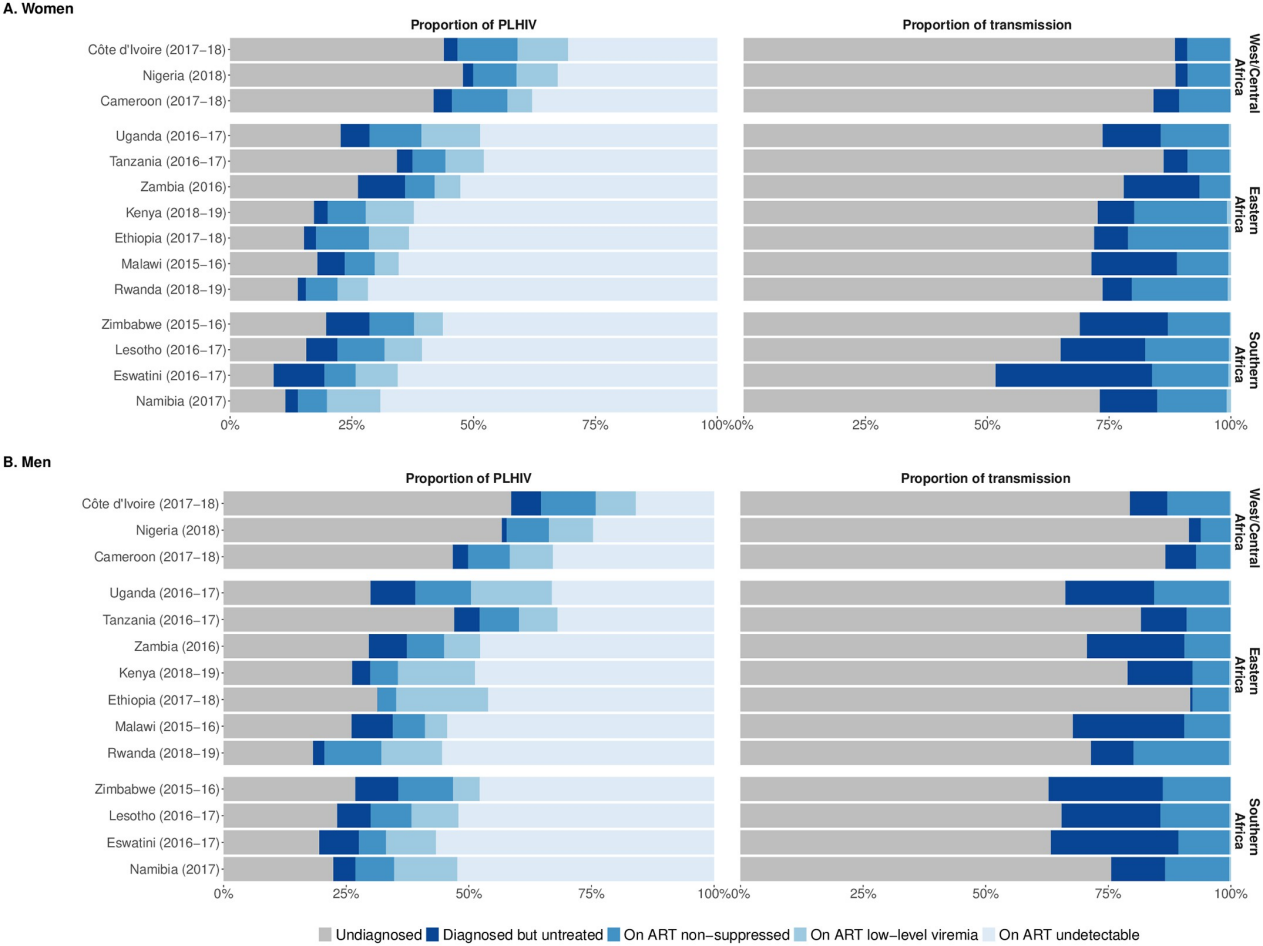
Weighted proportion of PLHIV and estimated proportion of transmission attributed to PLHIV subgroup among (a) women and (b) men across 14 PHIA surveys. Estimated using the country-specific prevalence ratio of HIV high-risk behaviour, proportion size, and transmission rate for each PLHIV subgroup.

In most surveys, the majority of transmission was estimated to be among undiagnosed PLHIV, accounting for the prevalence ratio of HIV high-risk behaviour, distribution of viremia, and relative size of each PLHIV subgroup. The proportion of transmission from this group ranged from 52% (Eswatini, 2016–2017, women) to 92% (Ethiopia, 2017–2018, men; Fig 3). The subgroup with the second largest proportion of transmission varied between PLHIV diagnosed but untreated and those on ART but non-suppressed. Individuals on ART with LLV had the smallest contribution to transmission, ranging between 0.1% (Nigeria, 2018, men) to 0.9% (Namibia, 2017, women; Fig 3).

**Fig 4:**
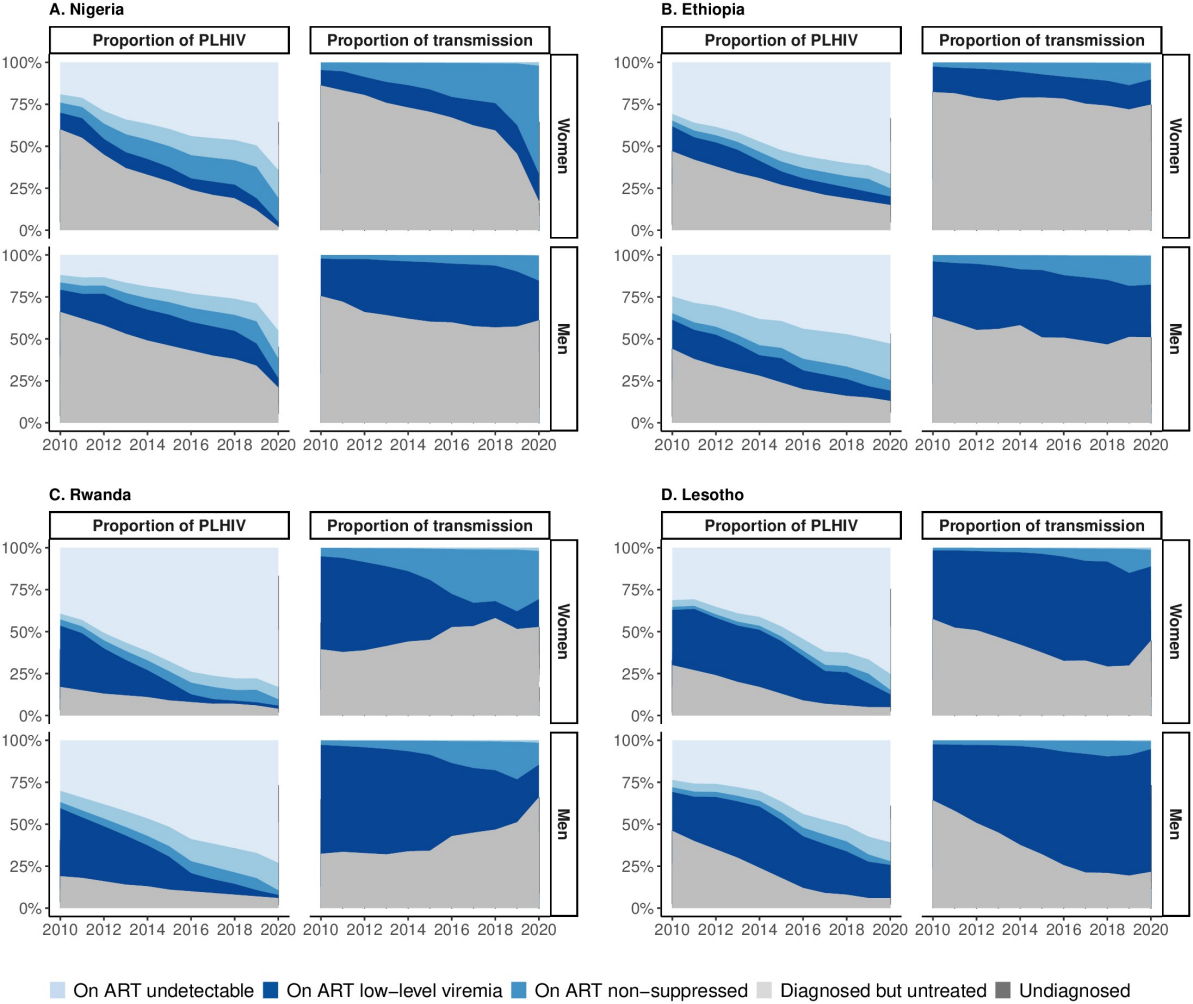
Estimated proportion of PLHIV and transmission proportion attributable to PLHIV subgroups in Nigeria, Ethiopia, Rwanda, Lesotho, 2010–2020. Countries chosen as illustrative examples as their transmission proportion patterns are similar to other countries in their regions. See S12 Fig for all countries in analysis.

Using UNAIDS estimates for the proportion of PLHIV in each subgroup from 2010 to 2020, we estimated that, in 2010, undiagnosed women and men accounted for the majority of transmission (47%–94%) in 9/14 countries (Cote d’Ivoire, Nigeria, Cameroon, Tanzania, Zambia, Ethiopia, Namibia, Lesotho and Zimbabwe), and women and men diagnosed but untreated had the second largest contribution (Fig 4 and S12 Fig). In the remaining countries, diagnosed but untreated PLHIV represented the largest transmission proportion (Kenya, Uganda, Malawi, Rwanda, Eswatini). The estimated transmission proportion from PLHIV on ART but non-suppressed (1%–5%) and with LLV (0.03%–0.2%) was small.

In 2020, the size of the undiagnosed and untreated population reduced and their estimated proportion of tranmsission also reduced since 2010, but, in most countries, the undiagnosed population still accounted for the majority of transmission (17%–75%). However, by 2020 men and women on ART but non-suppressed were the second largest transmission population (5%–64%). In Tanzania, Zambia, Ethiopia, Kenya, Malawi, and Lesotho, men and women undiagnosed and those diagnosed but untreated remained the two largest transmission groups, followed by those on ART but non-suppressed. Transmission from PLHIV on ART with LLV in 2020 remained low (0.3%–2.4%).

In sensitivity analyses, results were similar when we excluded the Nigeria survey, stratified the analysis by region, explored other measures of sexual risk behaviour, assumed equal sexual risk in all groups, higher risk among those on ART vs untreated and used a linear function to represent the relationship between viremia and transmission risk (S13 and S15–S25 Figs). However, when assuming higher levels of sexual risk among PLHIV on ART compared to those not on ART, those undiagnosed and diagnosed but untreated still account for majority of transmission across all countries in our analysis (27%–80% and 3%–32%, respectively), except among women in Ethiopia (2017–2018) and Lesotho (2016–2017), and men in Rwanda (2018–2019), where those on ART but non-suppressed accounted for the largest proportion of estimated transmission (54%, 43% and 48% respectively). Estimated transmission among those with LLV was still low and ranged between 0.3%–1.8% (S22 Fig).

## Discussion

In national HIV surveys with delineation of PLHIV across the HIV care cascade, undiagnosed PLHIV and those diagnosed but untreated were more likely to report risk behaviours that could result in HIV transmission. The distribution of viremia and, consequently, estimated transmission rates were remarkably similar between undiagnosed, diagnosed but untreated, and PLHIV on ART with viral non-suppression. Taken together, we estimated that the majority of transmission continues to be from undiagnosed PLHIV and those diagnosed but untreated, reflecting both higher VL and sexual risk behaviour. Even as ART coverage reaches high levels, timely diagnosis, linkage to care, retention on ART, and effective re-engagement following interruption remain priorities to prevent HIV transmisions.

We estimated that transmission from people with LLV constituted <1% of all new HIV infections. Treated PLHIV with undetectable VL reported the lowest levels of risk behaviour, and we found no evidence of increased risk among those on ART with LLV or virally non-suppressed. This contrasts studies from South Africa and the US where higher rates of HIV risk behaviour among non-suppressed PLHIV on ART were reported, though the authors acknowledged the potential role of unmeasured confounding [15, 22]. Our findings suggest that individuals on ART with LLV or non-suppression are not more likely to engage in HIV high-risk behaviour, which may reflect effective HIV prevention counselling provided during clinical care [33]. Sex and regional differences in the prevalence of HIV risk behaviours were similar to findings from other studies in SSA [15, 24].

Our assumption about infectiousness at LLV was derived by extrapolating the Hill function parameterised by Fraser *et al*. [30] to VL ≤1000 copies/mL, a range in which no transmission was observed in the data used to estimate the function [9]. A new systematic review of linked HIV transmissions did not identify any confirmed transmission events in which the infectious partner had VL ≤1000 copies/mL [34]. Therefore, our assumptions about transmission at LLV are likely conservative, and likely represents an upper bound on the proportion of transmissions. This finding alleviates concerns that people with LLV may become an important transmission sub-population in settings with high treatment coverage and demonstrates the population-level implications of “Undetectable = Untransmittable”. However, studies have found PLHIV with LLV have a high risk of subsequent viral non-suppression [7, 12], which could result in HIV transmission risk and adverse individual health outcomes.

That most transmissions continue to arise from undiagnosed and untreated subgroups was consistent with a recent mathematical modelling study from South Africa, which estimated that, in 2019, 30% of sexually acquired HIV transmissions were from undiagnosed individuals, 60% from diagnosed but untreated, and 10% from non-suppressed persons on ART [35]. The similar viral load distribution between non-suppressed PLHIV on ART and untreated PLHIV was notable. This suggests similar biologic risk of onward transmission among non-suppressed PLHIV on ART as those who are untreated, highlighting the importance of ensuring that individuals achieve and sustain viral suppression. Following large reductions in undiagnosed and untreated PLHIV over the past decade, the contribution to transmission from non-suppressed PLHIV on ART was estimated to have increased (between 1%-5% in 2010 to 5%-65% in 2020). Ongoing adherence support, frequent virologic monitoring, early interventions for viremia, and timely regimen switching among PLHIV on ART are increasingly important in this group. Optimized treatment regimens that increase viral suppression among PLHIV on ART, particularly recent transition to dolutegravir-based regimes [36, 37], will help curtail this source of transmission. The variations in the transmission trends between 2010 and 2020 across countries in our analysis were largely due to differences in distribution across the undiagnosed, diagnosed but untreated, viral non-suppression cascade stages. Since 2020, several countries have repeated PHIA surveys, which have confirmed the increases in ART coverage represented in UNAIDS modelled estimates used here and demonstrated increased viral suppression among persons on ART [38–43].

Strengths of this study included the combination of data on HIV risk behaviour and infectiousness across HIV cascade stages from nationally represntative samples acros multiple countries in SSA. Availability of ARV and VL testing enabled the classification PLHIV by treatment and viremia status. Our study also has limitations. In addition to those identified above, self-reported measures of sexual behaviour are subject to recall and social desirability bias, and we cannot exclude the possibility that ART and its counselling reduced risk reporting more than actual risk taking (and may underestimate relative sexual risk among those on ART). This may have biased our estimates of the prevalence ratios of sexual risk behaviours, however, overall findings about priority transmission subgroups were relatively robust in sensitivity analyses assuming smaller differentials in sexual risk. Second, because the PHIA surveys are cross-sectional, our study is unable to identify changes in sexual behaviours over time and there is a need for further research to understand how these have evolved. Third, we were unable to distinguish between causes for non-suppression while on ART (non-adherence, treatment failure or drug resistance), nor reasons for being untreated (recent diagnosis or interrupting treatment) among those diagnosed but untreated because responses to survey questions about treatment interruptions were redacted from some PHIA survey datasets, and UNAIDS estimates did not stratify the diagnosed but untreated group according to never or previously treated. In the era of “test-and-start”, we expect that persons interrupting treatment account for most transmissions among the diagnosed but not currently on ART population, consistent with findings of the recent mathematical modelling analysis for South Africa [35]. Finally, our findings may not be representative of all countries in the regions included in our analysis due to differences in their HIV epidemiology, healthcare systems and cultural factors. Notably, our results for the Southern African region do not include the national HIV household survey in South Africa [44], which has the largest HIV epidemic in the region. The survey was implemented using different questionnaires than PHIA surveys analysed here and variables could not be sufficiently harmonised for inclusion in pooled analyses.

In conclusion, as HIV treatment coverage reaches high levels in high HIV burden settings in sub-Saharan Africa, HIV programmes require up-to-date information about HIV risk behaviour and transmission risk among subgroups to guide prevention priorities. We estimated that undiagnosed PLHIV and those diagnosed but untreated continue to account for most HIV transmission due to higher levels of both sexual risk behaviour and viral load. Reaching undiagnosed and untreated PLHIV with HIV testing, ART linkage, retention, and treatment support services to attain durable virologic suppression remains the priority to reduce HIV transmission and end the HIV epidemic. As ART coverage increases, individuals on treatment with virologic non-suppression may increasingly contribute to onward transmission and become a high-priority subgroup. Individuals on ART with LLV do not contribute substantially to HIV transmission, but may indicate future transmission risk if LLV predicts future treatment failure and higher viremia.

## Supporting information

S1 Table Summary of participant characteristics across all included PHIA surveys by sex. (DOCX)

S2 Table Crude and adjusted prevalence ratios of self-reporting HIV high-risk behaviour by sex. (DOCX)

S3 Table Crude and adjusted prevalence ratios of self-reporting condomless casual partnership by sex. (DOCX)

S4 Table Crude and adjusted prevalence ratios of self-reporting transactional partnership by sex. (DOCX)

S5 Table Crude and adjusted prevalence ratios of self-reporting condomless last sex (with any partner) by sex. (DOCX)

S6 Table Crude and adjusted prevalence ratios of self-reporting both multiple sexual partnership and condomless last sex (with any partner) by sex. (DOCX)

S7 Table Predicted prevalence ratios of self-reporting high-risk sex relative to PLHIV On ART undetectable (≤50 copies/mL) for each of the 14 survey countries by sex. (DOCX)

S8 Table Predicted prevalence ratios of self-reporting condomless casual partnership for each of the 14 survey countries by sex. (DOCX)

S9 Table Predicted prevalence ratios of self-reporting transactional partnership for each of the 14 survey countries by sex. (DOCX)

S10 Table Predicted prevalence ratios of self-reporting condomless last sex (with any partner) for each of the 14 survey countries by sex. (DOCX)

S11 Table Predicted prevalence ratios of self-reporting both multiple sexual partnership and condomless last sex for each of the 14 survey countries by sex. (DOCX)

S12 Table Mean log10 viral load and transmission rate per year for all PLHIV subgroups by sex. (DOCX)

S1 Fig Conceptual framework. (DOCX)

S2 Fig Flowchart of participants included in study. (DOCX)

S3 Fig Weighted prevalence and 95% confidence intervals of self-reported HIV high-risk behaviour, condomless casual partnerships, transactional partnerships, condomless last sex and both multiple sexual partnership and condomless last sex by sex across 14 PHIA surveys. (DOCX)

S4 Fig Predicted prevalence ratios reporting high-risk sex for each cascade group relative to HIV positive on ART and undetectable (≤50 copies/mL) adults. (DOCX)

S5 Fig Forest plots showing the predicted prevalence ratios and 95% confidence intervals of self-reporting condomless casual partnership for each of the 14 survey countries by sex. (DOCX)

S6 Fig Forest plots showing the predicted prevalence ratios and 95% confidence intervals of self-reporting transactional partnership for each of the 14 survey countries by sex. (DOCX)

S7 Fig Forest plots showing the predicted prevalence ratios and 95% confidence intervals of self-reporting condomless last sex for each of the 14 survey countries by sex. (DOCX)

S8 Fig Forest plots showing the predicted prevalence ratios and 95% confidence intervals of self-reporting both multiple sexual partnership and condomless last sex for each of the 14 survey countries by sex. (DOCX)

S9 Fig Estimated log-odds ratio and 95% confidence intervals of HIV high-risk behaviour, condomless casual partnership, transactional partnership, condomless last sex and self-reporting both multiple sexual partnership and condomless last sex by age predicted by the spline effects by sex and risk behaviour. (DOCX)

S10 Fig (A) Boxplots of the log10 viral load distribution and (B) Mean annual transmission rates and 95% confidence intervals for each PLHIV population sub-group estimated using the Hill function by sex.
(DOCX)

S11 Fig (A) Plot showing the transmission rate as a function of viral load using the Hill function. (B) Distribution of viral load for each PLHIV subgroup using data from Lesotho 2016–2017 survey.
(DOCX)

S12 Fig Estimated proportion of PLHIV in each sub-group and proportion of transmission attributed to each PLHIV sub-group from 2010–2020. (DOCX)

S13 Fig Forest plots showing the adjusted prevalence ratios of self-reporting high HIV high-risk behaviour by sub-groups (sensitivity analyses excluding the Nigeria 2018 survey). (DOCX)

S14 Fig Forest plots showing the adjusted prevalence ratios of self-reporting high HIV high-risk behaviour by sub-groups (including normalized survey weight in regression mode). (DOCX)

S15 Fig Forest plots showing the region-specific adjusted prevalence ratios of self-reporting HIV high-risk behaviour, condomless casual partnerships, transactional partnerships, condomless last sex and self-reporting both multiple sexual partnership and condomless last sex for each subgroup. (DOCX)

S16 Fig Proportion of transmission attributed to PLHIV sub-group by sex across all 14 PHIA surveys (sensitivity analysis using Hill function of transmission and viremia relationship, and region-specific adjusted prevalence ratio of self-reporting HIV high-risk behaviour in transmission equation). (DOCX)

S17 Fig Proportion of transmission attributed to PLHIV sub-group by sex across all 14 PHIA surveys (sensitivity analysis using Hill function of transmission and viremia relationship, and adjusted prevalence ratio of self-reporting condomless casual partnerships in transmission equation). (DOCX)

S18 Fig Proportion of transmission attributed to PLHIV sub-group by sex across all 14 PHIA surveys (sensitivity analysis using Hill function of transmission and viremia relationship, and adjusted prevalence ratio of self-reporting transactional partnerships in transmission equation). (DOCX)

S19 Fig Proportion of transmission attributed to PLHIV sub-group by sex across all 14 PHIA surveys (sensitivity analysis using Hill function of transmission and viremia relationship, and adjusted prevalence ratio of self-reporting condomless last sex (with any partner) in transmission equation). (DOCX)

S20 Fig Proportion of transmission attributed to PLHIV sub-group by sex across all 14 PHIA surveys (sensitivity analysis using Hill function of transmission and viremia relationship, and adjusted prevalence ratio of self-reporting both multiple sexual partnership and condomless last sex in transmission equation). (DOCX)

S21 Fig Proportion of transmission attributed to PLHIV sub-group by sex across all 14 PHIA surveys (sensitivity analysis using Hill function of transmission and viremia relationship, and assuming the same adjusted prevalence ratio of self-reporting HIV high-risk behaviour for all subgroups). (DOCX)

S22 Fig Proportion of transmission attributed to PLHIV sub-group by sex across all 14 PHIA surveys (sensitivity analysis using Hill function of transmission and viremia relationship, and assuming the adjusted prevalence ratio of self-reporting HIV high-risk behaviour among untreated PLHIV was half that among PLHIV on ART). (DOCX)

S23 Fig Plot showing the transmission rate as a function of viral load using the linear function for (A) Women-to-Men and (B) Men-to-Women, and distribution of viral load for each PLHIV subgroup using data from Lesotho 2016–2017 survey for (C) women and (D) men.
(DOCX)

S24 Fig Sex-stratified weighted average transmission rates per sexual contact and 95% confidence intervals for each PLHIV population sub-group estimated using the linear function for each of the 14 PHIA surveys. (DOCX)

S25 Fig Proportion of transmission attributed to PLHIV sub-group by sex across all 14 PHIA surveys (sensitivity analysis using linear function of transmission and viremia relationship, and adjusted prevalence ratio of self-reporting HIV high-risk behaviour in transmission equation. (DOCX)
