# Supplementary material for: HIV risk behaviour, viraemia, and transmission across HIV cascade stages including low-level viremia: Analysis of 14 cross-sectional population-based HIV Impact Assessment surveys in sub-Saharan Africa
Source: PLOS Glob Public Health. 2024 Apr 4;4(4):e0003030. doi: 10.1371/journal.pgph.0003030 (PMC10994324; doi:10.1371/journal.pgph.0003030)
Supplement: S1 Table — (DOCX) [file pgph.0003030.s001.docx]

**S1 Table. Summary of participant characteristics across all included PHIA surveys by sex.** Note: all medians, interquartile ranges and percentages are weighted.

|  | **Western/Central Africa** | | | | | | | **Eastern Africa** | | | | |
| --- | --- | --- | --- | --- | --- | --- | --- | --- | --- | --- | --- | --- |
| **Characteristics** | **Cameroon (2017-2018)** | | **Côte d’Ivoire (2017-2018)** | | | **Nigeria (2018)** | | **Ethiopia (2017-2018)** | | | **Malawi (2015-2016)** | |
|  | **Women**  **(N = 12215)** | **Men**  **(N = 9641)** | **Women**  **(N = 8083)** | | **Men**  **(N = 7610)** | **Women**  **(N = 81992)** | **Men**  **(N = 57860)** | **Women**  **(N = 7860)** | | **Men**  **(N = 4945)** | **Women**  **(N = 8755)** | **Men**  **(N = 6089)** |
| **Age, years** |  |  |  | |  |  |  |  | |  |  |  |
| Range | 15 – 64 | 15 – 64 | 15 – 64 | | 15 – 64 | 15 – 64 | 15 – 64 | 15 – 64 | | 15 – 64 | 15 – 64 | 15 – 64 |
| Median (IQR) | 32 (24 – 42) | 32 (25 – 43) | 30 (23 – 40) | | 32 (25 – 42) | 32 (25 – 42) | 35 (26 – 45) | 31 (25 – 40) | | 33 (27 – 42) | 30 (23 – 40) | 30 (23 – 40) |
| Urban dwelling, n (%) | 4962 (50.7) | 4116 (53.7) | 4151 (62.0) | | 3607 (60.5) | 33518 (46.0) | 23315 (47.0) | 4072 (50.9) | | 2366 (47.3) | 3253 (18.3) | 2236 (20.8) |
| **Wealth quintile, n (%)** | | | |  | | | | |  | | | |
| Lowest | 3713 (21.0) | 2683 (17.8) | 1258 (26.6) | | 929 (23.4) | 15269 (18.6) | 10615 (16.8) | 1362 (16.5) | | 846 (15.7) | 1086 (17.0) | 674 (14.4) |
| Second | 2933 (21.7) | 2168 (19.6) | 1486 (18.9) | | 1225 (18.8) | 16022 (19.3) | 10936 (17.8) | 1380 (17.0) | | 917 (18.1) | 1281 (18.7) | 904 (18.7) |
| Middle | 2200 (20.4) | 1822 (21.3) | 1872 (18.9) | | 1722 (17.9) | 17714 (20.0) | 12064 (18.9) | 1577 (19.6) | | 1082 (21.6) | 1445 (20.5) | 981 (19.8) |
| Fourth | 1718 (17.6) | 1558 (20.3) | 2033 (19.9) | | 2000 (20.3) | 17491 (20.9) | 12396 (21.6) | 1728 (22.3) | | 1092 (22.9) | 1777 (21.1) | 1334 (22.7) |
| Highest | 1644 (19.2) | 1405 (20.9) | 1434 (15.7) | | 1734 (19.5) | 15496 (21.2) | 11849 (25.0) | 1813 (24.6) | | 1008 (21.7) | 3166 (22.6) | 2196 (24.4) |
| Missing | 7 (0.1) | 5 (0.1) | 0 (0.0) | | 0 (0.0) | 0 (0.0) | 0 (0.0) | 0 (0.0) | | 0 (0.0) | 0 (0.0) | 0 (0.0) |
| **Level of education, n (%)** | | | |  | | | | |  | | | |
| None | 3191 (19.7) | 1235 (9.0) | 4238 (49.3) | | 3130 (37.3) | 28716 (36.5) | 11735 (19.4) | 1671 (22.0) | | 355 (6.8) | 949 (13.6) | 314 (5.5) |
| Primary | 3906 (31.0) | 2755 (25.4) | 2245 (27.5) | | 2016 (24.3) | 17523 (20.4) | 11300 (17.8) | 3033 (38.8) | | 1754 (35.7) | 5317 (65.5) | 3328 (60.4) |
| Secondary | 3128 (28.4) | 2914 (30.5) | 1346 (18.9) | | 1954 (28.6) | 26306 (31.8) | 22570 (41.4) | 1726 (21.8) | | 1282 (25.8) | 2156 (18.9) | 2078 (30.1) |
| More than secondary | 1972 (20.7) | 2713 (34.8) | 241 (4.2) | | 499 (9.6) | 9360 (11.1) | 12222 (21.3) | 1399 (16.9) | | 1537 (31.4) | 328 (2.0) | 368 (4.0) |
| Missing | 18 (0.2) | 24 (0.3) | 13 (0.1) | | 11 (0.2) | 87 (0.1) | 33 (0.1) | 31 (0.5) | | 17 (0.3) | 5 (<0.1) | 1 (<0.1) |
| **Marital status, n (%)** | | | |  | | | | |  | | | |
| Currently married | 7672 (60.8) | 5778 (55.9) | 5247 (61.6) | | 4592 (55.2) | 7987 (75.4) | 6720 (70.5) | 5161 (65.5) | | 3519 (72.3) | 6126 (69.5) | 4047 (67.1) |
| Never married | 2240 (20.4) | 2839 (33.8) | 2057 (27.4) | | 2598 (39.1) | 2468 (12.7) | 3212 (26.5) | 822 (9.7) | | 1083 (21.3) | 1029 (11.8) | 1695 (27.5) |
| Divorced/separated | 1269 (10.7) | 890 (9.1) | 403 (5.6) | | 344 (4.7) | 1320 (3.6) | 348 (2.1) | 1232 (15.9) | | 288 (5.4) | 1108 (13.2) | 294 (4.7) |
| Widower/widow | 1008 (7.9) | 116 (1.0) | 345 (4.9) | | 52 (0.6) | 1155 (8.2) | 72 (0.8) | 625 (8.7) | | 47 (0.8) | 483 (5.3) | 48 (0.6) |
| Missing | 26 (0.2) | 18 (0.2) | 31 (0.5) | | 24 (0.4) | 8 (0.1) | 4 (0.1) | 20 (0.2) | | 8 (0.2) | 9 (0.1) | 5 (0.1) |
| **Pregnancy status, n (%)** | | | |  | | | | |  | | | |
| Pregnant | 1109 (8.2) | - | 806 (9.6) | | - | 6925 (8.8) | - | 560 (6.2) | | - | 547 (6.5) | - |
| Not pregnant | 10897 (90.2) | - | 7184 (89.1) | | - | 73986 (89.9) | - | 7251 (93.1) | | - | 8112 (92.4) | - |
| Missing | 209 (1.6) | - | 93 (1.3) | | - | 1081 (1.3) | - | 49 (0.7) | | - | 96 (1.1) | - |
| **HIV/ART/viremia status, n (%)** | | | |  | | | | |  | | | |
| HIV negative | 11570 (94.5) | 9368 (97.3) | 7801 (95.9) | | 7493 (98.2) | 80258 (98.0) | 57111 (98.7) | 7464 (95.0) | | 4822 (97.6) | 7338 (86.4) | 5438 (90.9) |
| On ART undetectable | 231 (2.1) | 88 (0.9) | 88 (1.3) | | 22 (0.3) | 600 (0.6) | 215 (0.3) | 250 (3.1) | | 58 (1.1) | 914 (8.9) | 361 (4.9) |
| On ART low-level viremia | 40 (0.3) | 20 (0.2) | 31 (0.4) | | 16 (0.2) | 140 (0.2) | 73 (0.1) | 32 (0.4) | | 24 (0.4) | 60 (0.7) | 24 (0.4) |
| On ART non-suppressed | 71 (0.6) | 28 (0.2) | 27 (0.5) | | 15 (0.2) | 168 (0.2) | 70 (0.1) | 43 (0.5) | | 7 (0.1) | 88 (0.8) | 41 (0.6) |
| Diagnosed but untreated | 24 (0.2) | 9 (<0.1) | 11 (0.1) | | 3 (0.1) | 33 (0.1) | 9 (<0.1) | 9 (0.1) | | 1 (<0.1) | 95 (0.8) | 52 (0.8) |
| Undiagnosed | 279 (2.3) | 128 (1.3) | 125 (1.8) | | 61 (1.0) | 793 (0.9) | 382 (0.7) | 62 (0.8) | | 33 (0.7) | 260 (2.4) | 173 (2.4) |
| **HIV high-risk behaviour, n (%)** | | | |  | | | | |  | | | |
| No | 11903 (97.2) | 8775 (90.4) | 7874 (96.9) | | 7081 (92.2) | 80047 (97.5) | 52352 (98.3) | 7787 (99.2) | | 4808 (97.2) | 8656 (99.0) | 5766 (94.4) |
| Yes | 312 (2.8) | 866 (9.6) | 209 (3.1) | | 529 (7.8) | 1945 (2.5) | 5508 (10.7) | 73 (0.8) | | 137 (2.8) | 99 (1.0) | 323 (5.6) |
| **Condomless casual partnerships, n (%)** | | | |  | | | | |  | | | |
| No | 11554 (94.0) | 8620 (88.5) | 7880 (97.2) | | 7178 (93.4) | 77725 (95.0) | 50305 (85.6) | 7722 (98.4) | | 4784 (96.8) | 8707 (99.4) | 5903 (96.8) |
| Yes | 661 (6.0) | 1021 (11.5) | 203 (2.8) | | 432 (6.6) | 4267 (5.0) | 7555 (14.4) | 138 (1.6) | | 161 (3.1) | 48 (0.6) | 186 (3.2) |
| **Transactional partnerships, n (%)** | | | |  | | | | |  | | | |
| No | 11452 (93.7) | 9280 (96.2) | 7764 (95.9) | | 7390 (97.2) | 73347 (89.5) | 54584 (93.9) | 7582 (96.7) | | 4806 (97.3) | 7772 (88.2) | 5809 (95.2) |
| Yes | 649 (5.4) | 310 (3.2) | 307 (4.0) | | 207 (2.7) | 8133 (9.8) | 2885 (5.4) | 238 (2.9) | | 96 (2.0) | 955 (11.4) | 176 (3.1) |
| Missing | 114 (0.9) | 51 (0.6) | 12 (0.1) | | 13 (0.1) | 513 (0.7) | 391 (0.7) | 40 (0.4) | | 43 (0.7) | 28 (0.4) | 104 (1.7) |
| **Condomless last sex, n (%)** |  |  |  | |  |  |  |  | |  |  |  |
| No | 3318 (28.9) | 2771 (31.7) | 2159 (29.2) | | 2256 (31.9) | 21676 (24.9) | 18300 (31.5) | 2909 (38.1) | | 1565 (30.5) | 2733 (30.6) | 2115 (33.7) |
| Yes | 8666 (69.0) | 6042 (58.7) | 5653 (66.7) | | 4607 (56.6) | 60308 ((75.1) | 39532 (68.5) | 4907 (61.4) | | 3275 (67.5) | 6022 (69.4) | 3971 (66.3) |
| Missing | 231 (2.1) | 828 (9.6) | 271 (4.1) | | 747 (11.4) | 8 (<0.1) | 28 (<0.1) | 44 (0.5) | | 105 (2.0) | 0 (0.0) | 3 (<0.1) |
| **Multiple sexual partnership and condomless last sex, n (%)** | | | | | | | | | | | | |
| No | 11720 (95.4) | 7382 (77.0) | 7576 (92.0) | | 5560 (72.3) | 79650 (96.9) | 46307 (79.2) | 7730 (98.4) | | 4694 (95.0) | 8542 (97.8) | 5186 (84.9) |
| Yes | 264 (2.5) | 1431 (13.4) | 236 (3.8) | | 1303 (16.3) | 2334 (3.1) | 11525 (20.8) | 86 (1.1) | | 146 (3.0) | 213 (2.2) | 900 (15.1) |
| Missing | 231 (2.1) | 828 (9.6) | 271 (4.1) | | 747 (11.4) | 8 (<0.1) | 28 (<0.1) | 44 (0.5) | | 105 (2.0) | 0 (0.0) | 3 (<0.1) |

**Table S1 continued**

|  | **Eastern Africa** | | | | | | | | | | |  |  |  |  |  |  |  |  |
| --- | --- | --- | --- | --- | --- | --- | --- | --- | --- | --- | --- | --- | --- | --- | --- | --- | --- | --- | --- |
| **Characteristics** | **Rwanda (2018-2019)** | | | **Tanzania (2016-2017)** | | **Uganda (2016-2017)** | | **Zambia (2016)** | | **Kenya (2018-2019)** | |  |  |  |  |  |  |  |  |
|  | **Women**  **(N = 12938)** | | **Men**  **(N = 10356)** | **Women**  **(N = 15554)** | **Men**  **(N = 11632)** | **Women**  **(N = 14623)** | **Men**  **(N = 10459)** | **Women**  **(N = 9110)** | **Men**  **(N = 6438)** | **Women**  **(N = 13189)** | **Men**  **(N = 9017)** |  |  |  |  |  |  |  |  |
| **Age, year** |  | |  |  |  |  |  |  |  |  |  |  |  |  |  |  |  |  |  |
| Range | 15 – 64 | | 15 – 64 | 15 – 80 | 15 – 80 | 15 – 64 | 15 – 64 | 15 – 59 | 15 – 59 | 15 – 64 | 15 – 64 |  |  |  |  |  |  |  |  |
| Median (IQR) | 34 (27 – 45) | | 33 (26 – 43) | 32 (24 – 45) | 32 (24 – 45) | 29 (23 – 40) | 30 (22 – 40) | 29 (23 – 39) | 30 (23 – 40) | 31 (24 – 41) | 32 (24 – 42) |  |  |  |  |  |  |  |  |
| Urban dwelling, n (%) | 3095 (19.2) | | 2829 (22.2) | 5173 (37.4) | 3430 (35.2) | 4022 (29.1) | 2667 (27.5) | 4054 (45.1) | 2516 (43.7) | 4839 (37.4) | 3355 (35.2) |  |  |  |  |  |  |  |  |
| **Wealth quintile, n (%)** | | | | | | | | | |  |  |  |  |  |  |  |  |  |  |
| Lowest | 2454 (20.7) | | 1560 (16.6) | 3407 (19.7) | 2659 (19.6) | 3949 (20.3) | 2866 (21.0) | 1463 (16.1) | 1030 (14.9) | 3555 (20.3) | 2141 (17.8) |  |  |  |  |  |  |  |  |
| Second | 2449 (20.0) | | 1740 (18.1) | 3346 (20.5) | 2679 (21.5) | 2864 (19.3) | 2208 (20.3) | 1668 (18.4) | 1284 (18.3) | 2912 (21.6) | 1997 (20.6) |  |  |  |  |  |  |  |  |
| Middle | 2438 (19.9) | | 1959 (20.0) | 3477 (20.7) | 2581 (20.8) | 2767 (20.3) | 1964 (19.7) | 1853 (19.9) | 1413 (20.9) | 2727 (20.4) | 1995 (21.8) |  |  |  |  |  |  |  |  |
| Fourth | 2479 (20.0) | | 2141 (21.5) | 2797 (19.3) | 1977 (18.9) | 2402 (19.9) | 1651 (19.2) | 2046 (22.2) | 1316 (21.7) | 2351 (19.2) | 1794 (21.2) |  |  |  |  |  |  |  |  |
| Highest | 3113 (19.4) | | 2944 (23.8) | 2522 (19.8) | 1734 (19.2) | 2641 (20.2) | 1770 (19.8) | 2043 (23.0) | 1363 (23.8) | 1642 (18.5) | 1090 (18.6) |  |  |  |  |  |  |  |  |
| Missing | 5 (<0.1) | | 12 (<0.1) | 5 (<0.1) | 2 (<0.1) | 0 (0.0) | 0 (0.0) | 37 (0.4) | 32 (0.4) | 2 (<0.1) | 0 (0.0) |  |  |  |  |  |  |  |  |
| **Level of education, n (%)** | | | | | | | | | |  |  |  |  |  |  |  |  |  |  |
| None | 1784 (14.3) | | 912 (9.3) | 3782 (21.6) | 1550 (11.5) | 2098 (12.1) | 486 (4.0) | 600 (6.8) | 188 (3.0) | 1755 (9.3) | 531 (3.7) |  |  |  |  |  |  |  |  |
| Primary | 8161 (64.7) | | 6552 (64.7) | 9266 (60.5) | 7668 (64.4) | 8447 (57.1) | 5788 (54.2) | 4495 (48.9) | 2480 (36.7) | 6567 (47.4) | 3967 (40.8) |  |  |  |  |  |  |  |  |
| Secondary | 2455 (17.6) | | 2245 (20.6) | 2371 (16.8) | 2148 (21.4) | 2906 (22.0) | 2650 (26.0) | 3370 (36.9) | 3111 (48.8) | 2907 (25.8) | 2358 (28.7) |  |  |  |  |  |  |  |  |
| More than secondary | 523 (3.3) | | 644 (5.4) | 126 (1.0) | 260 (2.7) | 1094 (8.3) | 1464 (15.0) | 642 (7.4) | 656 (11.5) | 1043 (9.3) | 987 (12.3) |  |  |  |  |  |  |  |  |
| Missing | 15 (0.1) | | 3 (<0.1) | 9 (<0.1) | 6 (<0.1) | 78 (0.5) | 71 (0.8) | 3 (<0.1) | 3 (<0.1) | 917 (8.2) | 1174 (14.5) |  |  |  |  |  |  |  |  |
| **Marital status, n (%)** | | | | | | | | | |  |  |  |  |  |  |  |  |  |  |
| Currently married | 7987 (62.5) | | 6720 (66.2) | 10236 (64.0) | 7983 (65.4) | 9496 (63.7) | 6717 (61.1) | 5988 (64.7) | 3970 (59.2) | 8860 (65.6) | 5692 (59.3) |  |  |  |  |  |  |  |  |
| Never married | 2468 (17.7) | | 3212 (29.5) | 1865 (14.0) | 2644 (26.5) | 1870 (14.3) | 2734 (29.1) | 1656 (19.6) | 2106 (35.5) | 2086 (18.8) | 2559 (32.8) |  |  |  |  |  |  |  |  |
| Divorced/separated | 1320 (10.6) | | 348 (3.6) | 1898 (12.2) | 782 (6.6) | 2208 (15.3) | 892 (8.9) | 971 (10.6) | 309 (4.6) | 1191 (9.0) | 632 (6.9) |  |  |  |  |  |  |  |  |
| Widower/widow | 1155 (9.1) | | 72 (0.7) | 1543 (9.7) | 213 (1.4) | 1005 (6.3) | 99 (0.7) | 495 (5.1) | 53 (0.7) | 1030 (6.3) | 127 (0.9) |  |  |  |  |  |  |  |  |
| Missing | 8 (0.1) | | 4 (<0.1) | 12 (0.1) | 10 (0.1) | 44 (0.4) | 17 (0.2) | 0 (0.0) | 0 (0.0) | 22 (0.2) | 7 (<0.1) |  |  |  |  |  |  |  |  |
| **Pregnancy status, n (%)** | | | | | | | | | |  |  |  |  |  |  |  |  |  |  |
| Pregnant | 975 (7.6) | | - | 1276 (8.1) | - | 1390 (9.6) | - | 759 (8.8) | - | 849 (6.7) | - |  |  |  |  |  |  |  |  |
| Not pregnant | 11815 (91.2) | | - | 14092 (90.8) | - | 12996 (88.6) | - | 8241 (89.8) | - | 12230 (92.3) | - |  |  |  |  |  |  |  |  |
| Missing | 148 (1.2) | | - | 186 (1.1) | - | 237 (1.8) | - | 110 (1.4) | - | 110 (1.0) | - |  |  |  |  |  |  |  |  |
| **HIV/ART/viremia status, n (%)** | | | | | | | | | |  |  |  |  |  |  |  |  |  |  |
| HIV negative | 12334 (95.5) | | 10073 (97.3) | 14388 (93.4) | 11116 (96.3) | 13490 (91.6) | 9934 (94.8) | 7609 (84.1) | 5773 (90.0) | 12194 (92.6) | 8669 (96.7) |  |  |  |  |  |  |  |  |
| On ART undetectable | 436 (3.2) | | 159 (1.5) | 550 (3.2) | 172 (1.2) | 552 (4.1) | 174 (1.7) | 817 (8.4) | 316 (4.7) | 621 (4.6) | 186 (1.6) |  |  |  |  |  |  |  |  |
| On ART low-level viremia | 39 (0.3) | | 35 (0.3) | 89 (0.5) | 42 (0.3) | 144 (1.0) | 90 (0.8) | 88 (0.8) | 53 (0.7) | 100 (0.7) | 51 (0.5) |  |  |  |  |  |  |  |  |
| On ART non-suppressed | 36 (0.3) | | 32 (0.3) | 85 (0.4) | 40 (0.3) | 129 (0.9) | 62 (0.6) | 93 (1.0) | 50 (0.8) | 74 (0.6) | 18 (0.2) |  |  |  |  |  |  |  |  |
| Diagnosed but untreated | | 12 (0.1) | 8 (0.1) | 45 (0.2) | 28 (0.2) | 66 (0.5) | 48 (0.5) | 136 (1.5) | 54 (0.8) | 27 (0.2) | 13 (0.1) |  |  |  |  |  |  |  |  |
| Undiagnosed | 81 (0.6) | | 49 (0.5) | 397 (2.3) | 234 (1.7) | 242 (1.9) | 151 (1.6) | 367 (4.2) | 192 (3.0) | 173 (1.3) | 80 (0.9) |  |  |  |  |  |  |  |  |
| **HIV high-risk behaviour, n (%)** | | | | | | | | | |  |  |  |  |  |  |  |  |  |  |
| No | 12495 (96.8) | | 9452 (91.4) | 14726 (94.3) | 9866 (83.0) | 14260 (97.3) | 9268 (88.0) | 8944 (98.2) | 5927 (91.7) | 13004 (98.5) | 8472 (93.1) |  |  |  |  |  |  |  |  |
| Yes | 443 (3.2) | | 904 (8.6) | 828 (5.7) | 1766 (17.0) | 363 (2.7) | 1191 (12.0) | 166 (1.8) | 511 (8.3) | 185 (1.5) | 545 (6.9) |  |  |  |  |  |  |  |  |
| **Condomless casual partnerships, n (%)** | | | | | | | | | |  |  |  |  |  |  |  |  |  |  |
| No | 11752 (91.4) | | 8947 (86.7) | 13999 (88.9) | 9390 (78.4) | 13807 (93.9) | 9028 (85.4) | 8696 (95.2) | 5685 (87.2) | 12485 (93.8) | 8184 (90.0) |  |  |  |  |  |  |  |  |
| Yes | 1186 (8.6) | | 1409 (13.3) | 1555 (11.1) | 2242 (21.6) | 816 (6.1) | 1431 (14.6) | 414 (4.8) | 753 (12.8) | 704 (6.2) | 833 (10.0) |  |  |  |  |  |  |  |  |
| **Transactional partnerships, n (%)** | | | | | | | | | |  |  |  |  |  |  |  |  |  |  |
| No | 12234 (94.7) | | 10045 (97.0) | 14310 (91.4) | 11312 (97.2) | 12216 (83.7) | 9784 (93.9) | 7928 (87.0) | 6182 (96.2) | 12108 (91.5) | 8664 (95.7) |  |  |  |  |  |  |  |  |
| Yes | 687 (5.2) | | 172 (1.7) | 1185 (8.2) | 226 (2.0) | 2360 (16.0) | 635 (5.7) | 1167 (12.8) | 225 (3.3) | 1031 (8.0) | 310 (3.8) |  |  |  |  |  |  |  |  |
| Missing | 17 (0.1) | | 139 (1.3) | 59 (0.4) | 94 (0.8) | 47 (0.3) | 40 (0.4) | 15 (0.2) | 31 (0.5) | 50 (0.5) | 43 (0.5) |  |  |  |  |  |  |  |  |
| **Condomless last sex, n (%)** | | | | | | | | | | | |  |  |  |  |  |  |  |  |
| No | 4107 (31.4) | | 3055 (28.6) | 5186 (33.0) | 3348 (28.5) | 4082 (27.7) | 2903 (28.7) | 2931 (32.5) | 2491 (39.9) | 3954 (29.6) | 2838 (33.4) |  |  |  |  |  |  |  |  |
| Yes | 8558 (66.7) | | 6595 (64.9) | 9957 (64.2) | 7248 (62.7) | 10524 (72.2) | 7490 (70.7) | 6174 (67.4) | 3945 (60.1) | 9085 (69.2) | 5704 (60.1) |  |  |  |  |  |  |  |  |
| Missing | 273 (1.9) | | 706 (6.5) | 411 (2.8) | 1036 (8.8) | 17 (0.1) | 66 (0.6) | 5 (0.1) | 2 (<0.1) | 150 (1.2) | 475 (5.9) |  |  |  |  |  |  |  |  |
| **Multiple sexual partnership and condomless last sex, n (%)** | | | | | | | | | | | |  |  |  |  |  |  |  |  |
| No | 12400 (96.1) | | 8960 (86.6) | 14515 (92.8) | 8597 (73.3) | 14116 (96.4) | 8042 (77.2) | 8918 (97.8) | 5596 (87.1) | 12884 (97.8) | 7889 (87.3) |  |  |  |  |  |  |  |  |
| Yes | 265 (2.0) | | 690 (6.9) | 628 (4.4) | 1999 (17.8) | 490 (3.5) | 2351 (22.1) | 187 (2.1) | 840 (12.9) | 155 (1.2) | 653 (6.7) |  |  |  |  |  |  |  |  |
| Missing | 273 (1.9) | | 706 (6.5) | 411 (2.8) | 1036 (8.9) | 17 (0.1) | 66 (0.6) | 5 (0.1) | 2 (<0.1) | 150 (1.2) | 475 (5.9) |  |  |  |  |  |  |  |  |

**Table S1 continued**

|  | **Southern Africa** | | | | | | | |  |  |  |  |  |  |
| --- | --- | --- | --- | --- | --- | --- | --- | --- | --- | --- | --- | --- | --- | --- |
| **Characteristics** | **Lesotho (2016-2017)** | | **Namibia (2017)** | | **Zimbabwe (2015-2016)** | | **Eswatini (2016-2017)** | |  |  |  |  |  |  |
|  | **Women**  **(N = 5962)** | **Men**  **(N = 3985)** | **Women**  **(N = 7560)** | **Men**  **(N = 5561)** | **Women**  **(N = 11079)** | **Women**  **(N = 7169)** | **Women**  **(N = 5385)** | **Men**  **(N = 3306)** |  |  |  |  |  |  |
| **Age, year** |  |  |  |  |  |  |  |  |  |  |  |  |  |  |
| Range | 15 – 59 | 15 – 59 | 15 – 64 | 15 – 64 | 15 – 80 | 15 – 80 | 15 – 80 | 15 – 80 |  |  |  |  |  |  |
| Median (IQR) | 32 (24 – 42) | 31 (23 – 40) | 32 (24 – 43) | 31 (24 – 41) | 33 (25 – 45) | 34 (25 – 45) | 34 (26 – 45) | 33 (25 – 45) |  |  |  |  |  |  |
| Urban dwelling, n (%) | 2333 (42.8) | 1456 (40.3) | 3335 (60.3) | 2384 (61.1) | 3349 (35.0) | 1728 (33.3) | 1120 (27.8) | 815 (29.8) |  |  |  |  |  |  |
| **Wealth quintile, n (%)** | | | | | | |  |  |  |  |  |  |  |  |
| Lowest | 1203 (17.3) | 814 (17.3) | 1973 (18.3) | 1312 (16.3) | 2652 (20.1) | 1748 (19.3) | 1287 (20.7) | 705 (18.9) |  |  |  |  |  |  |
| Second | 1175 (17.8) | 866 (20.1) | 1693 (18.6) | 1369 (19.8) | 2376 (20.3) | 1614 (20.0) | 1159 (20.1) | 699 (19.6) |  |  |  |  |  |  |
| Middle | 1173 (19.8) | 778 (19.8) | 1619 (20.9) | 1319 (23.4) | 2127 (19.2) | 1596 (21.2) | 1279 (22.6) | 766 (22.7) |  |  |  |  |  |  |
| Fourth | 1177 (20.9) | 792 (21.1) | 1332 (21.6) | 918 (21.1) | 1822 (19.2) | 1105 (19.5) | 832 (17.6) | 559 (18.4) |  |  |  |  |  |  |
| Highest | 1218 (23.9) | 728 (21.5) | 943 (20.6) | 643 (19.4) | 2102 (21.2) | 1106 (19.9) | 826 (18.9) | 577 (20.4) |  |  |  |  |  |  |
| Missing | 16 (0.3) | 7 (0.2) | 0 (0.0) | 0 (0.0) | 0 (0.0) | 0 (0.0) | 2 (<0.1) | 0 (0.0) |  |  |  |  |  |  |
| **Level of education, n (%)** | | | | | | |  |  |  |  |  |  |  |  |
| None | 94 (1.4) | 400 (8.6) | 557 (5.4) | 621 (8.0) | 606 (4.8) | 165 (1.7) | 493 (6.7) | 225 (5.5) |  |  |  |  |  |  |
| Primary | 2608 (41.1) | 1734 (40.8) | 2040 (21.4) | 1673 (23.0) | 4148 (33.8) | 2353 (26.9) | 1691 (27.1) | 1045 (28.3) |  |  |  |  |  |  |
| Secondary | 2690 (45.7) | 1478 (38.7) | 4337 (59.9) | 2820 (55.4) | 5792 (55.6) | 4059 (60.4) | 1496 (28.5) | 781 (23.0) |  |  |  |  |  |  |
| More than secondary | 568 (11.7) | 369 (11.7) | 606 (13.1) | 434 (13.4) | 524 (5.7) | 580 (10.9) | 1695 (37.5) | 1248 (43.1) |  |  |  |  |  |  |
| Missing | 2 (0.1) | 4 (0.1) | 20 (0.2) | 13 (0.2) | 9 (0.1) | 12 (0.1) | 10 (0.2) | 7 (0.2) |  |  |  |  |  |  |
| **Marital status, n (%)** | | | | | | |  |  |  |  |  |  |  |  |
| Currently married | 3520 (59.2) | 1911 (49.3) | 2917 (35.9) | 2229 (37.6) | 7401 (68.3) | 4960 (68.2) | 2462 (46.3) | 1608 (46.3) |  |  |  |  |  |  |
| Never married | 1220 (21.2) | 1634 (40.7) | 3747 (53.0) | 2875 (54.4) | 993 (8.6) | 1654 (24.6) | 1832 (37.0) | 1443 (46.7) |  |  |  |  |  |  |
| Divorced/separated | 434 (7.3) | 279 (6.5) | 541 (7.0) | 376 (6.7) | 1059 (10.0) | 357 (5.0) | 261 (5.1) | 160 (4.6) |  |  |  |  |  |  |
| Widower/widow | 783 (12.2) | 152 (3.3) | 293 (3.5) | 47 (0.7) | 1615 (13.0) | 191 (2.1) | 803 (11.0) | 81 (1.9) |  |  |  |  |  |  |
| Missing | 5 (0.1) | 9 (0.2) | 62 (0.6) | 34 (0.6) | 11 (0.1) | 7 (0.1) | 27 (0.6) | 14 (0.4) |  |  |  |  |  |  |
| **Pregnancy status, n (%)** | | | | | | |  |  |  |  |  |  |  |  |
| Pregnant | 271 (4.6) | - | 534 (6.8) | - | 603 (6.0) | - | 190 (3.8) | - |  |  |  |  |  |  |
| Not pregnant | 5670 (95.0) | - | 6976 (92.6) | - | 10293 (92.1) | - | 5140 (95.1) | - |  |  |  |  |  |  |
| Missing | 21 (0.4) | - | 50 (0.6) | - | 183 (1.9) | - | 55 (1.1) | - |  |  |  |  |  |  |
| **HIV/ART/viremia status, n (%)** | | | | | | |  |  |  |  |  |  |  |  |
| HIV negative | 3906 (66.6) | 3088 (78.3) | 6205 (83.9) | 4965 (90.3) | 8935 (82.7) | 6089 (86.7) | 3466 (63.2) | 2422 (75.5) |  |  |  |  |  |  |
| On ART undetectable | 1267 (20.2) | 478 (11.3) | 935 (11.1) | 317 (5.1) | 1262 (9.8) | 548 (6.3) | 1288 (24.1) | 528 (13.9) |  |  |  |  |  |  |
| On ART low-level viremia | 163 (2.6) | 88 (2.1) | 152 (1.8) | 82 (1.2) | 131 (1.0) | 64 (0.7) | 156 (3.2) | 92 (2.5) |  |  |  |  |  |  |
| On ART non-suppressed | 200 (3.2) | 73 (1.8) | 97 (0.9) | 53 (0.8) | 188 (1.6) | 111 (1.5) | 124 (2.3) | 45 (1.3) |  |  |  |  |  |  |
| Diagnosed but untreated | 124 (2.1) | 60 (1.5) | 39 (0.4) | 23 (0.4) | 184 (1.5) | 92 (1.2) | 193 (3.8) | 69 (2.0) |  |  |  |  |  |  |
| Undiagnosed | 302 (5.2) | 198 (5.0) | 132 (1.9) | 121 (2.2) | 379 (3.4) | 265 (3.6) | 158 (3.3) | 150 (4.8) |  |  |  |  |  |  |
| **HIV high-risk behaviour, n (%)** | | | | | | |  |  |  |  |  |  |  |  |
| No | 5814 (97.4) | 3711 (93.2) | 7447 (98.3) | 5272 (94.7) | 10934 (98.6) | 6801 (94.5) | 5323 (98.7) | 3239 (97.9) |  |  |  |  |  |  |
| Yes | 148 (2.6) | 274 (6.8) | 113 (1.7) | 289 (5.3) | 145 (1.4) | 368 (5.5) | 62 (1.3) | 67 (2.1) |  |  |  |  |  |  |
| **Condomless casual partnerships, n (%)** | | | | | | |  |  |  |  |  |  |  |  |
| No | 5766 (96.6) | 3734 (93.8) | 7459 (98.6) | 5383 (96.9) | 10947 (98.7) | 6889 (95.8) | 5353 (99.4) | 3270 (98.9) |  |  |  |  |  |  |
| Yes | 196 (3.4) | 251 (6.2) | 101 (1.4) | 178 (3.1) | 132 (1.3) | 280 (4.2) | 32 (0.6) | 36 (1.1) |  |  |  |  |  |  |
| **Transactional partnerships, n (%)** | | | | | | |  |  |  |  |  |  |  |  |
| No | 5674 (95.2) | 3890 (97.5) | 6995 (92.5) | 5347 (95.6) | 10235 (92.5) | 6929 (96.7) | 5183 (96.0) | 3235 (97.8) |  |  |  |  |  |  |
| Yes | 277 (4.6) | 88 (2.3) | 545 (7.2) | 208 (4.3) | 805 (7.1) | 160 (2.1) | 189 (3.8) | 65 (2.0) |  |  |  |  |  |  |
| Missing | 11 (0.2) | 7 (0.2) | 20 (0.3) | 6 (0.1) | 39 (0.4) | 80 (1.2) | 13 (0.2) | 6 (0.2) |  |  |  |  |  |  |
| **Condomless last sex, n (%)** | | | | | | | | |  |  |  |  |  |  |
| No | 3184 (53.3) | 2458 (61.7) | 3861 (53.0) | 2826 (51.7) | 4366 (37.1) | 2955 (40.3) | 3486 (63.5) | 2053 (61.6) |  |  |  |  |  |  |
| Yes | 2765 (46.4) | 1509 (37.8) | 3555 (44.9) | 2295 (39.4) | 6713 (62.9) | 4209 (59.6) | 1879 (36.1) | 1205 (36.9) |  |  |  |  |  |  |
| Missing | 13 (0.2) | 18 (0.5) | 144 (2.1) | 440 (8.9) | 0 (0.0) | 5 (0.1) | 20 (0.4) | 48 (1.4) |  |  |  |  |  |  |
| **Multiple sexual partnership and condomless last sex, n (%)** | | | | | | | | |  |  |  |  |  |  |
| No | 5635 (94.5) | 3551 (88.3) | 7314 (96.4) | 4887 (86.9) | 10888 (98.3) | 6329 (87.8) | 5277 (97.8) | 3039 (91.6) |  |  |  |  |  |  |
| Yes | 314 (5.3) | 436 (11.3) | 102 (1.5) | 234 (4.2) | 191 (17.1) | 835 (12.1) | 88 (1.8) | 219 (6.9) |  |  |  |  |  |  |
| Missing | 13 (0.2) | 18 (0.5) | 144 (2.1) | 440 (8.9) | 0 (0.0) | 5 (0.1) | 20 (0.4) | 48 (1.4) |  |  |  |  |  |  |
