# Supplementary material for: HIV risk behaviour, viraemia, and transmission across HIV cascade stages including low-level viremia: Analysis of 14 cross-sectional population-based HIV Impact Assessment surveys in sub-Saharan Africa
Source: PLOS Glob Public Health. 2024 Apr 4;4(4):e0003030. doi: 10.1371/journal.pgph.0003030 (PMC10994324; doi:10.1371/journal.pgph.0003030)
Supplement: S2 Table — (DOCX) [file pgph.0003030.s002.docx]

**S2 Table. Crude and adjusted prevalence ratios of self-reporting HIV high-risk behaviour by sex.** Models were adjusted for age, level of education, wealth quintile, marital status, urban/rural dwelling or urbanicity size and pregnancy status in women.

|  | **Women**  **(N = 214,305)** | | | **Men**  **(N = 154,068)** | | |
| --- | --- | --- | --- | --- | --- | --- |
| Characteristic | **Reported HIV high-risk behaviour, n (%)** | **Crude prevalence ratio (95% CI)** | **Adjusted prevalence ratio**  **(95% CI)** | **Reported HIV high-risk behaviour, n (%)** | **Crude prevalence ratio**  **(95% CI)** | **Adjusted prevalence ratio**  **(95% CI)** |
| **HIV/ART/viremia status** |  |  |  |  |  |  |
| On ART undetectable | 268 (2.8) | Ref | Ref | 153 (4.3) | Ref | Ref |
| HIV negative | 4311 (2.2) | 0.80 (0.71, 0.91) *** | 0.60 (0.53, 0.69) *** | 12578 (8.7) | 1.98 (1.69, 2.32) *** | 1.06 (0.91, 1.25) |
| On ART low-level viremia | 42 (3.1) | 1.12 (0.81, 1.55) | 0.98 (0.72, 1.34) | 49 (6.5) | 1.50 (1.09, 2.07) * | 1.18 (0.87, 1.61) |
| On ART non-suppressed | 40 (2.9) | 1.01 (0.72, 1.41) | 0.76 (0.55, 1.04) | 44 (6.9) | 1.64 (1.19, 2.26) ** | 1.21 (0.88, 1.67) |
| Diagnosed but untreated | 38 (3.9) | 1.40 (1.00, 1.96) * | 1.09 (0.79, 1.51) | 53 (11.3) | 2.65 (1.97, 3.58) *** | 2.06 (1.52, 2.78) *** |
| Undiagnosed | 222 (6.1) | 2.16 (1.81, 2.58) *** | 1.28 (1.08, 1.52) ** | 260 (11.7) | 2.70 (2.22, 3.28) *** | 1.61 (1.33, 1.95) *** |
| **Age** |  |  |  |  |  |  |
| Spline 1 | - | 0.26 (0.20, 0.34) *** | 0.28 (1.08, 1.52) *** | - | 0.20 (0.18, 0.23) *** | 0.37 (0.33, 0.42) *** |
| Spline 2 | - | 0.01 (0.006, 0.02) *** | 0.28 (0.22, 0.37) *** | - | 0.14 (0.11, 0.17) *** | 0.57 (0.46, 0.71) *** |
| Spline 3 | - | 0.01 (0.003, 0.03) *** | 0.02 (0.007, 0.04) *** | - | 0.17 (0.13, 0.22) *** | 0.25 (0.19, 0.32) *** |
| **Dwelling** |  |  |  |  |  |  |
| Rural | 2498 (1.9) | Ref | Ref | 5530 (9.8) | Ref | Ref |
| Urban | 2423 (3.0) | 0.63 (0.60, 0.67) *** | 0.93 (0.87, 0.99) * | 7607 (7.9) | 0.80 (0.77, 0.83) *** | 1.03 (0.99, 1.08) |
| **Wealth quintile** |  |  |  |  |  |  |
| Lowest | 667 (1.5) | Ref | Ref | 1805 (6.0) | Ref | Ref |
| Second | 756 (1.8) | 1.18 (1.06, 1.31) ** | 1.06 (0.95, 1.17) ** | 2172 (7.2) | 1.22 (1.14, 1.29) *** | 1.08 (1.02, 1.15) ** |
| Middle | 976 (2.3) | 1.47 (1.33, 1.62) *** | 1.16 (1.05, 0.95) *** | 2808 (8.9) | 1.51 (1.42, 1.60) *** | 1.23 (1.16, 1.31) *** |
| Fourth | 1177 (2.9) | 1.87 (1.70, 2.06) *** | 1.30 (1.17, 1.45) *** | 3111 (10.3) | 1.74 (1.64, 1.85) *** | 1.35 (1.27, 1.44) *** |
| Highest | 1345 (3.4) | 2.19 (2.00, 2.41) *** | 1.42 (1.27, 1.59) *** | 3241 (10.8) | 1.83 (1.72, 1.93) *** | 1.41 (1.32, 1.50) *** |
| **Level of education** |  |  |  |  |  |  |
| None | 493 (1.0) | Ref | Ref | 777 (3.6) | Ref | Ref |
| Primary | 1800 (2.3) | 2.28 (2.07, 2.52) | 1.73 (1.56, 1.93) *** | 4518 (8.3) | 2.28 (2.12, 2.47) *** | 1.95 (1.81 2.11) *** |
| Secondary | 1976 (3.2) | 3.18 (2.88, 3.51) | 1.65 (1.48, 1.84) *** | 5563 (10.6) | 2.92 (2.71, 3.15) *** | 1.89 (1.75, 2.04) *** |
| More than secondary | 652 (3.3) | 3.28 (2.92, 3.69) | 1.65 91.46, 1.88) *** | 2279 (9.5) | 2.63 (2.42, 2.85) *** | 1.71 (1.57, 1.86) *** |
| **Marital status** |  |  |  |  |  |  |
| Currently married | 1531 (1.1) | Ref | Ref | 5488 (5.4) | Ref | Ref |
| Never married | 2121 (6.5) | 5.96 (4.49, 6.37) *** | 4.77 (4.42, 5.15) *** | 6324 (15.3) | 2.81 (2.71, 2.91) *** | 2.22 (2.12, 2.32) *** |
| Divorced/separated | 1032 (6.2) | 5.70 (5.27, 6.16) *** | 5.92 (5.45, 6.44) *** | 1196 (16.7) | 3.07 (2.90, 3.26) *** | 2.89 (2.72, 3.07) *** |
| Widower/widow | 237 (1.3) | 1.19 (1.03, 1.36) * | 2.54 (2.19, 2.95) *** | 129 (6.9) | 1.28 (1.08, 1.52) ** | 1.87 (1.58, 2.21) *** |
| **Pregnancy status** |  |  |  |  |  |  |
| Pregnant | 364 (2.2) | Ref | Ref | - | - | - |
| Not pregnant | 4557 (2.4) | 1.07 (0.96, 1.19) | 0.92 (0.83, 1.02) | - | - | - |
| **Country** |  |  |  |  |  |  |
| Côte d’Ivoire (2017-2018) | 202 (2.5) | Ref | Ref | 521 (6.9) | Ref | Ref |
| Cameroon (2017-2018) | 296 (2.5) | 1.00 (0.84, 1.21) | 0.88 (0.74, 1.06) *** | 857 (8.9) | 1.32 (1.18, 1.47) *** | 1.18 (1.06, 1.32) ** |
| Eswatini (2016-2017) | 59 (1.1) | 0.45 (0.34, 0.61) *** | 0.31 (0.23, 0.41) *** | 66 (2.0) | 0.30 (0.23, 0.38) *** | 0.24 (0.18, 0.31) *** |
| Ethiopia (2017-2018) | 70 (0.9) | 0.36 (0.28, 0.48) *** | 0.32 (0.24, 0.42) *** | 134 (2.7) | 0.40 (0.33, 0.49) *** | 0.38 (0.31, 0.46) *** |
| Kenya (2018-2019) | 163 (1.3) | 0.54 (0.44, 0.66) *** | 0.57 (0.47, 0.71) *** | 461 (5.9) | 0.88 (0.78, 0.99) * | 0.86 (0.76, 0.98) * |
| Lesotho (2016-2017) | 147 (2.5) | 1.01 (0.81, 1.25) | 0.70 (0.57, 0.87) ** | 274 (6.9) | 1.05 (0.91, 1.21) | 0.78 (0.67, 0.90) *** |
| Malawi (2015-2016) | 97 (1.1) | 0.45 (0.35, 0.58) *** | 0.36 (0.28, 0.46) *** | 323 (5.3) | 0.80 (0.70, 0.92) ** | 0.66 (0.57, 0.75) *** |
| Namibia (2017) | 107 (1.4) | 0.59 (0.46, 0.74) *** | 0.33 (0.26, 0.43) *** | 282 (5.1) | 0.78 (0.67, 0.90) *** | 0.58 (0.50, 0.67) *** |
| Nigeria (2018) | 1898 (2.3) | 0.94 (0.81, 1.09) | 1.19 (1.03, 1.38) * | 5494 (9.5) | 1.39 (1.27, 1.53) *** | 1.50 (1.37, 1.65) *** |
| Rwanda (2018-2019) | 433 (3.4) | 1.37 (1.16, 1.62) *** | 1.17 (0.99, 1.38) | 900 (8.7) | 1.28 (1.15, 1.43) *** | 1.09 (0.98, 1.21) |
| Tanzania (2016-2017) | 812 (5.3) | 2.13 (1.82, 2.49) *** | 2.05 (1.75, 2.39) *** | 1765 (15.2) | 2.24 (2.03, 2.47) *** | 2.15 (1.95, 2.37) *** |
| Uganda (2016-2017) | 344 (2.4) | 0.71 (0.58, 0.88) | 0.79 (0.66, 0.95) * | 1184 (11.4) | 1.71 (1.54, 1.90) *** | 1.45 (1.31, 1.61) *** |
| Zambia (2016) | 159 (1.8) | 0.71 (0.58, 0.88) ** | 0.52 (0.42, 0.63) *** | 510 (8.0) | 1.18 (1.05, 1.34) ** | 0.95 (0.84, 1.07) |
| Zimbabwe (2015-2016) | 134 (1.2) | 0.49 (0.40, 0.62) *** | 0.50 (0.40, 0.62) *** | 366 (5.1) | 0.76 (0.66, 0.87) *** | 0.74 (0.65, 0.85) *** |

***p < 0.001, **p < 0.01, *p < 0.05.
