# Supplementary material for: HIV risk behaviour, viraemia, and transmission across HIV cascade stages including low-level viremia: Analysis of 14 cross-sectional population-based HIV Impact Assessment surveys in sub-Saharan Africa
Source: PLOS Glob Public Health. 2024 Apr 4;4(4):e0003030. doi: 10.1371/journal.pgph.0003030 (PMC10994324; doi:10.1371/journal.pgph.0003030)
Supplement: S3 Table — (DOCX) [file pgph.0003030.s003.docx]

**S3 Table. Crude and adjusted prevalence ratios of self-reporting condomless casual partnership by sex.** Models were adjusted for age, level of education, wealth quintile, marital status, urban/rural dwelling or urbanicity size and pregnancy status in women.

|  |  | **Women**  **(N = 214,305)** |  |  | **Men**  **(N = 154,068)** |  |
| --- | --- | --- | --- | --- | --- | --- |
| Characteristic | **Reported condomless casual partnership, n (%)** | **Crude prevalence ratio (95% CI)** | **Adjusted prevalence ratio**  **(95% CI)** | **Reported condomless casual partnership, n (%)** | **Crude prevalence ratio**  **(95% CI)** | **Adjusted prevalence ratio**  **(95% CI)** |
| **HIV/ART/viremia status** |  |  |  |  |  |  |
| On ART undetectable | 331 (3.4) | Ref | Ref | 152 (4.2) | Ref | Ref |
| HIV negative | 9270 (4.8) | 1.38 (1.23, 1.54) *** | 0.99 (0.89, 1.10) | 15962 (11.0) | 2.50 (2.13, 2.93) *** | 1.04 (0.88, 1.21) |
| On ART low-level viremia | 60 (4.5) | 1.31 (0.99, 1.73) | 1.06 (0.82, 1.36) | 49 (6.5) | 1.45 (1.04, 2.03) * | 1.16 (0.85, 1.57) |
| On ART non-suppressed | 64 (4.6) | 1.33 (1.02, 1.75) * | 0.94 (0.73, 1.20) | 44 (6.9) | 1.66 (1.19, 2.30 ** | 1.19 (0.86, 1.64) |
| Diagnosed but untreated | 67 (6.8) | 2.04 (1.57, 2.64) *** | 1.79 (1.41, 2.26) *** | 43 (9.2) | 2.13 (1.53, 2.97) *** | 1.80 (1.35, 2.40) *** |
| Undiagnosed | 326 (8.9) | 2.56 (2.19, 2.92) *** | 1.42 (1.23, 1.63) *** | 263 (11.9) | 2.72 (2.23, 3.31) *** | 1.43 (1.19, 1.73) *** |
| **Age** |  |  |  |  |  |  |
| Spline 1 | - | 0.59 (0.48, 0.73) *** | 0.77 (0.64, 0.93) ** | - | 0.16 (0.14, 0.18) *** | 0.49 (0.44, 0.55) *** |
| Spline 2 | - | 0.001 (0.0008, 0.002) *** | 0.01 (0.009, 0.02) *** | - | 0.04 (0.03, 0.05) *** | 0.35 (0.29, 0.43) *** |
| Spline 3 | - | 0.003 (0.001, 0.007) *** | 0.003 (0.001, 0.006) *** | - | 0.17 (0.13, 0.21) *** | 0.25 (0.19, 0.32) *** |
| **Dwelling** |  |  |  |  |  |  |
| Rural | 4520 (5.7) | Ref | Ref | 6796 (12.1) | Ref | Ref |
| Urban | 5598 (4.3) | 0.75 (0.72, 0.78) *** | 1.13 (1.09, 1.18) *** | 9717 (10.1) | 0.83 (0.80, 0.85) *** | 1.08 (1.05, 1.12) *** |
| **Wealth quintile** |  |  |  |  |  |  |
| Lowest | 1273 (2.9) | Ref | Ref | 2236 (7.4) | Ref | Ref |
| Second | 1651 (3.9) | 1.35 (1.25, 1.45) *** | 1.13 (1.05, 1.20) *** | 2687 (8.9) | 1.21 (1.15, 1.28) *** | 1.03 (0.98, 1.09) |
| Middle | 2178 (5.0) | 1.71 (1.60, 1.84) *** | 1.19 (1.11, 1.27) *** | 3616 (11.4) | 1.58 (1.50, 1.66) *** | 1.17 (1.11, 1.22) *** |
| Fourth | 2407 (5.9) | 1.99 (1.86, 2.13) *** | 1.23 (1.14, 1.31) *** | 3876 (12.8) | 1.77 (1.68, 1.86) *** | 1.21 (1.15, 1.28) *** |
| Highest | 2609 (6.6) | 2.22 (2.07, 2.37) *** | 1.26 (1.17, 1.36) *** | 4098 (13.7) | 1.88 (1.78, 1.98) *** | 1.27 (1.20, 1.34) *** |
| **Level of education** |  |  |  |  |  |  |
| None | 750 (1.5) | Ref | Ref | 906 (4.2) | Ref | Ref |
| Primary | 3425 (4.4) | 2.80 (2.57, 3.03) *** | 1.90 (1.75, 2.05) *** | 5475 (10.1) | 2.36 (2.19, 2.53) *** | 1.83 (1.70, 1.96) *** |
| Secondary | 4547 (7.3) | 4.80 (4.43, 5.20) *** | 2.10 (1.94, 2.27) *** | 7281 (13.9) | 3.30 (3.07, 3.54) *** | 1.68 (1.57, 1.80) *** |
| More than secondary | 1396 (7.1) | 4.62 (4.21, 5.06) *** | 2.00 (1.83, 2.20) *** | 2851 (11.9) | 2.82 (2.62, 3.04) *** | 1.51 (1.40, 1.63) *** |
| **Marital status** |  |  |  |  |  |  |
| Currently married | 1255 (0.9) | Ref | Ref | 4973 (4.9) | Ref | Ref |
| Never married | 5994 (18.2) | 20.86 (19.62, 22.17) *** | 17.62 (16.50, 18.81) *** | 9961 (24.1) | 4.95 (4.79, 5.12) *** | 4.01 (3.85, 4.19) *** |
| Divorced/separated | 1879 (11.3) | 12.76 (11.89, 13.70) *** | 14.08 (13.10, 15.14) *** | 1333 (18.6) | 3.82 (3.61, 4.05) *** | 3.87 (3.66, 4.09) *** |
| Widower/widow | 990 (5.4) | 6.06 (5.57, 6.59) *** | 12.94 (11.82, 14.17) *** | 246 (13.3) | 2.73 (2.42, 3.08) *** | 3.79 (3.37, 4.27) *** |
| **Pregnancy status** |  |  |  |  |  |  |
| Pregnant | 655 (3.9) | Ref | Ref | - | - | - |
| Not pregnant | 9463 (4.9) | 1.21 (1.12, 1.31) *** | 0.76 (0.72, 0.82) *** | - | - | - |
| **Country** |  |  |  |  |  |  |
| Côte d’Ivoire (2017-2018) | 197 (2.5) | Ref | Ref | 424 (5.6) | Ref | Ref |
| Cameroon (2017-2018) | 641 (5.4) | 2.24 (1.90, 2.64) *** | 2.00 (1.71, 2.35) *** | 1011 (10.5) | 1.92 (1.71, 2.16) *** | 1.77 (1.57, 1.99) *** |
| Eswatini (2016-2017) | 29 (0.5) | 0.23 (0.16, 0.34) *** | 0.15 (0.10, 0.22) *** | 35 (1.1) | 0.19 (0.14, 0.28) *** | 0.15 (0.11, 0.21) *** |
| Ethiopia (2017-2018) | 134 (1.7) | 0.72 (0.58, 0.90) ** | 0.79 (0.64, 0.98) * | 161 (3.3) | 0.60 (0.50, 0.72) *** | 0.64 (0.54, 0.77) *** |
| Kenya (2018-2019) | 557 (4.6) | 1.91 (1.62, 2.25) *** | 2.37 (2.02, 2.79) *** | 641 (8.2) | 1.52 (1.34, 1.72) *** | 1.63 (1.44, 1.84) *** |
| Lesotho (2016-2017) | 194 (3.3) | 1.40 (1.15, 1.71) *** | 1.02 (0.84, 1.24) | 250 (6.3) | 1.19 (1.02, 1.40) * | 0.83 (0.71, 0.97) * |
| Malawi (2015-2016) | 47 (0.5) | 0.23 (0.16, 0.31) *** | 0.23 (0.17, 0.31) *** | 186 (3.1) | 0.58 (0.49, 0.69) *** | 0.49 (0.41, 0.58) *** |
| Namibia (2017) | 95 (1.3) | 0.54 (0.42, 0.69) *** | 0.27 (0.21, 0.34) *** | 176 (3.2) | 0.59 (0.50, 0.71) *** | 0.41 (0.35, 0.49) *** |
| Nigeria (2018) | 4196 (5.2) | 2.14 (1.85, 2.48) *** | 2.98 (2.58, 3.44) *** | 7542 (13.1) | 2.36 (2.13, 2.61) *** | 2.79 (2.52, 3.09) *** |
| Rwanda (2018-2019) | 1179 (9.2) | 3.79 (3.25, 4.42) *** | 3.38 (2.91, 3.39) *** | 1404 (13.6) | 2.47 (2.21, 2.76) *** | 2.15 (1.93, 2.40) *** |
| Tanzania (2016-2017) | 1528 (10.0) | 4.12 (3.54, 4.79) *** | 4.49 (3.87, 5.20) *** | 2238 (19.3) | 3.49 (3.14, 3.89) *** | 3.48 (3.13, 3.87) *** |
| Uganda (2016-2017) | 796 (5.6) | 2.29 (1.95, 2.68) *** | 2.16 (1.85, 2.53) *** | 1415 (13.6) | 2.50 (2.23, 2.79) *** | 2.20 (1.97, 2.46) *** |
| Zambia (2016) | 400 (4.5) | 1.83 (1.54, 2.18) *** | 1.50 (1.27, 1.78) *** | 752 (11.7) | 2.15 (1.91, 2.43) *** | 1.78 (1.58, 2.01) *** |
| Zimbabwe (2015-2016) | 125 (1.1) | 0.48 (0.38, 0.60) *** | 0.57 (0.46, 0.71) *** | 278 (3.9) | 0.72 (0.62, 0.84) *** | 0.72 (0.62, 0.84) *** |

***p < 0.001, **p < 0.01, *p < 0.05.
