# Supplementary material for: HIV risk behaviour, viraemia, and transmission across HIV cascade stages including low-level viremia: Analysis of 14 cross-sectional population-based HIV Impact Assessment surveys in sub-Saharan Africa
Source: PLOS Glob Public Health. 2024 Apr 4;4(4):e0003030. doi: 10.1371/journal.pgph.0003030 (PMC10994324; doi:10.1371/journal.pgph.0003030)
Supplement: S4 Table — (DOCX) [file pgph.0003030.s004.docx]

**S4 Table. Crude and adjusted prevalence ratios of self-reporting transactional partnership by sex.** Models were adjusted for age, level of education, wealth quintile, marital status, urban/rural dwelling or urbanicity size and pregnancy status in women.

|  |  | **Women**  **(N = 214,305)** |  |  | **Men**  **(N = 154,068)** |  |
| --- | --- | --- | --- | --- | --- | --- |
| Characteristic | **Reported**  **transactional partnership, n (%)** | **Crude prevalence ratio (95% CI)** | **Adjusted prevalence ratio**  **(95% CI)** | **Reported transactional partnership, n (%)** | **Crude prevalence ratio**  **(95% CI)** | **Adjusted prevalence ratio**  **(95% CI)** |
| **HIV/ART/viremia status** |  |  |  |  |  |  |
| On ART undetectable | 945 (9.8) | Ref | Ref | 97 (2.7) | Ref | Ref |
| HIV negative | 16308 (8.5) | 0.86 (0.81, 0.92) *** | 0.68 (0.64, 0.73) *** | 5457 (3.8) | 1.40 (1.15, 1.70) *** | 0.86 (0.70, 1.05) |
| On ART low-level viremia | 128 (9.6) | 0.97 (0.81, 1.16) | 0.88 (0.74, 1.05) | 16 (2.2) | 0.78 (0.46, 1.32) | 0.67 (0.40, 1.14) |
| On ART non-suppressed | 137 (9.8) | 1.00 (0.84, 1.19) | 0.84 (0.71, 1.00) * | 23 (3.6) | 1.33 (0.84, 2.09) | 1.12 (0.71, 1.76) |
| Diagnosed but untreated | 117 (12.0) | 1.22 (1.01, 1.47) * | 1.05 (0.88, 1.25) | 12 (2.6) | 0.95 (0.52, 1.72) | 0.84 (0.47, 1.53) |
| Undiagnosed | 436 (12.0) | 1.24 (1.11, 1.38) *** | 0.93 (0.83, 1.03) | 95 (4.3) | 1.59 (1.20, 2.09) ** | 1.22 (0.93, 1.61) |
| **Age** |  |  |  |  |  |  |
| Spline 1 | - | 0.75 (0.67, 0.84) *** | 0.68 (0.61, 0.76) *** | - | 0.73 (0.63, 0.85) *** | 0.66 (0.56, 0.78) *** |
| Spline 2 | - | 0.02 (0.01, 0.02) *** | 0.04 (0.03, 0.05) *** | - | 0.22 (0.16, 0.29) *** | 0.28 (0.20, 0.40) *** |
| Spline 3 | - | 0.01 (0.007, 0.02) *** | 0.02 (0.01, 0.03) *** | - | 0.25 (0.18, 0.35) *** | 0.31 (0.22, 0.45) *** |
| **Dwelling** |  |  |  |  |  |  |
| Rural | 6142 (7.8) | Ref | Ref | 1809 (3.2) | Ref | Ref |
| Urban | 11929 (9.2) | 1.18 (1.15, 1.22) *** | 1.10 (1.06, 1.14) *** | 3891 (4.1) | 1.26 (1.19, 1.33) *** | 1.13 (1.06, 1.21) *** |
| **Wealth quintile** |  |  |  |  |  |  |
| Lowest | 4207 (9.7) | Ref | Ref | 1454 (4.8) | Ref | Ref |
| Second | 3826 (9.2) | 0.93 (0.89, 0.98) ** | 0.95 (0.91, 0.99) * | 1260 (4.2) | 0.87 (0.80, 0.94) *** | 0.93 (0.86, 1.01) |
| Middle | 3812 (8.8) | 0.90 (0.86, 0.94) *** | 0.91 (0.87, 0.95) *** | 1095 (3.5) | 0.72 (0.66, 0.78) *** | 0.80 (0.74, 0.87) *** |
| Fourth | 3368 (8.2) | 0.84 (0.80, 0.87) *** | 0.85 (0.80, 0.89) *** | 1025 (3.4) | 0.70 (0.64, 0.76) *** | 0.80 (0.73, 0.87) *** |
| Highest | 2858 (7.2) | 0.73 (0.70, 0.77) *** | 0.76 (0.72, 0.81) *** | 866 (2.9) | 0.60 (0.55, 0.65) *** | 0.71 (0.64, 0.79) *** |
| **Level of education** |  |  |  |  |  |  |
| None | 3716 (7.5) | Ref | Ref | 1164 (5.4) | Ref | Ref |
| Primary | 7026 (9.0) | 1.21 (1.16, 1.26) *** | 1.16 (1.11, 1.20) *** | 1714 (3.2) | 0.59 (0.55, 0.63) *** | 0.68 (0.63, 0.74) *** |
| Secondary | 6077 (9.9) | 1.35 (1.30, 1.41) *** | 1.06 (1.02, 1.11) ** | 1994 (3.8) | 0.71 (0.66, 0.76) *** | 0.69 (0.64, 0.74) *** |
| More than secondary | 1252 (6.4) | 0.90 (0.84, 0.96) *** | 0.82 (0.77, 0.88) *** | 828 (3.5) | 0.65 (0.59, 0.70) *** | 0.68 (0.62, 0.75) *** |
| **Marital status** |  |  |  |  |  |  |
| Currently married | 10527 (7.4) | Ref | Ref | 3585 (3.5) | Ref | Ref |
| Never married | 4380 (13.4) | 1.86 (1.80, 1.92) *** | 1.77 (1.70, 1.84) *** | 1825 (4.5) | 1.27 (1.20, 1.34) *** | 1.20 (1.11, 1.30) *** |
| Divorced/separated | 2185 (13.2) | 1.76 (1.68, 1.84) *** | 1.85 (1.77, 1.93) *** | 254 (3.6) | 1.03 (0.91, 1.17) | 1.13 (1.00, 1.28) * |
| Widower/widow | 979 (5.3) | 0.72 (0.67, 0.77) *** | 1.27 (1.18, 1.36) *** | 36 (2.0) | 0.56 (0.40, 0.77) *** | 0.73 (0.53, 1.02) |
| **Pregnancy status** |  |  |  |  |  |  |
| Pregnant | 1645 (9.9) | Ref | Ref | - | - | - |
| Not pregnant | 16426 (8.5) | 0.86 (0.82, 0.90) *** | 0.96 (0.92, 1.01) | - | - | - |
| **Country** |  |  |  |  |  |  |
| Côte d’Ivoire (2017-2018) | 306 (3.9) | Ref | Ref | 204 (2.7) | Ref | Ref |
| Cameroon (2017-2018) | 627 (5.3) | 1.43 (1.24, 1.65) *** | 1.43 (1.24, 1.65) *** | 308 (3.2) | 1.21 (1.01, 1.45) * | 1.27 (1.05, 1.52) * |
| Eswatini (2016-2017) | 182 (3.5) | 0.92 (0.76, 1.11) | 0.90 (0.75, 1.09) | 65 (2.0) | 0.73 (0.55, 0.97) * | 0.79 (0.59, 1.05) |
| Ethiopia (2017-2018) | 233 (3.0) | 0.82 (0.69, 0.97) * | 0.84 (0.71, 1.00) * | 94 (1.9) | 0.71 (0.55, 0.92) ** | 0.81 (0.63, 1.05) |
| Kenya (2018-2019) | 905 (7.5) | 2.02 (1.77, 2.31) *** | 2.16 (1.89, 2.47) *** | 270 (3.5) | 1.30 (1.08, 1.56) ** | 1.48 (1.22, 1.78) *** |
| Lesotho (2016-2017) | 277 4.7) | 1.29 (1.09, 1.52) ** | 1.12 (0.95, 1.33) | 86 (2.2) | 0.81 (0.63, 1.04) | 0.80 (0.62, 1.04) |
| Malawi (2015-2016) | 944 (11.0) | 2.98 (2.61, 3.41) *** | 2.83 (2.48, 3.24) *** | 175 (2.9) | 1.10 (0.90, 1.35) | 1.22 (0.99, 1.50) |
| Namibia (2017) | 542 (7.3) | 1.99 (1.72, 2.30) *** | 1.57 (1.35, 1.81) *** | 203 (3.7) | 1.37 (1.12, 1.66) ** | 1.33 (1.08, 1.62) ** |
| Nigeria (2018) | 7988 (10.0) | 2.69 (2.38, 3.03) *** | 3.09 (2.74, 3.49) *** | 2882 (5.0) | 1.88 (1.62, 2.18) *** | 2.07 (1.78, 2.41) *** |
| Rwanda (2018-2019) | 674 (5.3) | 1.38 (1.20, 1.59) *** | 1.44 (1.25, 1.66) *** | 172 (1.7) | 0.62 (0.51, 0.76) *** | 0.69 (0.56, 0.85) *** |
| Tanzania (2016-2017) | 1164 (7.6) | 2.03 (1.78, 2.32) *** | 2.16 (1.90, 2.46) *** | 226 (2.0) | 0.72 (0.59, 0.87) *** | 0.79 (0.65, 0.96) * |
| Uganda (2016-2017) | 2302 (16.2) | 4.37 (3.86, 4.95) *** | 4.03 (3.56, 4.57) *** | 633 (6.1) | 2.27 (1.93, 2.67) *** | 2.37 (2.00, 2.80) *** |
| Zambia (2016) | 1142 (12.8) | 3.49 (3.06, 3.97) *** | 3.13 (2.75, 3.56) *** | 222 (3.5) | 1.30 (1.07, 1.58) ** | 1.38 (1.13, 1.69) ** |
| Zimbabwe (2015-2016) | 785 (7.2) | 1.97 (1.72, 2.26) *** | 2.16 (1.88, 2.48) *** | 160 (2.3) | 0.84 (0.68, 1.04) | 0.96 (0.77, 1.19) |

***p < 0.001, **p < 0.01, *p < 0.05.
