# Supplementary material for: HIV risk behaviour, viraemia, and transmission across HIV cascade stages including low-level viremia: Analysis of 14 cross-sectional population-based HIV Impact Assessment surveys in sub-Saharan Africa
Source: PLOS Glob Public Health. 2024 Apr 4;4(4):e0003030. doi: 10.1371/journal.pgph.0003030 (PMC10994324; doi:10.1371/journal.pgph.0003030)
Supplement: S5 Table — (DOCX) [file pgph.0003030.s005.docx]

**S5 Table. Crude and adjusted prevalence ratios of self-reporting condomless last sex (with any partner) by sex.** Models were adjusted for age, level of education, wealth quintile, marital status, urban/rural dwelling or urbanicity size and pregnancy status in women.

|  |  | **Women**  **(N = 208,556)** |  |  | **Men**  **(N = 148,010)** |  |
| --- | --- | --- | --- | --- | --- | --- |
| Characteristic | **Reported**  **condomless last sex (with any partner, n (%)** | **Crude prevalence ratio (95% CI)** | **Adjusted prevalence ratio**  **(95% CI)** | **Reported condomless last sex (with any partner, n (%)** | **Crude prevalence ratio**  **(95% CI)** | **Adjusted prevalence ratio**  **(95% CI)** |
| **HIV/ART/viremia status** |  |  |  |  |  |  |
| On ART undetectable | 3375 (35.3) | Ref | Ref | 1323 (37.4) | Ref | Ref |
| HIV negative | 134657 (70.2) | 1.93 (1.88, 1.98)*** | 1.44 (1.41, 1.48)*** | 93523 (66.5) | 1.74 (1.67, 1.81)*** | 1.71 (1.64, 1.78)*** |
| On ART low-level viremia | 500 (37.5) | 1.06 (0.99, 1.14) | 1.06 (0.99, 1.13) | 321 (44.0) | 1.17 (1.07, 1.28)*** | 1.17 (1.07, 1.27)*** |
| On ART non-suppressed | 578 (41.6) | 1.17 (1.10, 1.25)*** | 1.09 (1.02, 1.15)** | 276 (44.9) | 1.20 (1.09, 1.32)*** | 1.20 (1.10, 1.31)*** |
| Diagnosed but untreated | 475 (49.0) | 1.38 (1.29, 1.47)*** | 1.28 (1.20, 1.36)*** | 243 (53.5) | 1.40 (1.27, 1.54)*** | 1.40 (1.29, 1.53)*** |
| Undiagnosed | 2175 (60.1) | 1.67 (1.61, 1.73)*** | 1.42 (1.38, 1.47)*** | 1376 (64.8) | 1.69 (1.61, 1.78)*** | 1.67 (1.59, 1.75)*** |
| **Age** |  |  |  |  |  |  |
| Spline 1 | - | 0.86 (0.84, 0.88)*** | 0.97 (0.96, 0.99)*** | - | 1.59 (1.56, 1.62)*** | 1.07 (1.05, 1.08)*** |
| Spline 2 | - | 0.44 (0.42, 0.47)*** | 0.56 (0.54, 0.57)*** | - | 4.17 (3.95, 4.40)*** | 0.99 (0.95, 1.04) |
| Spline 3 | - | 0.15 (0.14, 0.16)*** | 0.37 (0.36, 0.39)*** | - | 1.12 (1.08, 1.17)*** | 0.82 (0.79, 0.84)*** |
| **Dwelling** |  |  |  |  |  |  |
| Rural | 90301 (69.5) | Ref | Ref | 63686 (67.8) | Ref | Ref |
| Urban | 51459 (65.4) | 1.06 (1.05, 1.07)*** | 1.00 (0.99, 1.01) | 33376 (61.7) | 1.10 (1.09, 1.11)*** | 1.01 (1.00, 1.02)** |
| **Wealth quintile** |  |  |  |  |  |  |
| Lowest | 31420 (72.3) | Ref | Ref | 21316 (72.2) | Ref | Ref |
| Second | 29008 (69.5) | 0.96 (0.95, 0.97)*** | 0.99 (0.98, 1.00)** | 19768 (67.3) | 0.93 (0.92, 0.94)*** | 0.98 (0.97, 0.99)*** |
| Middle | 28625 (66.3) | 0.92 (0.91, 0.93)*** | 0.97 (0.96, 0.98)*** | 19816 (64.5) | 0.90 (0.88, 0.91)*** | 0.97 (0.96, 0.98)*** |
| Fourth | 27020 (66.2) | 0.92 (0.91, 0.93)*** | 0.97 (0.96, 0.98)*** | 18736 (63.7) | 0.88 (0.87, 0.89)*** | 0.98 (0.97, 0.99)*** |
| Highest | 25687 (65.2) | 0.91 (0.90, 0.92)*** | 0.97 (0.96, 0.98)*** | 17426 (60.2) | 0.84 (0.83, 0.85)*** | 0.96 (0.95, 0.97)*** |
| **Level of education** |  |  |  |  |  |  |
| None | 35969 (72.3) | Ref | Ref | 16177 (75.6) | Ref | Ref |
| Primary | 52599 (67.6) | 0.95 (0.94, 0.96)*** | 0.98 (0.97, 0.99)*** | 36642 (69.8) | 0.93 (0.92, 0.94)*** | 0.98 (0.97, 0.99)*** |
| Secondary | 40715 (66.3) | 0.94 (0.93, 0.95)*** | 0.97 (0.96, 0.97)*** | 30239 (59.4) | 0.79 (0.78, 0.80)*** | 0.96 (0.95, 0.97) *** |
| More than secondary | 12477 (63.6) | 0.91 (0.90, 0.92)*** | 0.96 (0.95, 0.97)*** | 14004 (60.3) | 0.81 (0.80, 0.82)*** | 0.95 (0.94, 0.96)*** |
| **Marital status** |  |  |  |  |  |  |
| Currently married | 117937 (83.2) | Ref | Ref | 79557 (79.6) | Ref | Ref |
| Never married | 14590 (45.3) | 0.55 (0.54, 0.56)*** | 0.58 (0.57, 0.59)*** | 14157 (35.8) | 0.45 (0.44, 0.46) | 0.46 (0.45, 0.47)*** |
| Divorced/separated | 6591 (40.3) | 0.49 (0.48, 0.50)*** | 0.54 (0.53, 0.55)*** | 2885 (42.7) | 0.53, 0.52, 0.55) | 0.54 (0.52, 0.55)*** |
| Widower/widow | 2642 (14.4) | 0.17 (0.16, 1.18)*** | 0.27 (0.26, 0.28)*** | 463 (25.2) | 0.32 (0.29, 0.34) | 0.35 (0.33, 0.38)*** |
| **Pregnancy status** |  |  |  |  |  |  |
| Pregnant | 15329 (92.5) | Ref | Ref | - | - | - |
| Not pregnant | 126431 (65.9) | 0.72 (0.71, 0.73)*** | 0.92 (0.91, 0.92)*** | - | - | - |
| **Country** |  |  |  |  |  |  |
| Côte d’Ivoire (2017-2018) | 5553 (72.3) | Ref | Ref | 4586 (67.1) | Ref | Ref |
| Cameroon (2017-2018) | 8444 (72.0) | 0.99 (0.97, 1.01) | 1.03 (1.02, 1.05)*** | 6011 (68.5) | 1.02 (0.99, 1.04) | 1.07 (1.05, 1.09)*** |
| Eswatini (2016-2017) | 1823 (34.6) | 0.47 (0.45, 0.49)*** | 0.71 (0.69, 0.74)*** | 1199 (37.0) | 0.55 (0.52, 0.58)*** | 0.72 (0.69, 0.75)*** |
| Ethiopia (2017-2018) | 4842 (62.7) | 0.86 (0.85, 0.88)*** | 0.93 (0.92, 0.95)*** | 3261 (67.7) | 1.00 (0.98, 1.03) | 0.99 (0.97, 1.01) |
| Kenya (2018-2019) | 8602 (71.6) | 0.98 (0.97, 1.00) | 1.04 (1.03, 1.05)*** | 5388 (72.5) | 1.07 (1.05, 1.10)*** | 1.07 (1.05, 1.09)*** |
| Lesotho (2016-2017) | 2740 (46.4) | 0.64 (0.62, 0.66)*** | 0.79 (0.77, 0.81)*** | 1500 (38.0) | 0.57 (0.54, 0.59)*** | 0.70 (0.67, 0.72)*** |
| Malawi (2015-2016) | 5944 (68.8) | 0.95 (0.93, 0.97)*** | 1.01 (0.99, 1.03) | 3969 (65.3) | 0.97 (0.95, 1.00)* | 1.01 (0.99, 1.03) |
| Namibia (2017) | 3488 (47.8) | 0.66 (0.64, 0.68)*** | 0.87 (0.85, 0.89)*** | 2280 (44.8) | 0.67 (0.64, 0.69)*** | 0.85 (0.83, 0.87)*** |
| Nigeria (2018) | 59254 (73.4) | 1.01 (0.99, 1.02) | 1.02 (1.00, 1.03)* | 39483 (68.4) | 1.02 (1.00, 1.03)* | 0.94 (0.93, 0.96)*** |
| Rwanda (2018-2019) | 8411 (67.3) | 0.93 (0.92, 0.95)*** | 1.03 (1.02, 1.05)*** | 6585 (68.3) | 1.02 (1.00, 1.05)* | 1.06 (1.04, 1.08)*** |
| Tanzania (2016-2017) | 9817 (65.7) | 0.90 (0.89, 0.92)*** | 0.96 (0.94, 0.97)*** | 7240 (68.4) | 1.01 (0.99, 1.03) | 0.95 (0.94, 0.97)*** |
| Uganda (2016-2017) | 10234 (71.8) | 0.99 (0.98, 1.01) | 1.03 (1.02, 1.05)*** | 7435 (72.1) | 1.08 (1.06, 1.10)*** | 1.08 (1.06, 1.10)*** |
| Zambia (2016) | 6053 (67.6) | 0.94 (0.92, 0.96)*** | 1.00 (0.98, 1.01) | 3926 (61.3) | 0.91 (0.89, 0.94)*** | 0.95 (0.93, 0.97)*** |
| Zimbabwe (2015-2016) | 6555 (60.3) | 0.83 (0.81, 0.85)*** | 0.99 (0.98, 1.01) | 4199 (58.8) | 0.87 (0.85, 0.89)*** | 0.93 (0.91, 0.95)*** |

***p < 0.001, **p < 0.01, *p < 0.05.
