# Supplementary material for: HIV risk behaviour, viraemia, and transmission across HIV cascade stages including low-level viremia: Analysis of 14 cross-sectional population-based HIV Impact Assessment surveys in sub-Saharan Africa
Source: PLOS Glob Public Health. 2024 Apr 4;4(4):e0003030. doi: 10.1371/journal.pgph.0003030 (PMC10994324; doi:10.1371/journal.pgph.0003030)
Supplement: S6 Table — (DOCX) [file pgph.0003030.s006.docx]

**S6 Table. Crude and adjusted prevalence ratios of self-reporting both multiple sexual partnership and condomless last sex (with any partner) by sex.** Models were adjusted for age, level of education, wealth quintile, marital status, urban/rural dwelling or urbanicity size and pregnancy status in women.

|  |  | **Women**  **(N = 214,305)** |  |  | **Men**  **(N = 154,068)** |  |
| --- | --- | --- | --- | --- | --- | --- |
| Characteristic | **Reported**  **multiple sexual partnership and condomless last sex (with any partner, n (%)** | **Crude prevalence ratio (95% CI)** | **Adjusted prevalence ratio**  **(95% CI)** | **Reported multiple sexual partnership and condomless last sex (with any partner, n (%)** | **Crude prevalence ratio**  **(95% CI)** | **Adjusted prevalence ratio**  **(95% CI)** |
| **HIV/ART/viremia status** |  |  |  |  |  |  |
| On ART undetectable | 246 (2.6) | Ref | Ref | 270 (7.6) | Ref | Ref |
| HIV negative | 4733 (2.5) | 0.96 (0.84, 1.09) | 0.85 (0.74, 0.98)* | 22576 (16.1) | 2.08 (1.85, 2.33)*** | 1.66 (1.48, 1.87)*** |
| On ART low-level viremia | 42 (3.1) | 1.20 (0.86, 1.68) | 1.11 (0.80, 1.53) | 76 (10.4) | 1.35 (1.06, 1.72)* | 1.26 (0.99, 1.59) |
| On ART non-suppressed | 47 (3.4) | 1.30 (0.95, 1.77) | 0.98 (0.72, 1.33) | 59 (9.6) | 1.25 (0.96, 1.63) | 1.09 (0.84, 1.42) |
| Diagnosed but untreated | 48 (4.9) | 1.91 (1.40, 2.60)*** | 1.49 (1.11, 2.02)** | 71 (15.6) | 2.02 (1.59, 2.57)*** | 1.91 (1.51, 2.43)*** |
| Undiagnosed | 243 (6.7) | 2.60 (2.18, 3.10)*** | 1.80 (1.51, 2.14)*** | 404 (19.0) | 2.46 (2.13, 2.84)*** | 2.04 (1.77, 2.36)*** |
| **Age** |  |  |  |  |  |  |
| Spline 1 | - | 0.31 (0.25, 0.38)*** | 0.34 (0.27, 0.42)*** | - | 1.65 (1.55, 1.76)*** | 1.63 (1.53, 1.74)*** |
| Spline 2 | - | 0.03 (0.02, 0.05)*** | 0.13 (0.08, 0.21)*** | - | 1.54 (1.33, 1.78)*** | 1.72 (1.46, 2.03)*** |
| Spline 3 | - | 0.03 (0.01, 0.05)*** | 0.04 (0.02, 0.08)*** | - | 0.61 (0.54, 0.70)*** | 0.62 (0.54, 0.70)*** |
| **Dwelling** |  |  |  |  |  |  |
| Rural | 2900 (2.2) | Ref | Ref | 15664 (16.7) | Ref | Ref |
| Urban | 2459 (3.1) | 0.72 (0.68, 0.76)*** | 0.91 (0.86, 0.98)** | 7792 (14.4) | 1.16 (1.13, 1.19)*** | 1.09 (1.06, 1.13)*** |
| **Wealth quintile** |  |  |  |  |  |  |
| Lowest | 898 (2.1) | Ref | Ref | 5220 (17.7) | Ref | Ref |
| Second | 891 (2.1) | 1.04 (0.95, 1.14) | 0.95 (0.86, 1.04) | 4860 (16.5) | 0.94 (0.90, 0.97)*** | 1.00 (0.97, 1.04) |
| Middle | 1073 (2.5) | 1.21 (1.10, 1.32)*** | 1.00 (0.91, 1.09) | 4811 (15.7) | 0.88 (0.85, 0.92)*** | 0.98 (0.95, 1.02) |
| Fourth | 1201 (2.9) | 1.42 (1.30, 1.55)*** | 1.06 (0.96, 1.17) | 4578 (15.6) | 0.88 (0.84, 0.91)*** | 1.00 (0.96, 1.04) |
| Highest | 1296 (3.3) | 1.58 (1.45, 1.72)*** | 1.11 (1.00, 1.23)* | 3987 (13.8) | 0.77 (0.74, 0.80)*** | 0.93 (0.89, 0.97)** |
| **Level of education** |  |  |  |  |  |  |
| None | 716 (1.4) | Ref | Ref | 4695 (21.9) | Ref | Ref |
| Primary | 1951 (2.5) | 1.73 (1.58, 1.88)*** | 1.55 (1.42, 1.70)*** | 8166 (15.5) | 0.71 (0.69, 0.73)*** | 0.83 (0.80, 0.86)*** |
| Secondary | 2041 (3.3) | 2.29 (2.10, 2.49)*** | 1.42 (1.29, 1.56)*** | 7411 (14.6) | 0.66 (0.64, 0.68)*** | 0.77 (0.74, 0.80)*** |
| More than secondary | 651 (3.3) | 2.29 (2.06, 2.55)*** | 1.39 (1.23, 1.56)*** | 3184 (13.7) | 0.62 (0.60, 0.65)*** | 0.70 (0.67, 0.73)*** |
| **Marital status** |  |  |  |  |  |  |
| Currently married | 2230 (1.6) | Ref | Ref | 17001 (17.0) | Ref | Ref |
| Never married | 1925 (6.0) | 3.79 (3.57, 4.03) | 3.41 (3.18, 3.67)*** | 5298 (13.4) | 0.78 (0.76, 0.81)*** | 1.07 (1.03, 1.11)*** |
| Divorced/separated | 927 (5.7) | 3.58 (3.32, 3.87) | 3.94 (3.64, 4.28)*** | 1029 (15.2) | 0.89 (0.84, 0.94)*** | 0.97 (0.91, 1.03) |
| Widower/widow | 277 (1.5) | 0.95 (0.84, 1.08) | 1.86 (1.62, 2.13)*** | 128 (7.0) | 0.41 (0.35, 0.48)*** | 0.46 (0.39, 0.55)*** |
| **Pregnancy status** |  |  |  |  |  |  |
| Pregnant | 473 (2.9) | Ref | Ref | - | - | - |
| Not pregnant | 4886 (2.6) | 0.89 (0.81, 0.97) | 0.82 (0.75, 0.90)*** | - | - | - |
| **Country** |  |  |  |  |  |  |
| Côte d’Ivoire (2017-2018) | 228 (3.0) | Ref | Ref | 1295 (18.9) | Ref | Ref |
| Cameroon (2017-2018) | 251 (2.1) | 0.74 (0.62, 0.89) | 0.68 (0.57, 0.82)*** | 1423 (16.2) | 0.86 (0.80, 0.92)*** | 0.95 (0.88, 1.02) |
| Eswatini (2016-2017) | 82 (1.6) | 0.54 (0.42, 0.69) | 0.43 (0.33, 0.56)*** | 216 (6.7) | 0.35 (0.31, 0.40)*** | 0.43 (0.37, 0.49)*** |
| Ethiopia (2017-2018) | 84 (1.1) | 0.37 (0.29, 0.48) | 0.34 (0.26, 0.44)*** | 144 (3.0) | 0.16 (0.13, 0.19)*** | 0.18 (0.15, 0.21)*** |
| Kenya (2018-2019) | 142 (1.2) | 0.40 (0.33, 0.50) | 0.43 (0.35, 0.53)*** | 614 (8.3) | 0.43 (0.40, 0.48)*** | 0.47 (0.42, 0.51)*** |
| Lesotho (2016-2017) | 309 (5.2) | 1.81 (1.53, 2.14) | 0.34 (0.26, 0.44)*** | 434 (11.0) | 0.58 (0.52, 0.64)*** | 0.67 (0.61, 0.75)*** |
| Malawi (2015-2016) | 204 (2.4) | 0.81 (0.67, 0.97) | 0.43 (0.35, 0.53)*** | 900 (14.8) | 0.78 (0.72, 0.85)*** | 0.89 (0.82, 0.96)*** |
| Namibia (2017) | 100 (1.4) | 0.47 (0.37, 0.60) | 0.31 (0.24, 0.39)*** | 231 (4.5) | 0.24 (0.21, 0.28)*** | 0.27 (0.23, 0.31)** |
| Nigeria (2018) | 2274 (2.8) | 0.96 (0.84, 1.10) | 1.18 (1.03, 1.35)* | 11501 (19.9) | 1.05 (0.99, 1.11) | 1.11 (1.06, 1.18)*** |
| Rwanda (2018-2019) | 256 (2.1) | 0.71 (0.59, 0.85) | 0.65 (0.54, 0.77)*** | 689 (7.2) | 0.38 (0.35, 0.41)*** | 0.40 (0.37, 0.44)*** |
| Tanzania (2016-2017) | 620 (4.2) | 1.42 (1.22, 1.66) | 1.44 (1.24, 1.68)*** | 1996 (18.9) | 0.99 (0.93, 1.05) | 1.07 (1.00, 1.15)* |
| Uganda (2016-2017) | 465 (3.3) | 1.12 (0.96, 1.31) | 0.96 (0.82, 1.13) | 2342 (22.7) | 1.20 (1.13, 1.28)*** | 1.35 (1.26, 1.44)*** |
| Zambia (2016) | 177 (2.0) | 0.68 (0.55, 0.82) | 0.53 (0.44, 0.65)*** | 837 (13.1) | 0.69 (0.63, 0.75)*** | 0.79 (0.72, 0.86)*** |
| Zimbabwe (2015-2016) | 167 (1.5) | 0.52 (0.43, 0.64) | 0.56 (0.46, 0.68)*** | 834 (11.7) | 0.62 (0.57, 0.67)*** | 0.73 (0.67, 0.79)*** |

***p < 0.001, **p < 0.01, *p < 0.05.
