# Supplementary material for: HIV risk behaviour, viraemia, and transmission across HIV cascade stages including low-level viremia: Analysis of 14 cross-sectional population-based HIV Impact Assessment surveys in sub-Saharan Africa
Source: PLOS Glob Public Health. 2024 Apr 4;4(4):e0003030. doi: 10.1371/journal.pgph.0003030 (PMC10994324; doi:10.1371/journal.pgph.0003030)
Supplement: S7 Table — (DOCX) [file pgph.0003030.s007.docx]

**S7 Table.** **Predicted prevalence ratios of self-reporting high-risk sex relative to PLHIV On ART undetectable (≤50 copies/mL) for each of the 14 survey countries by sex.**

|  |  | **Predicted prevalence ratio (95% confidence interval) of self-reporting HIV high-risk sex** | |
| --- | --- | --- | --- |
| **Survey** | **Subgroup (Reference group: On ART undetectable)** | **Women** | **Men** |
| Côte d’Ivoire (2017-18) | HIV negative | 1.07 (0.99, 1.14) | 0.73 (0.64, 0.83) |
|  | On ART undetectable | 1.0 (Ref) | 1.0 (Ref) |
|  | On ART low-level viremia | 1.37 (1.08, 1.75) | 1.09 (0.85, 1.42) |
|  | On ART non-suppressed | 0.80 (0.62, 1.03) | 0.88 (0.68, 1.15) |
|  | Diagnosed but untreated | 1.08 (0.83, 1.40) | 0.90 (0.71, 1.14) |
|  | Undiagnosed | 2.09 (1.93, 2.26) | 1.25 (1.13, 1.37) |
| Cameroon (2017-18) | HIV negative | 0.84 (0.78, 0.90) | 0.66 (0.58, 0.75) |
|  | On ART undetectable | 1.0 (Ref) | 1.0 (Ref) |
|  | On ART low-level viremia | 1.28 (1.00, 1.64) | 1.20 (0.92, 1.57) |
|  | On ART non-suppressed | 1.16 (0.90, 1.49) | 0.83 (0.63, 1.09) |
|  | Diagnosed but untreated | 1.26 (0.97, 1.64) | 1.44 (1.13, 1.86) |
|  | Undiagnosed | 1.90 (1.75, 2.06) | 1.37 (1.25, 1.52) |
| Eswatini (2016-17) | HIV negative | 1.17 (1.10, 1.24) | 0.76 (0.66, 0.87) |
|  | On ART undetectable | 1.0 (Ref) | 1.0 (Ref) |
|  | On ART low-level viremia | 1.28 (1.01, 1.62) | 0.88 (0.68, 1.14) |
|  | On ART non-suppressed | 1.07 (0.84, 1.37) | 1.09 (0.83, 1.44) |
|  | Diagnosed but untreated | 1.35 (1.05, 1.73) | 1.55 (1.22, 1.99) |
|  | Undiagnosed | 2.56 (2.36, 2.78) | 1.64 (1.49, 1.82) |
| Ethiopia (2017-18) | HIV negative | 0.92 (0.86, 0.99) | 0.63 (0.55, 0.71) |
|  | On ART undetectable | 1.0 (Ref) | 1.0 (Ref) |
|  | On ART low-level viremia | 1.59 (1.24, 2.05) | 0.94 (0.73, 1.22) |
|  | On ART non-suppressed | 0.97 (0.76, 1.26) | 0.88 (0.68, 1.16) |
|  | Diagnosed but untreated | 1.33 (1.02, 1.74) | 1.22 (0.96, 1.58) |
|  | Undiagnosed | 2.34 (2.16, 2.54) | 1.29 (1.17, 1.41) |
| Kenya (2018-19) | HIV negative | 0.89 (0.84, 0.95) | 0.67 (0.59, 0.76) |
|  | On ART undetectable | 1.0 (Ref) | 1.0 (Ref) |
|  | On ART low-level viremia | 1.37 (1.08, 1.75) | 0.90 (0.70, 1.17) |
|  | On ART non-suppressed | 1.10 (0.86, 1.41) | 0.82 (0.63, 1.08) |
|  | Diagnosed but untreated | 1.30 (1.01, 1.68) | 1.89 (1.48, 2.44) |
|  | Undiagnosed | 1.92 (1.77, 2.07) | 1.54 (1.40, 1.69) |
| Lesotho (2016-17) | HIV negative | 1.03 (0.97, 1.10) | 0.76 (0.67, 0.86) |
|  | On ART undetectable | 1.0 (Ref) | 1.0 (Ref) |
|  | On ART low-level viremia | 1.22 (0.96, 1.56) | 0.89 (0.69, 1.16) |
|  | On ART non-suppressed | 1.01 (0.79, 1.31) | 0.94 (0.72, 1.24) |
|  | Diagnosed but untreated | 1.25 (0.97, 1.63) | 1.50 (1.18, 1.93) |
|  | Undiagnosed | 2.10 (1.95, 2.28) | 1.45 (1.32, 1.59) |
| Malawi (2015-16) | HIV negative | 0.96 (0.89, 1.03) | 0.70 (0.61, 0.80) |
|  | On ART undetectable | 1.0 (Ref) | 1.0 (Ref) |
|  | On ART low-level viremia | 1.32 (1.03, 1.70) | 0.87 (0.68, 1.14) |
|  | On ART non-suppressed | 0.86 (0.66, 1.11) | 0.94 (0.72, 1.24) |
|  | Diagnosed but untreated | 1.45 (1.12, 1.90) | 1.38 (1.08, 1.77) |
|  | Undiagnosed | 2.06 (1.90, 2.24) | 1.38 (1.25, 1.52) |
| Namibia (2017) | HIV negative | 1.16 (1.09, 1.24) | 0.73 (0.64, 0.84) |
|  | On ART undetectable | 1.0 (Ref) | 1.0 (Ref) |
|  | On ART low-level viremia | 1.16 (0.91, 1.48) | 0.85 (0.66, 1.11) |
|  | On ART non-suppressed | 0.88 (0.69, 1.13) | 1.06 (0.80, 1.41) |
|  | Diagnosed but untreated | 1.34 (1.04, 1.74) | 1.39 (1.09, 1.79) |
|  | Undiagnosed | 1.89 (1.75, 2.05) | 1.63 (1.48, 1.80) |
| Nigeria (2018) | HIV negative | 0.90 (0.84, 0.97) | 0.64 (0.56, 0.73) |
|  | On ART undetectable | 1.0 (Ref) | 1.0 (Ref) |
|  | On ART low-level viremia | 1.37 (1.07, 1.76) | 0.93 (0.72, 1.20) |
|  | On ART non-suppressed | 1.14 (0.89, 1.48) | 0.89 (0.68, 1.17) |
|  | Diagnosed but untreated | 1.60 (1.23, 2.08) | 2.02 (1.58, 2.62) |
|  | Undiagnosed | 2.15 (1.98, 2.33) | 1.64 (1.49, 1.81) |
| Rwanda (2018-19) | HIV negative | 0.86 (0.80, 0.92) | 0.70 (0.61, 0.80) |
|  | On ART undetectable | 1.0 (Ref) | 1.0 (Ref) |
|  | On ART low-level viremia | 1.26 (0.98, 1.62) | 0.79 (0.61, 1.03) |
|  | On ART non-suppressed | 1.44 (1.11, 1.86) | 0.9 (0.68, 1.18) |
|  | Diagnosed but untreated | 1.11 (0.85, 1.44) | 2.01 (1.56, 2.63) |
|  | Undiagnosed | 1.83 (1.68, 1.98) | 1.74 (1.57, 1.92) |
| Tanzania (2016-17) | HIV negative | 0.86 (0.81, 0.92) | 0.63 (0.56, 0.71) |
|  | On ART undetectable | 1.0 (Ref) | 1.0 (Ref) |
|  | On ART low-level viremia | 1.3 (1.02, 1.67) | 0.83 (0.65, 1.07) |
|  | On ART non-suppressed | 0.99 (0.77, 1.28) | 0.87 (0.67, 1.13) |
|  | Diagnosed but untreated | 1.27 (0.98, 1.65) | 1.33 (1.05, 1.70) |
|  | Undiagnosed | 1.86 (1.72, 2.02) | 1.24 (1.14, 1.36) |
| Uganda (2016-17) | HIV negative | 0.87 (0.81, 0.94) | 0.66 (0.58, 0.75) |
|  | On ART undetectable | 1.0 (Ref) | 1.0 (Ref) |
|  | On ART low-level viremia | 1.32 (1.02, 1.69) | 0.95 (0.74, 1.24) |
|  | On ART non-suppressed | 0.96 (0.75, 1.25) | 0.93 (0.71, 1.22) |
|  | Diagnosed but untreated | 1.42 (1.09, 1.85) | 1.25 (0.98, 1.59) |
|  | Undiagnosed | 2.07 (1.91, 2.24) | 1.40 (1.27, 1.54) |
| Zambia (2016) | HIV negative | 0.94 (0.87, 1.01) | 0.69 (0.60, 0.79) |
|  | On ART undetectable | 1.0 (Ref) | 1.0 (Ref) |
|  | On ART low-level viremia | 1.14 (0.89, 1.47) | 0.84 (0.65, 1.10) |
|  | On ART non-suppressed | 1.08 (0.83, 1.40) | 0.9 (0.68, 1.19) |
|  | Diagnosed but untreated | 1.35 (1.04, 1.76) | 1.37 (1.07, 1.77) |
|  | Undiagnosed | 2.44 (2.24, 2.65) | 1.41 (1.28, 1.56) |
| Zimbabwe (2015-16) | HIV negative | 0.93 (0.88, 0.99) | 0.69 (0.61, 0.78) |
|  | On ART undetectable | 1.0 (Ref) | 1.0 (Ref) |
|  | On ART low-level viremia | 1.18 (0.93, 1.49) | 0.92 (0.71, 1.18) |
|  | On ART non-suppressed | 0.95 (0.75, 1.21) | 0.88 (0.67, 1.15) |
|  | Diagnosed but untreated | 1.23 (0.96, 1.58) | 1.49 (1.18, 1.90) |
|  | Undiagnosed | 2.08 (1.94, 2.24) | 1.42 (1.29, 1.55) |
