# Supplementary material for: HIV risk behaviour, viraemia, and transmission across HIV cascade stages including low-level viremia: Analysis of 14 cross-sectional population-based HIV Impact Assessment surveys in sub-Saharan Africa
Source: PLOS Glob Public Health. 2024 Apr 4;4(4):e0003030. doi: 10.1371/journal.pgph.0003030 (PMC10994324; doi:10.1371/journal.pgph.0003030)
Supplement: S8 Table — (DOCX) [file pgph.0003030.s008.docx]

**S8 Table. Predicted prevalence ratios of self-reporting condomless casual partnership for each of the 14 survey countries by sex** (Note: reference group is “On ART undetectable” group).

|  |  | **Predicted prevalence ratio (95% confidence interval) of self-reporting condomless casual partnership** | | |  |
| --- | --- | --- | --- | --- | --- |
| **Survey** | **Subgroup (Reference group: On ART undetectable)** | **Women** | **Men** | |  |
| Côte d'Ivoire (2017-18) | HIV negative | 0.44 (0.41, 0.47) | | 0.70 (0.62, 0.80) | |
|  | On ART undetectable | 1.0 (Ref) | | 1.0 (Ref) | |
|  | On ART low-level viremia | 0.89 (0.72, 1.09) | | 1.09 (0.84, 1.41) | |
|  | On ART non-suppressed | 0.36 (0.29, 0.43) | | 0.79 (0.61, 1.02) | |
|  | Diagnosed but untreated | 0.83 (0.69, 1.00) | | 0.57 (0.44, 0.74) | |
|  | Undiagnosed | 1.24 (1.16, 1.32) | | 1.10 (1.00, 1.20) | |
| Cameroon (2017-18) | HIV negative | 0.32 (0.29, 0.34) | | 0.63 (0.55, 0.72) | |
|  | On ART undetectable | 1.0 (Ref) | | 1.0 (Ref) | |
|  | On ART low-level viremia | 0.89 (0.72, 1.10) | | 1.14 (0.88, 1.49) | |
|  | On ART non-suppressed | 0.82 (0.67, 1.00) | | 0.72 (0.56, 0.94) | |
|  | Diagnosed but untreated | 1.22 (1.00, 1.49) | | 0.94 (0.71, 1.24) | |
|  | Undiagnosed | 1.07 (1.00, 1.15) | | 1.23 (1.12, 1.35) | |
| Eswatini (2016-17) | HIV negative | 0.57 (0.54, 0.61) | | 0.74 (0.65, 0.85) | |
|  | On ART undetectable | 1.0 (Ref) | | 1.0 (Ref) | |
|  | On ART low-level viremia | 0.71 (0.58, 0.86) | | 0.74 (0.58, 0.96) | |
|  | On ART non-suppressed | 0.76 (0.63, 0.93) | | 1.01 (0.78, 1.33) | |
|  | Diagnosed but untreated | 1.01 (0.84, 1.22) | | 1.05 (0.80, 1.40) | |
|  | Undiagnosed | 1.72 (1.60, 1.84) | | 1.57 (1.42, 1.73) | |
| Ethiopia (2017-18) | HIV negative | 0.35 (0.32, 0.37) | | 0.54 (0.47, 0.61) | |
|  | On ART undetectable | 1.0 (Ref) | | 1.0 (Ref) | |
|  | On ART low-level viremia | 0.90 (0.73, 1.11) | | 0.76 (0.59, 0.98) | |
|  | On ART non-suppressed | 0.73 (0.60, 0.89) | | 0.78 (0.60, 1.02) | |
|  | Diagnosed but untreated | 1.15 (0.94, 1.40) | | 0.72 (0.55, 0.96) | |
|  | Undiagnosed | 1.46 (1.37, 1.57) | | 1.03 (0.94, 1.13) | |
| Kenya (2018-19) | HIV negative | 0.31 (0.29, 0.33) | | 0.59 (0.52, 0.67) | |
|  | On ART undetectable | 1.0 (Ref) | | 1.0 (Ref) | |
|  | On ART low-level viremia | 0.77 (0.63, 0.95) | | 0.78 (0.60, 1.00) | |
|  | On ART non-suppressed | 0.69 (0.57, 0.85) | | 0.74 (0.57, 0.96) | |
|  | Diagnosed but untreated | 0.90 (0.74, 1.09) | | 1.54 (1.16, 2.06) | |
|  | Undiagnosed | 1.10 (1.02, 1.17) | | 1.33 (1.22, 1.46) | |
| Lesotho (2016-17) | HIV negative | 0.41 (0.38, 0.44) | | 0.71 (0.62, 0.81) | |
|  | On ART undetectable | 1.0 (Ref) | | 1.0 (Ref) | |
|  | On ART low-level viremia | 0.71 (0.58, 0.87) | | 0.78 (0.6, 1.01) | |
|  | On ART non-suppressed | 0.61 (0.50, 0.74) | | 0.79 (0.61, 1.03) | |
|  | Diagnosed but untreated | 0.85 (0.71, 1.04) | | 0.96 (0.73, 1.28) | |
|  | Undiagnosed | 1.26 (1.18, 1.35) | | 1.17 (1.07, 1.28) | |
| Malawi (2015-16) | HIV negative | 0.39 (0.36, 0.42) | | 0.66 (0.58, 0.75) | |
|  | On ART undetectable | 1.0 (Ref) | | 1.0 (Ref) | |
|  | On ART low-level viremia | 0.87 (0.70, 1.07) | | 0.75 (0.58, 0.96) | |
|  | On ART non-suppressed | 0.47 (0.38, 0.57) | | 0.83 (0.64, 1.08) | |
|  | Diagnosed but untreated | 1.16 (0.96, 1.41) | | 0.92 (0.70, 1.21) | |
|  | Undiagnosed | 1.23 (1.15, 1.31) | | 1.16 (1.06, 1.27) | |
| Namibia (2017) | HIV negative | 0.51 (0.47, 0.54) | | 0.66 (0.58, 0.75) | |
|  | On ART undetectable | 1.0 (Ref) | | 1.0 (Ref) | |
|  | On ART low-level viremia | 0.62 (0.51, 0.77) | | 0.72 (0.56, 0.94) | |
|  | On ART non-suppressed | 0.50 (0.41, 0.61) | | 0.99 (0.76, 1.31) | |
|  | Diagnosed but untreated | 1.06 (0.87, 1.29) | | 0.83 (0.63, 1.10) | |
|  | Undiagnosed | 1.05 (0.98, 1.13) | | 1.43 (1.29, 1.58) | |
| Nigeria (2018) | HIV negative | 0.34 (0.31, 0.36) | | 0.58 (0.51, 0.66) | |
|  | On ART undetectable | 1.0 (Ref) | | 1.0 (Ref) | |
|  | On ART low-level viremia | 0.86 (0.70, 1.05) | | 0.81 (0.63, 1.04) | |
|  | On ART non-suppressed | 0.89 (0.73, 1.08) | | 0.76 (0.59, 0.99) | |
|  | Diagnosed but untreated | 1.32 (1.09, 1.60) | | 1.48 (1.12, 1.97) | |
|  | Undiagnosed | 1.40 (1.31, 1.49) | | 1.55 (1.41, 1.70) | |
| Rwanda (2018-19) | HIV negative | 0.33 (0.31, 0.36) | | 0.66 (0.58, 0.75) | |
|  | On ART undetectable | 1.0 (Ref) | | 1.0 (Ref) | |
|  | On ART low-level viremia | 0.94 (0.76, 1.17) | | 0.65 (0.50, 0.83) | |
|  | On ART non-suppressed | 1.32 (1.07, 1.63) | | 0.76 (0.59, 1.00) | |
|  | Diagnosed but untreated | 0.80 (0.66, 0.97) | | 1.60 (1.20, 2.15) | |
|  | Undiagnosed | 1.09 (1.01, 1.17) | | 1.90 (1.73, 2.10) | |
| Tanzania (2016-17) | HIV negative | 0.34 (0.32, 0.36) | | 0.56 (0.49, 0.63) | |
|  | On ART undetectable | 1.0 (Ref) | | 1.0 (Ref) | |
|  | On ART low-level viremia | 0.73 (0.60, 0.90) | | 0.68 (0.53, 0.87) | |
|  | On ART non-suppressed | 0.73 (0.60, 0.89) | | 0.72 (0.56, 0.94) | |
|  | Diagnosed but untreated | 0.82 (0.68, 1.00) | | 0.86 (0.65, 1.13) | |
|  | Undiagnosed | 1.09 (1.02, 1.17) | | 1.05 (0.96, 1.14) | |
| Uganda (2016-17) | HIV negative | 0.34 (0.31, 0.36) | | 0.6 (0.53, 0.68) | |
|  | On ART undetectable | 1.0 (Ref) | | 1.0 (Ref) | |
|  | On ART low-level viremia | 0.83 (0.67, 1.02) | | 0.8 (0.62, 1.03) | |
|  | On ART non-suppressed | 0.61 (0.50, 0.74) | | 0.78 (0.60, 1.01) | |
|  | Diagnosed but untreated | 1.03 (0.85, 1.26) | | 0.81 (0.62, 1.07) | |
|  | Undiagnosed | 1.12 (1.04, 1.20) | | 1.17 (1.07, 1.29) | |
| Zambia (2016) | HIV negative | 0.39 (0.37, 0.42) | | 0.67 (0.59, 0.77) | |
|  | On ART undetectable | 1.0 (Ref) | | 1.0 (Ref) | |
|  | On ART low-level viremia | 0.59 (0.48, 0.73) | | 0.69 (0.54, 0.89) | |
|  | On ART non-suppressed | 0.75 (0.62, 0.92) | | 0.82 (0.63, 1.07) | |
|  | Diagnosed but untreated | 1.17 (0.96, 1.42) | | 0.92 (0.70, 1.22) | |
|  | Undiagnosed | 1.71 (1.60, 1.83) | | 1.24 (1.13, 1.36) | |
| Zimbabwe (2015-16) | HIV negative | 0.34 (0.32, 0.37) | | 0.63 (0.56, 0.72) | |
|  | On ART undetectable | 1.0 (Ref) | | 1.0 (Ref) | |
|  | On ART low-level viremia | 0.63 (0.52, 0.77) | | 0.78 (0.61, 1.00) | |
|  | On ART non-suppressed | 0.59 (0.49, 0.72) | | 0.76 (0.59, 0.99) | |
|  | Diagnosed but untreated | 0.77 (0.64, 0.93) | | 1.06 (0.81, 1.40) | |
|  | Undiagnosed | 1.19 (1.12, 1.27) | | 1.21 (1.11, 1.33) | |
