# Supplementary material for: HIV risk behaviour, viraemia, and transmission across HIV cascade stages including low-level viremia: Analysis of 14 cross-sectional population-based HIV Impact Assessment surveys in sub-Saharan Africa
Source: PLOS Glob Public Health. 2024 Apr 4;4(4):e0003030. doi: 10.1371/journal.pgph.0003030 (PMC10994324; doi:10.1371/journal.pgph.0003030)
Supplement: S9 Table — (DOCX) [file pgph.0003030.s009.docx]

**S9 Table. Predicted prevalence ratios of self-reporting transactional partnership for each of the 14 survey countries by sex.** (Note: reference group is “On ART undetectable” group).

|  |  | **Predicted prevalence ratio (95% confidence interval) of self-reporting transactional partnership** | |
| --- | --- | --- | --- |
| **Survey** | **Subgroup (Reference group: On ART undetectable)** | **Women** | **Men** |
| Côte d'Ivoire (2017-18) | HIV negative | 1.18 (1.14, 1.23) | 0.88 (0.75, 1.04) |
|  | On ART undetectable | 1.0 (Ref) | 1.0 (Ref) |
|  | On ART low-level viremia | 1.18 (1.01, 1.37) | 0.71 (0.45, 1.12) |
|  | On ART non-suppressed | 0.99 (0.85, 1.14) | 1.02 (0.70, 1.49) |
|  | Diagnosed but untreated | 1.32 (1.13, 1.55) | 0.89 (0.52, 1.53) |
|  | Undiagnosed | 1.34 (1.26, 1.43) | 1.25 (1.07, 1.48) |
| Cameroon (2017-18) | HIV negative | 1.20 (1.16, 1.25) | 0.97 (0.83, 1.14) |
|  | On ART undetectable | 1.0 (Ref) | 1.0 (Ref) |
|  | On ART low-level viremia | 1.26 (1.08, 1.47) | 0.66 (0.42, 1.04) |
|  | On ART non-suppressed | 1.16 (1.01, 1.35) | 1.15 (0.79, 1.68) |
|  | Diagnosed but untreated | 1.52 (1.30, 1.79) | 0.76 (0.45, 1.29) |
|  | Undiagnosed | 1.42 (1.33, 1.52) | 1.32 (1.13, 1.55) |
| Eswatini (2016-17) | HIV negative | 1.22 (1.18, 1.26) | 0.90 (0.76, 1.06) |
|  | On ART undetectable | 1.0 (Ref) | 1.0 (Ref) |
|  | On ART low-level viremia | 1.16 (1.00, 1.34) | 0.66 (0.42, 1.04) |
|  | On ART non-suppressed | 1.17 (1.01, 1.35) | 1.03 (0.71, 1.52) |
|  | Diagnosed but untreated | 1.36 (1.17, 1.60) | 0.72 (0.43, 1.23) |
|  | Undiagnosed | 1.36 (1.28, 1.46) | 1.26 (1.07, 1.48) |
| Ethiopia (2017-18) | HIV negative | 1.12 (1.08, 1.16) | 0.96 (0.82, 1.11) |
|  | On ART undetectable | 1.0 (Ref) | 1.0 (Ref) |
|  | On ART low-level viremia | 1.32 (1.13, 1.54) | 0.67 (0.43, 1.04) |
|  | On ART non-suppressed | 1.14 (0.98, 1.32) | 1.18 (0.82, 1.71) |
|  | Diagnosed but untreated | 1.48 (1.26, 1.75) | 0.86 (0.51, 1.46) |
|  | Undiagnosed | 1.34 (1.26, 1.44) | 1.33 (1.15, 1.55) |
| Kenya (2018-19) | HIV negative | 1.15 (1.11, 1.19) | 0.90 (0.78, 1.06) |
|  | On ART undetectable | 1.0 (Ref) | 1.0 (Ref) |
|  | On ART low-level viremia | 1.20 (1.03, 1.40) | 0.63 (0.40, 0.99) |
|  | On ART non-suppressed | 1.10 (0.95, 1.27) | 1.19 (0.82, 1.75) |
|  | Diagnosed but untreated | 1.41 (1.21, 1.66) | 0.84 (0.50, 1.44) |
|  | Undiagnosed | 1.32 (1.23, 1.41) | 1.25 (1.07, 1.46) |
| Lesotho (2016-17) | HIV negative | 1.18 (1.14, 1.23) | 0.91 (0.79, 1.05) |
|  | On ART undetectable | 1.0 (Ref) | 1.0 (Ref) |
|  | On ART low-level viremia | 1.16 (1.00, 1.36) | 0.65 (0.42, 1.02) |
|  | On ART non-suppressed | 1.13 (0.98, 1.31) | 1.12 (0.78, 1.62) |
|  | Diagnosed but untreated | 1.35 (1.16, 1.59) | 0.74 (0.44, 1.24) |
|  | Undiagnosed | 1.39 (1.30, 1.48) | 1.28 (1.10, 1.48) |
| Malawi (2015-16) | HIV negative | 1.23 (1.18, 1.28) | 0.94 (0.81, 1.10) |
|  | On ART undetectable | 1.0 (Ref) | 1.0 (Ref) |
|  | On ART low-level viremia | 1.24 (1.06, 1.44) | 0.71 (0.46, 1.11) |
|  | On ART non-suppressed | 1.03 (0.89, 1.19) | 1.05 (0.73, 1.52) |
|  | Diagnosed but untreated | 1.42 (1.22, 1.67) | 0.78 (0.47, 1.31) |
|  | Undiagnosed | 1.32 (1.23, 1.41) | 1.26 (1.08, 1.46) |
| Namibia (2017) | HIV negative | 1.15 (1.11, 1.20) | 0.88 (0.75, 1.03) |
|  | On ART undetectable | 1.0 (Ref) | 1.0 (Ref) |
|  | On ART low-level viremia | 1.12 (0.96, 1.30) | 0.66 (0.42, 1.05) |
|  | On ART non-suppressed | 1.05 (0.91, 1.22) | 1.07 (0.74, 1.57) |
|  | Diagnosed but untreated | 1.36 (1.16, 1.60) | 0.71 (0.42, 1.20) |
|  | Undiagnosed | 1.29 (1.21, 1.38) | 1.22 (1.04, 1.43) |
| Nigeria (2018) | HIV negative | 1.21 (1.16, 1.26) | 0.95 (0.81, 1.11) |
|  | On ART undetectable | 1.0 (Ref) | 1.0 (Ref) |
|  | On ART low-level viremia | 1.21 (1.04, 1.41) | 0.66 (0.42, 1.04) |
|  | On ART non-suppressed | 1.20 (1.04, 1.39) | 1.10 (0.75, 1.61) |
|  | Diagnosed but untreated | 1.47 (1.26, 1.73) | 0.79 (0.47, 1.35) |
|  | Undiagnosed | 1.44 (1.35, 1.55) | 1.22 (1.04, 1.44) |
| Rwanda (2018-19) | HIV negative | 1.21 (1.17, 1.26) | 0.88 (0.75, 1.03) |
|  | On ART undetectable | 1.0 (Ref) | 1.0 (Ref) |
|  | On ART low-level viremia | 1.19 (1.02, 1.39) | 0.63 (0.41, 1.00) |
|  | On ART non-suppressed | 1.26 (1.09, 1.46) | 0.99 (0.68, 1.44) |
|  | Diagnosed but untreated | 1.42 (1.21, 1.67) | 0.68 (0.40, 1.14) |
|  | Undiagnosed | 1.38 (1.29, 1.48) | 1.32 (1.13, 1.55) |
| Tanzania (2016-17) | HIV negative | 1.20 (1.16, 1.24) | 0.94 (0.81, 1.09) |
|  | On ART undetectable | 1.0 (Ref) | 1.0 (Ref) |
|  | On ART low-level viremia | 1.18 (1.02, 1.38) | 0.65 (0.42, 1.01) |
|  | On ART non-suppressed | 1.09 (0.94, 1.27) | 1.10 (0.77, 1.60) |
|  | Diagnosed but untreated | 1.41 (1.20, 1.66) | 0.80 (0.48, 1.36) |
|  | Undiagnosed | 1.39 (1.30, 1.48) | 1.37 (1.19, 1.60) |
| Uganda (2016-17) | HIV negative | 1.20 (1.15, 1.24) | 0.94 (0.81, 1.09) |
|  | On ART undetectable | 1.0 (Ref) | 1.0 (Ref) |
|  | On ART low-level viremia | 1.16 (1.00, 1.35) | 0.65 (0.42, 1.01) |
|  | On ART non-suppressed | 1.11 (0.96, 1.29) | 1.08 (0.75, 1.57) |
|  | Diagnosed but untreated | 1.42 (1.21, 1.68) | 0.83 (0.49, 1.40) |
|  | Undiagnosed | 1.37 (1.28, 1.46) | 1.27 (1.09, 1.47) |
| Zambia (2016) | HIV negative | 1.29 (1.24, 1.34) | 0.97 (0.83, 1.13) |
|  | On ART undetectable | 1.0 (Ref) | 1.0 (Ref) |
|  | On ART low-level viremia | 1.11 (0.96, 1.30) | 0.67 (0.43, 1.05) |
|  | On ART non-suppressed | 1.16 (1.00, 1.34) | 1.10 (0.76, 1.60) |
|  | Diagnosed but untreated | 1.44 (1.23, 1.70) | 0.79 (0.47, 1.34) |
|  | Undiagnosed | 1.53 (1.43, 1.64) | 1.30 (1.12, 1.52) |
| Zimbabwe (2015-16) | HIV negative | 1.18 (1.14, 1.22) | 0.91 (0.78, 1.07) |
|  | On ART undetectable | 1.0 (Ref) | 1.0 (Ref) |
|  | On ART low-level viremia | 1.14 (0.98, 1.32) | 0.65 (0.42, 1.02) |
|  | On ART non-suppressed | 1.12 (0.97, 1.29) | 1.10 (0.76, 1.61) |
|  | Diagnosed but untreated | 1.36 (1.17, 1.59) | 0.75 (0.44, 1.27) |
|  | Undiagnosed | 1.36 (1.28, 1.45) | 1.26 (1.08, 1.48) |
