# Supplementary material for: HIV risk behaviour, viraemia, and transmission across HIV cascade stages including low-level viremia: Analysis of 14 cross-sectional population-based HIV Impact Assessment surveys in sub-Saharan Africa
Source: PLOS Glob Public Health. 2024 Apr 4;4(4):e0003030. doi: 10.1371/journal.pgph.0003030 (PMC10994324; doi:10.1371/journal.pgph.0003030)
Supplement: S10 Table — (DOCX) [file pgph.0003030.s010.docx]

**S10 Table. Predicted prevalence ratios of self-reporting condomless last sex (with any partner) for each of the 14 survey countries by sex** (Note: reference group is “On ART undetectable” group).

|  |  | **Predicted prevalence ratio (95% confidence interval) of self-reporting condomless last sex** | |
| --- | --- | --- | --- |
| **Survey** | **Subgroup (Reference group: On ART undetectable)** | **Women** | **Men** |
| Côte d'Ivoire (2017-18) | HIV negative | 0.56 (0.55, 0.58) | 0.19 (0.18, 0.2) |
|  | On ART undetectable | 1.0 (Ref) | 1.0 (Ref) |
|  | On ART low-level viremia | 0.38 (0.36, 0.41) | 0.22 (0.2, 0.25) |
|  | On ART non-suppressed | 0.79 (0.72, 0.88) | 0.31 (0.28, 0.35) |
|  | Diagnosed but untreated | 0.60 (0.56, 0.66) | 0.71 (0.58, 0.89) |
|  | Undiagnosed | 1.03 (1.00, 1.06) | 0.72 (0.68, 0.76) |
| Cameroon (2017-18) | HIV negative | 0.69 (0.67, 0.70) | 0.21 (0.21, 0.22) |
|  | On ART undetectable | 1.0 (Ref) | 1.0 (Ref) |
|  | On ART low-level viremia | 0.44 (0.41, 0.47) | 0.25 (0.22, 0.27) |
|  | On ART non-suppressed | 0.53 (0.50, 0.57) | 0.39 (0.34, 0.45) |
|  | Diagnosed but untreated | 0.61 (0.56, 0.65) | 0.48 (0.42, 0.56) |
|  | Undiagnosed | 1.26 (1.23, 1.29) | 0.71 (0.67, 0.75) |
| Eswatini (2016-17) | HIV negative | 0.43 (0.42, 0.44) | 0.22 (0.21, 0.23) |
|  | On ART undetectable | 1.0 (Ref) | 1.0 (Ref) |
|  | On ART low-level viremia | 0.49 (0.45, 0.53) | 0.39 (0.35, 0.44) |
|  | On ART non-suppressed | 0.52 (0.48, 0.56) | 0.34 (0.3, 0.38) |
|  | Diagnosed but untreated | 0.74 (0.68, 0.81) | 0.49 (0.43, 0.57) |
|  | Undiagnosed | 1.03 (1.00, 1.06) | 0.58 (0.55, 0.6) |
| Ethiopia (2017-18) | HIV negative | 0.68 (0.67, 0.69) | 0.28 (0.27, 0.29) |
|  | On ART undetectable | 1.0 (Ref) | 1.0 (Ref) |
|  | On ART low-level viremia | 0.53 (0.49, 0.57) | 0.41 (0.36, 0.46) |
|  | On ART non-suppressed | 0.46 (0.43, 0.49) | 0.39 (0.35, 0.44) |
|  | Diagnosed but untreated | 0.51 (0.48, 0.55) | 0.79 (0.67, 0.96) |
|  | Undiagnosed | 0.94 (0.92, 0.96) | 1.07 (1.01, 1.13) |
| Kenya (2018-19) | HIV negative | 0.73 (0.71, 0.75) | 0.23 (0.23, 0.24) |
|  | On ART undetectable | 1.0 (Ref) | 1.0 (Ref) |
|  | On ART low-level viremia | 0.52 (0.48, 0.56) | 0.33 (0.29, 0.37) |
|  | On ART non-suppressed | 0.59 (0.55, 0.63) | 0.35 (0.31, 0.4) |
|  | Diagnosed but untreated | 0.83 (0.76, 0.90) | 0.3 (0.27, 0.33) |
|  | Undiagnosed | 1.09 (1.07, 1.12) | 0.7 (0.66, 0.74) |
| Lesotho (2016-17) | HIV negative | 0.64 (0.63, 0.66) | 0.25 (0.25, 0.26) |
|  | On ART undetectable | 1.0 (Ref) | 1.0 (Ref) |
|  | On ART low-level viremia | 0.48 (0.45, 0.52) | 0.37 (0.34, 0.41) |
|  | On ART non-suppressed | 0.64 (0.60, 0.69) | 0.41 (0.37, 0.46) |
|  | Diagnosed but untreated | 0.80 (0.74, 0.88) | 0.57 (0.5, 0.65) |
|  | Undiagnosed | 1.11 (1.08, 1.13) | 0.92 (0.88, 0.96) |
| Malawi (2015-16) | HIV negative | 0.61 (0.59, 0.62) | 0.2 (0.2, 0.21) |
|  | On ART undetectable | 1.0 (Ref) | 1.0 (Ref) |
|  | On ART low-level viremia | 0.40 (0.37, 0.43) | 0.35 (0.31, 0.39) |
|  | On ART non-suppressed | 0.64 (0.59, 0.69) | 0.33 (0.3, 0.38) |
|  | Diagnosed but untreated | 0.61 (0.56, 0.67) | 0.47 (0.41, 0.56) |
|  | Undiagnosed | 1.05 (1.02, 1.08) | 0.79 (0.74, 0.84) |
| Namibia (2017) | HIV negative | 0.47 (0.46, 0.48) | 0.28 (0.27, 0.29) |
|  |  |  |  |
|  | On ART low-level viremia | 0.49 (0.45, 0.53) | 0.42 (0.38, 0.47) |
|  | On ART non-suppressed | 0.58 (0.54, 0.63) | 0.35 (0.32, 0.39) |
|  | Diagnosed but untreated | 0.57 (0.53, 0.62) | 0.66 (0.58, 0.76) |
|  | Undiagnosed | 1.09 (1.05, 1.12) | 0.72 (0.7, 0.75) |
| Nigeria (2018) | HIV negative | 0.64 (0.62, 0.65) | 0.22 (0.21, 0.23) |
|  | On ART undetectable | 1.0 (Ref) | 1.0 (Ref) |
|  | On ART low-level viremia | 0.43 (0.40, 0.47) | 0.31 (0.27, 0.35) |
|  | On ART non-suppressed | 0.46 (0.43, 0.50) | 0.35 (0.31, 0.4) |
|  | Diagnosed but untreated | 0.69 (0.64, 0.76) | 0.31 (0.28, 0.36) |
|  | Undiagnosed | 1.03 (1.01, 1.06) | 0.54 (0.52, 0.57) |
| Rwanda (2018-19) | HIV negative | 0.69 (0.68, 0.71) | 0.21 (0.2, 0.22) |
|  | On ART undetectable | 1.0 (Ref) | 1.0 (Ref) |
|  | On ART low-level viremia | 0.38 (0.35, 0.40) | 0.38 (0.33, 0.44) |
|  | On ART non-suppressed | 0.43 (0.41, 0.46) | 0.38 (0.33, 0.44) |
|  | Diagnosed but untreated | 0.98 (0.90, 1.08) | 0.29 (0.26, 0.33) |
|  | Undiagnosed | 1.12 (1.10, 1.15) | 0.38 (0.36, 0.39) |
| Tanzania (2016-17) | HIV negative | 0.64 (0.63, 0.65) | 0.26 (0.25, 0.27) |
|  | On ART undetectable | 1.0 (Ref) | 1.0 (Ref) |
|  | On ART low-level viremia | 0.51 (0.47, 0.55) | 0.4 (0.36, 0.45) |
|  | On ART non-suppressed | 0.46 (0.43, 0.49) | 0.41 (0.36, 0.47) |
|  | Diagnosed but untreated | 0.90 (0.82, 0.99) | 0.6 (0.51, 0.7) |
|  | Undiagnosed | 1.07 (1.04, 1.10) | 0.9 (0.86, 0.95) |
| Uganda (2016-17) | HIV negative | 0.66 (0.64, 0.67) | 0.25 (0.24, 0.26) |
|  | On ART undetectable | 1.0 (Ref) | 1.0 (Ref) |
|  | On ART low-level viremia | 0.40 (0.37, 0.42) | 0.37 (0.33, 0.41) |
|  | On ART non-suppressed | 0.56 (0.52, 0.60) | 0.41 (0.37, 0.47) |
|  | Diagnosed but untreated | 0.74 (0.68, 0.81) | 0.60 (0.52, 0.70) |
|  | Undiagnosed | 1.32 (1.28, 1.36) | 0.89 (0.85, 0.93) |
| Zambia (2016) | HIV negative | 0.61 (0.59, 0.62) | 0.21 (0.20, 0.22) |
|  | On ART undetectable | 1.0 (Ref) | 1.0 (Ref) |
|  | On ART low-level viremia | 0.50 (0.46, 0.55) | 0.39 (0.34, 0.44) |
|  | On ART non-suppressed | 0.52 (0.48, 0.56) | 0.33 (0.29, 0.37) |
|  | Diagnosed but untreated | 0.56 (0.52, 0.61) | 0.50 (0.43, 0.59) |
|  | Undiagnosed | 0.91 (0.89, 0.94) | 0.75 (0.71, 0.79) |
| Zimbabwe (2015-16) | HIV negative | 0.69 (0.68, 0.71) | 0.22 (0.21, 0.23) |
|  | On ART undetectable | 1.0 (Ref) | 1.0 (Ref) |
|  | On ART low-level viremia | 0.55 (0.52, 0.60) | 0.34 (0.30, 0.38) |
|  | On ART non-suppressed | 0.62 (0.58, 0.66) | 0.36 (0.32, 0.41) |
|  | Diagnosed but untreated | 0.89 (0.82, 0.97) | 0.41 (0.36, 0.48) |
|  | Undiagnosed | 1.13 (1.11, 1.16) | 0.76 (0.72, 0.81) |
