# Supplementary material for: HIV risk behaviour, viraemia, and transmission across HIV cascade stages including low-level viremia: Analysis of 14 cross-sectional population-based HIV Impact Assessment surveys in sub-Saharan Africa
Source: PLOS Glob Public Health. 2024 Apr 4;4(4):e0003030. doi: 10.1371/journal.pgph.0003030 (PMC10994324; doi:10.1371/journal.pgph.0003030)
Supplement: S11 Table — (DOCX) [file pgph.0003030.s011.docx]

**S11 Table.** **Predicted prevalence ratios of self-reporting both multiple sexual partnership and condomless last sex for each of the 14 survey countries by sex** (Note: reference group is “On ART undetectable” group).

|  |  | **Predicted prevalence ratio (95% confidence interval) of self-reporting both multiple sexual partnership and condomless last sex** | |
| --- | --- | --- | --- |
| **Survey** | **Subgroup (Reference group: On ART undetectable)** | **Women** | **Men** |
| Côte d'Ivoire (2017-18) | HIV negative | 0.92 (0.86, 1.00) | 0.43 (0.39, 0.47) |
|  | On ART undetectable | 1.0 (Ref) | 1.0 (Ref) |
|  | On ART low-level viremia | 1.23 (0.96, 1.58) | 0.62 (0.50, 0.78) |
|  | On ART non-suppressed | 0.94 (0.74, 1.18) | 0.49 (0.39, 0.63) |
|  | Diagnosed but untreated | 1.43 (1.14, 1.81) | 1.14 (0.90, 1.46) |
|  | Undiagnosed | 2.25 (2.08, 2.43) | 1.21 (1.11, 1.32) |
| Cameroon (2017-18) | HIV negative | 0.81 (0.75, 0.88) | 0.45 (0.41, 0.50) |
|  | On ART undetectable | 1.0 (Ref) | 1.0 (Ref) |
|  | On ART low-level viremia | 1.19 (0.92, 1.54) | 0.57 (0.46, 0.71) |
|  | On ART non-suppressed | 1.27 (1.00, 1.61) | 0.57 (0.45, 0.73) |
|  | Diagnosed but untreated | 1.60 (1.26, 2.02) | 0.95 (0.76, 1.20) |
|  | Undiagnosed | 2.18 (2.02, 2.36) | 1.25 (1.15, 1.36) |
| Eswatini (2016-17) | HIV negative | 1.00 (0.93, 1.07) | 0.41 (0.37, 0.46) |
|  | On ART undetectable | 1.0 (Ref) | 1.0 (Ref) |
|  | On ART low-level viremia | 1.18 (0.93, 1.51) | 0.58 (0.47, 0.72) |
|  | On ART non-suppressed | 1.20 (0.95, 1.50) | 0.51 (0.40, 0.66) |
|  | Diagnosed but untreated | 1.65 (1.32, 2.08) | 0.92 (0.74, 1.17) |
|  | Undiagnosed | 2.65 (2.44, 2.87) | 1.18 (1.08, 1.29) |
| Ethiopia (2017-18) | HIV negative | 0.85 (0.78, 0.92) | 0.45 (0.41, 0.49) |
|  | On ART undetectable | 1.0 (Ref) | 1.0 (Ref) |
|  | On ART low-level viremia | 1.41 (1.09, 1.83) | 0.61 (0.50, 0.76) |
|  | On ART non-suppressed | 1.11 (0.88, 1.41) | 0.58 (0.46, 0.73) |
|  | Diagnosed but untreated | 1.64 (1.29, 2.08) | 1.03 (0.82, 1.30) |
|  | Undiagnosed | 2.45 (2.27, 2.66) | 1.32 (1.22, 1.43) |
| Kenya (2018-19) | HIV negative | 0.84 (0.78, 0.90) | 0.44 (0.39, 0.48) |
|  | On ART undetectable | 1.0 (Ref) | 1.0 (Ref) |
|  | On ART low-level viremia | 1.25 (0.98, 1.61) | 0.56 (0.45, 0.69) |
|  | On ART non-suppressed | 1.21 (0.96, 1.53) | 0.55 (0.43, 0.70) |
|  | Diagnosed but untreated | 1.63 (1.30, 2.06) | 0.97 (0.77, 1.23) |
|  | Undiagnosed | 2.15 (1.99, 2.32) | 1.22 (1.12, 1.33) |
| Lesotho (2016-17) | HIV negative | 0.93 (0.86, 1.00) | 0.44 (0.40, 0.48) |
|  | On ART undetectable | 1.0 (Ref) | 1.0 (Ref) |
|  | On ART low-level viremia | 1.15 (0.90, 1.48) | 0.56 (0.46, 0.69) |
|  | On ART non-suppressed | 1.15 (0.92, 1.46) | 0.56 (0.44, 0.71) |
|  | Diagnosed but untreated | 1.57 (1.25, 1.98) | 0.95 (0.77, 1.19) |
|  | Undiagnosed | 2.32 (2.15, 2.50) | 1.28 (1.18, 1.38) |
| Malawi (2015-16) | HIV negative | 0.88 (0.81, 0.95) | 0.44 (0.40, 0.49) |
|  | On ART undetectable | 1.0 (Ref) | 1.0 (Ref) |
|  | On ART low-level viremia | 1.22 (0.94, 1.57) | 0.63 (0.51, 0.79) |
|  | On ART non-suppressed | 1.00 (0.79, 1.27) | 0.53 (0.42, 0.68) |
|  | Diagnosed but untreated | 1.76 (1.39, 2.23) | 0.96 (0.77, 1.20) |
|  | Undiagnosed | 2.25 (2.08, 2.44) | 1.22 (1.13, 1.32) |
| Namibia (2017) | HIV negative | 0.99 (0.92, 1.06) | 0.42 (0.38, 0.46) |
|  | On ART undetectable | 1.0 (Ref) | 1.0 (Ref) |
|  | On ART low-level viremia | 1.10 (0.86, 1.42) | 0.57 (0.47, 0.71) |
|  | On ART non-suppressed | 1.03 (0.82, 1.30) | 0.51 (0.40, 0.65) |
|  | Diagnosed but untreated | 1.63 (1.29, 2.06) | 0.91 (0.73, 1.15) |
|  | Undiagnosed | 2.13 (1.97, 2.30) | 1.19 (1.10, 1.3) |
| Nigeria (2018) | HIV negative | 0.85 (0.78, 0.92) | 0.45 (0.41, 0.50) |
|  | On ART undetectable | 1.0 (Ref) | 1.0 (Ref) |
|  | On ART low-level viremia | 1.24 (0.97, 1.60) | 0.58 (0.47, 0.72) |
|  | On ART non-suppressed | 1.25 (0.99, 1.58) | 0.53 (0.42, 0.69) |
|  | Diagnosed but untreated | 1.86 (1.48, 2.36) | 0.93 (0.74, 1.18) |
|  | Undiagnosed | 2.33 (2.15, 2.52) | 1.19 (1.09, 1.30) |
| Rwanda (2018-19) | HIV negative | 0.81 (0.75, 0.87) | 0.42 (0.38, 0.46) |
|  | On ART undetectable | 1.0 (Ref) | 1.0 (Ref) |
|  | On ART low-level viremia | 1.15 (0.89, 1.49) | 0.58 (0.47, 0.72) |
|  | On ART non-suppressed | 1.48 (1.17, 1.88) | 0.52 (0.41, 0.66) |
|  | Diagnosed but untreated | 1.44 (1.14, 1.82) | 0.88 (0.70, 1.11) |
|  | Undiagnosed | 2.07 (1.92, 2.24) | 1.15 (1.05, 1.25) |
| Tanzania (2016-17) | HIV negative | 0.82 (0.76, 0.88) | 0.45 (0.41, 0.49) |
|  | On ART undetectable | 1.0 (Ref) | 1.0 (Ref) |
|  | On ART low-level viremia | 1.21 (0.94, 1.56) | 0.59 (0.48, 0.73) |
|  | On ART non-suppressed | 1.11 (0.87, 1.40) | 0.55 (0.44, 0.71) |
|  | Diagnosed but untreated | 1.59 (1.26, 2.01) | 1.03 (0.82, 1.30) |
|  | Undiagnosed | 2.12 (1.96, 2.29) | 1.32 (1.22, 1.43) |
| Uganda (2016-17) | HIV negative | 0.83 (0.77, 0.9) | 0.45 (0.41, 0.49) |
|  | On ART undetectable | 1.0 (Ref) | 1.0 (Ref) |
|  | On ART low-level viremia | 1.20 (0.93, 1.56) | 0.59 (0.48, 0.73) |
|  | On ART non-suppressed | 1.10 (0.87, 1.40) | 0.55 (0.44, 0.70) |
|  | Diagnosed but untreated | 1.74 (1.38, 2.21) | 1.00 (0.81, 1.26) |
|  | Undiagnosed | 2.29 (2.12, 2.48) | 1.27 (1.18, 1.38) |
| Zambia (2016) | HIV negative | 0.87 (0.80, 0.94) | 0.45 (0.41, 0.50) |
|  | On ART undetectable | 1.0 (Ref) | 1.0 (Ref) |
|  | On ART low-level viremia | 1.09 (0.85, 1.41) | 0.60 (0.49, 0.74) |
|  | On ART non-suppressed | 1.20 (0.95, 1.53) | 0.53 (0.42, 0.68) |
|  | Diagnosed but untreated | 1.67 (1.32, 2.12) | 1.00 (0.80, 1.26) |
|  | Undiagnosed | 2.59 (2.39, 2.81) | 1.25 (1.15, 1.35) |
| Zimbabwe (2015-16) | HIV negative | 0.86 (0.80, 0.92) | 0.43 (0.39, 0.48) |
|  | On ART undetectable | 1.0 (Ref) | 1.0 (Ref) |
|  | On ART low-level viremia | 1.13 (0.89, 1.45) | 0.58 (0.47, 0.72) |
|  | On ART non-suppressed | 1.09 (0.87, 1.37) | 0.54 (0.42, 0.69) |
|  | Diagnosed but untreated | 1.55 (1.24, 1.94) | 0.91 (0.73, 1.15) |
|  | Undiagnosed | 2.29 (2.13, 2.46) | 1.22 (1.12, 1.33) |
