# Supplementary material for: HIV risk behaviour, viraemia, and transmission across HIV cascade stages including low-level viremia: Analysis of 14 cross-sectional population-based HIV Impact Assessment surveys in sub-Saharan Africa
Source: PLOS Glob Public Health. 2024 Apr 4;4(4):e0003030. doi: 10.1371/journal.pgph.0003030 (PMC10994324; doi:10.1371/journal.pgph.0003030)
Supplement: S12 Table — (DOCX) [file pgph.0003030.s012.docx]

**S12 Table. Mean log_10_ viral load and transmission rate per year for all PLHIV subgroups by sex.** Transmission rate estimated using the Hill function from Fraser et al.

|  |  | **Women** | | **Men** | |
| --- | --- | --- | --- | --- | --- |
| **Survey** | **PLHIV subgroup** | **Mean log_10_ viral load (copies/mL)** | **Mean annual transmission rate (per year)** | **Mean log_10_ viral load (copies/mL)** | **Mean annual transmission rate (per year)** |
| Cameroon  (2017-18) | On ART low-level viremia | 2.24 | 0.005 | 2.05 | 0.003 |
|  | On ART non-suppressed | 4.35 | 0.17 | 4.71 | 0.20 |
|  | Diagnosed but untreated | 4.81 | 0.23 | 5.18 | 0.27 |
|  | Undiagnosed | 4.79 | 0.23 | 5.19 | 0.27 |
| Côte d’Ivoire (2017-18) | On ART low-level viremia | 2.11 | 0.004 | 2.25 | 0.006 |
|  | On ART non-suppressed | 4.47 | 0.21 | 4.95 | 0.26 |
|  | Diagnosed but untreated | 4.47 | 0.19 | 5.32 | 0.28 |
|  | Undiagnosed | 4.66 | 0.22 | 4.68 | 0.22 |
| Nigeria (2018) | On ART low-level viremia | 2.16 | 0.004 | 2.05 | 0.004 |
|  | On ART non-suppressed | 4.47 | 0.20 | 4.38 | 0.18 |
|  | Diagnosed but untreated | 4.17 | 0.17 | 4.99 | 0.27 |
|  | Undiagnosed | 4.45 | 0.20 | 4.65 | 0.22 |
| Ethiopia (2017-18) | On ART low-level viremia | 2.13 | 0.004 | 2.04 | 0.003 |
|  | On ART non-suppressed | 4.31 | 0.18 | 4.62 | 0.21 |
|  | Diagnosed but untreated | 4.60 | 0.19 | 5.31 | 0.30 |
|  | Undiagnosed | 4.31 | 0.19 | 4.54 | 0.21 |
| Kenya  (2018-19) | On ART low-level viremia | 2.23 | 0.005 | 2.00 | 0.003 |
|  | On ART non-suppressed | 4.44 | 0.19 | 4.25 | 0.17 |
|  | Diagnosed but untreated | 4.28 | 0.17 | 4.42 | 0.20 |
|  | Undiagnosed | 4.36 | 0.19 | 4.52 | 0.20 |
| Malawi (2015-16) | On ART low-level viremia | 2.36 | 0.007 | 2.37 | 0.006 |
|  | On ART non-suppressed | 4.16 | 0.16 | 4.15 | 0.16 |
|  | Diagnosed but untreated | 4.21 | 0.17 | 4.54 | 0.20 |
|  | Undiagnosed | 4.05 | 0.15 | 4.43 | 0.20 |
| Rwanda (2018-19) | On ART low-level viremia | 2.24 | 0.006 | 2.24 | 0.004 |
|  | On ART non-suppressed | 4.06 | 0.14 | 4.06 | 0.16 |
|  | Diagnosed but untreated | 4.58 | 0.21 | 4.28 | 0.16 |
|  | Undiagnosed | 4.39 | 0.19 | 4.39 | 0.20 |
| Tanzania (2016-17) | On ART low-level viremia | 2.17 | 0.004 | 2.11 | 0.004 |
|  | On ART non-suppressed | 4.34 | 0.18 | 4.70 | 0.22 |
|  | Diagnosed but untreated | 4.22 | 0.17 | 4.66 | 0.23 |
|  | Undiagnosed | 4.39 | 0.19 | 4.81 | 0.24 |
| Uganda (2016-17) | On ART low-level viremia | 2.14 | 0.004 | 2.16 | 0.004 |
|  | On ART non-suppressed | 4.33 | 0.18 | 4.62 | 0.21 |
|  | Diagnosed but untreated | 4.34 | 0.19 | 4.76 | 0.23 |
|  | Undiagnosed | 4.50 | 0.21 | 4.75 | 0.23 |
| Zambia (2016) | On ART low-level viremia | 2.22 | 0.005 | 2.14 | 0.004 |
|  | On ART non-suppressed | 4.32 | 0.17 | 4.45 | 0.19 |
|  | Diagnosed but untreated | 4.60 | 0.21 | 4.99 | 0.26 |
|  | Undiagnosed | 4.61 | 0.22 | 4.78 | 0.24 |
| Eswatini (2016-17) | On ART low-level viremia | 2.14 | 0.004 | 2.13 | 0.004 |
|  | On ART non-suppressed | 4.37 | 0.18 | 4.38 | 0.19 |
|  | Diagnosed but untreated | 4.30 | 0.18 | 4.57 | 0.22 |
|  | Undiagnosed | 4.28 | 0.18 | 4.55 | 0.21 |
| Lesotho (2016-17) | On ART low-level viremia | 2.17 | 0.005 | 2.18 | 0.004 |
|  | On ART non-suppressed | 4.25 | 0.17 | 4.43 | 0.19 |
|  | Diagnosed but untreated | 4.44 | 0.21 | 4.52 | 0.21 |
|  | Undiagnosed | 4.33 | 0.19 | 4.51 | 0.21 |
| Namibia (2017) | On ART low-level viremia | 2.14 | 0.004 | 2.13 | 0.004 |
|  | On ART non-suppressed | 4.31 | 0.17 | 4.33 | 0.18 |
|  | Diagnosed but untreated | 4.55 | 0.21 | 4.42 | 0.20 |
|  | Undiagnosed | 4.55 | 0.21 | 4.84 | 0.24 |
| Zimbabwe (2015-16) | On ART low-level viremia | 2.16 | 0.004 | 2.16 | 0.003 |
|  | On ART non-suppressed | 4.33 | 0.18 | 4.47 | 0.20 |
|  | Diagnosed but untreated | 4.44 | 0.20 | 4.83 | 0.25 |
|  | Undiagnosed | 4.48 | 0.21 | 4.69 | 0.23 |
