# Supplementary figures and images for: HIV risk behaviour, viraemia, and transmission across HIV cascade stages including low-level viremia: Analysis of 14 cross-sectional population-based HIV Impact Assessment surveys in sub-Saharan Africa
Source: PLOS Glob Public Health. 2024 Apr 4;4(4):e0003030. doi: 10.1371/journal.pgph.0003030 (PMC10994324; doi:10.1371/journal.pgph.0003030)

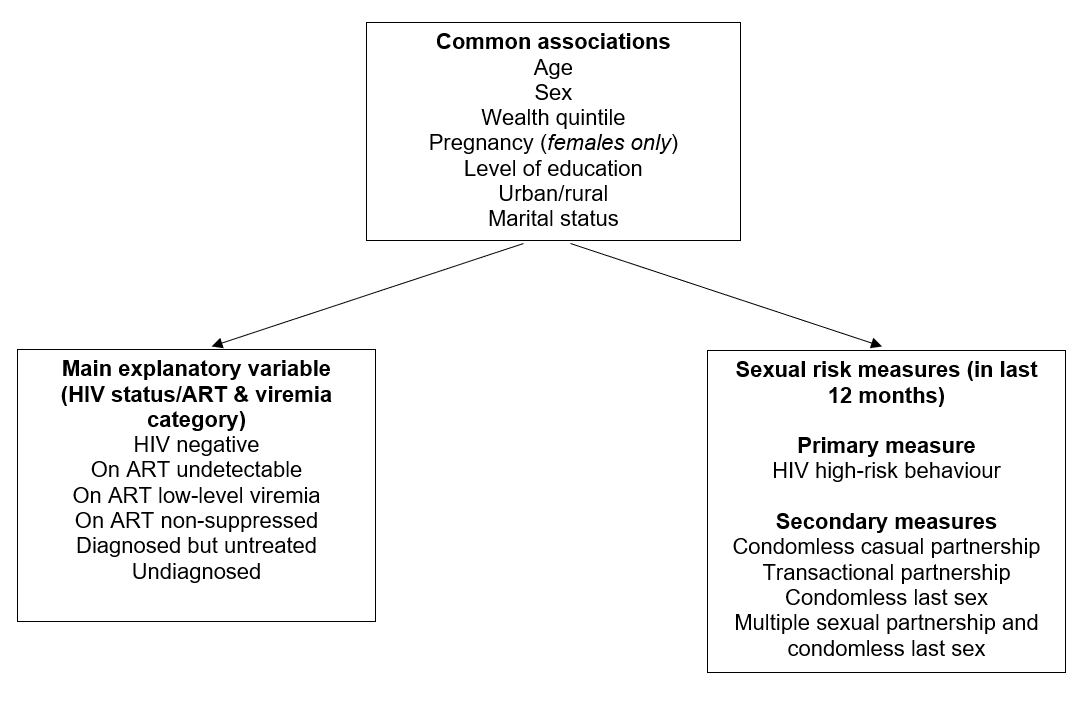


**S1 Fig. Conceptual framework.**

Supplement: S1 Fig — (DOCX) [file pgph.0003030.s013.docx]

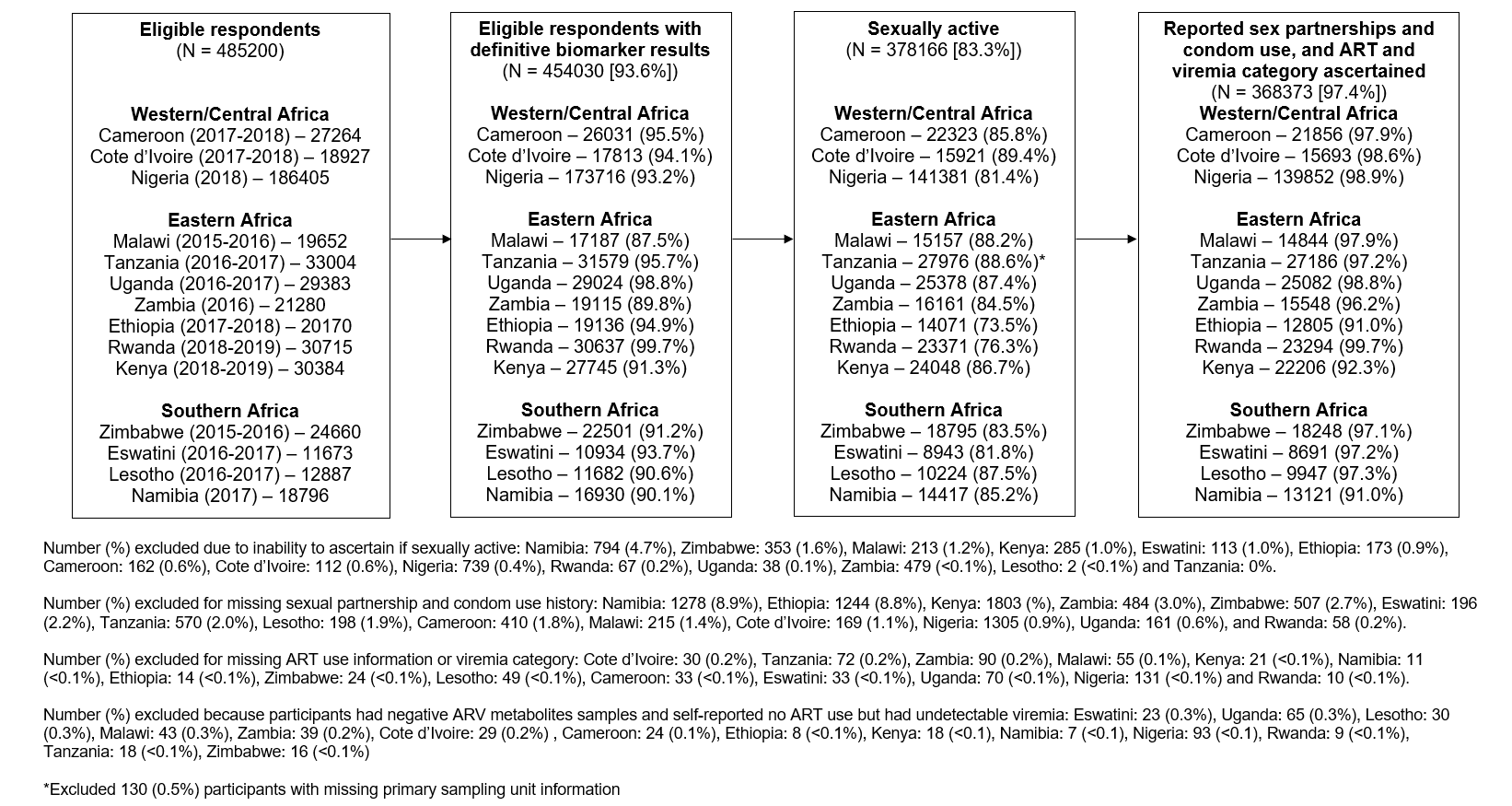


**S2 Fig. Flowchart of participants included in study.**

Supplement: S2 Fig — (DOCX) [file pgph.0003030.s014.docx]
