# Supplementary material for: HIV risk behaviour, viraemia, and transmission across HIV cascade stages including low-level viremia: Analysis of 14 cross-sectional population-based HIV Impact Assessment surveys in sub-Saharan Africa
Source: PLOS Glob Public Health. 2024 Apr 4;4(4):e0003030. doi: 10.1371/journal.pgph.0003030 (PMC10994324; doi:10.1371/journal.pgph.0003030)
Supplement: S3 Fig — (DOCX) [file pgph.0003030.s015.docx]

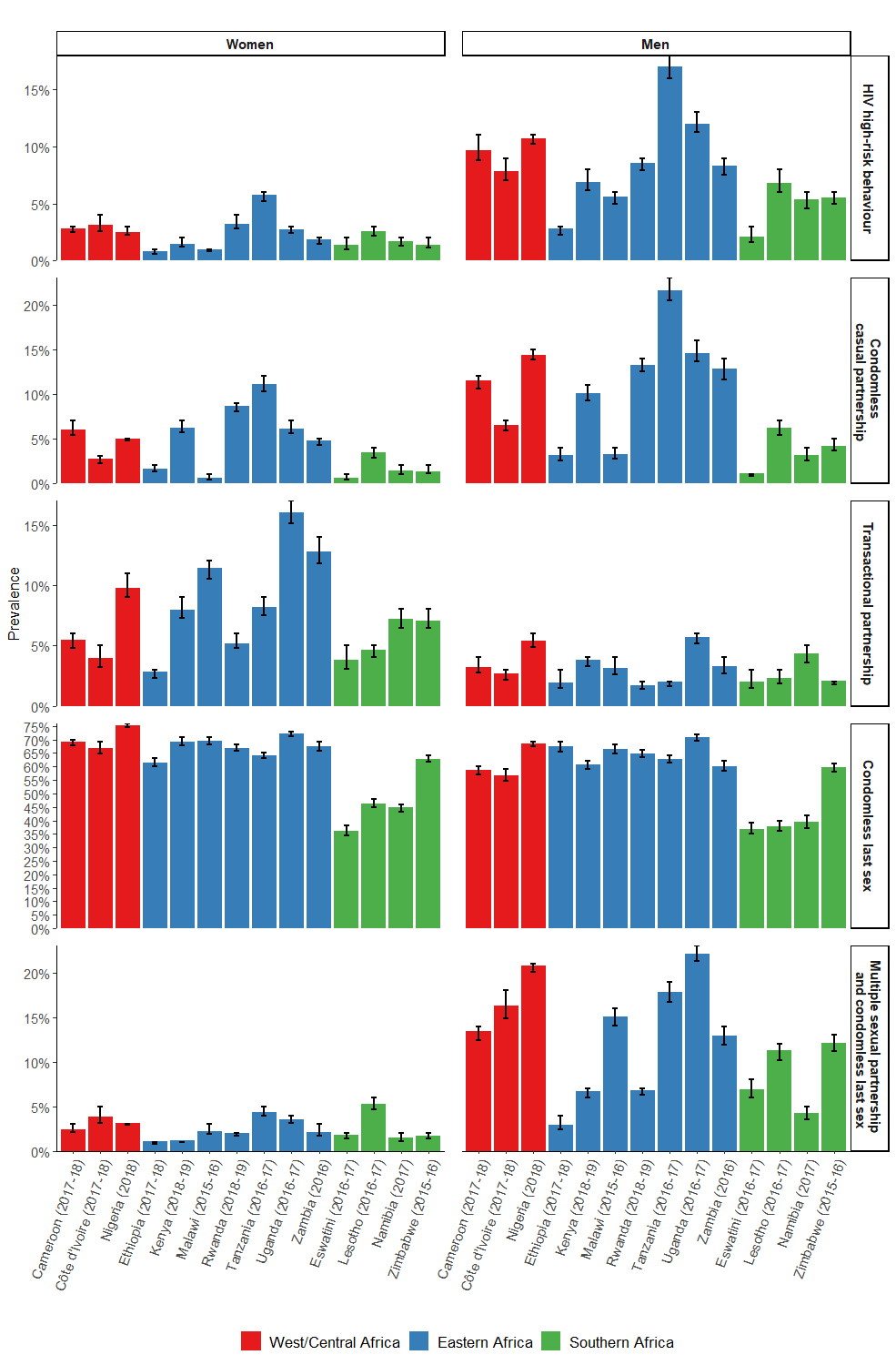


**S3 Fig. Weighted prevalence and 95% confidence intervals of self-reported HIV high-risk behaviour, condomless casual partnerships, transactional partnerships, condomless last sex and both multiple sexual partnership and condomless last sex by sex across 14 PHIA surveys.** HIV high-risk behaviour was defined as self-reporting both ≥2 sexual partners and condomless sex with a non-spousal partner (i.e., ex-spouse, friend, sex-worker, sex-worker client, stranger, or other) in the past 12 months. Condomless casual partnership was defined as self-reporting condomless sex with a non-marital and non-cohabiting partner (i.e., friend, sex worker, sex-worker client, stranger, or other) in the past 12 months, and transactional partnership was defined as self-reporting engaging in a sexual relationship because a partner provided or was expected to provide material or other support (e.g., money, food, or shelter) in the past 12 months. Condomless last sex was defined as self-reporting condomless sex at last sex (with any partner) in the past 12 months and multiple sexual partnership and condomless last sex was reported as self-reporting both ≥2 sexual partners and condomless last sex (with any partner) in the past 12 months.
