# Supplementary material for: HIV risk behaviour, viraemia, and transmission across HIV cascade stages including low-level viremia: Analysis of 14 cross-sectional population-based HIV Impact Assessment surveys in sub-Saharan Africa
Source: PLOS Glob Public Health. 2024 Apr 4;4(4):e0003030. doi: 10.1371/journal.pgph.0003030 (PMC10994324; doi:10.1371/journal.pgph.0003030)
Supplement: S4 Fig — (DOCX) [file pgph.0003030.s016.docx]

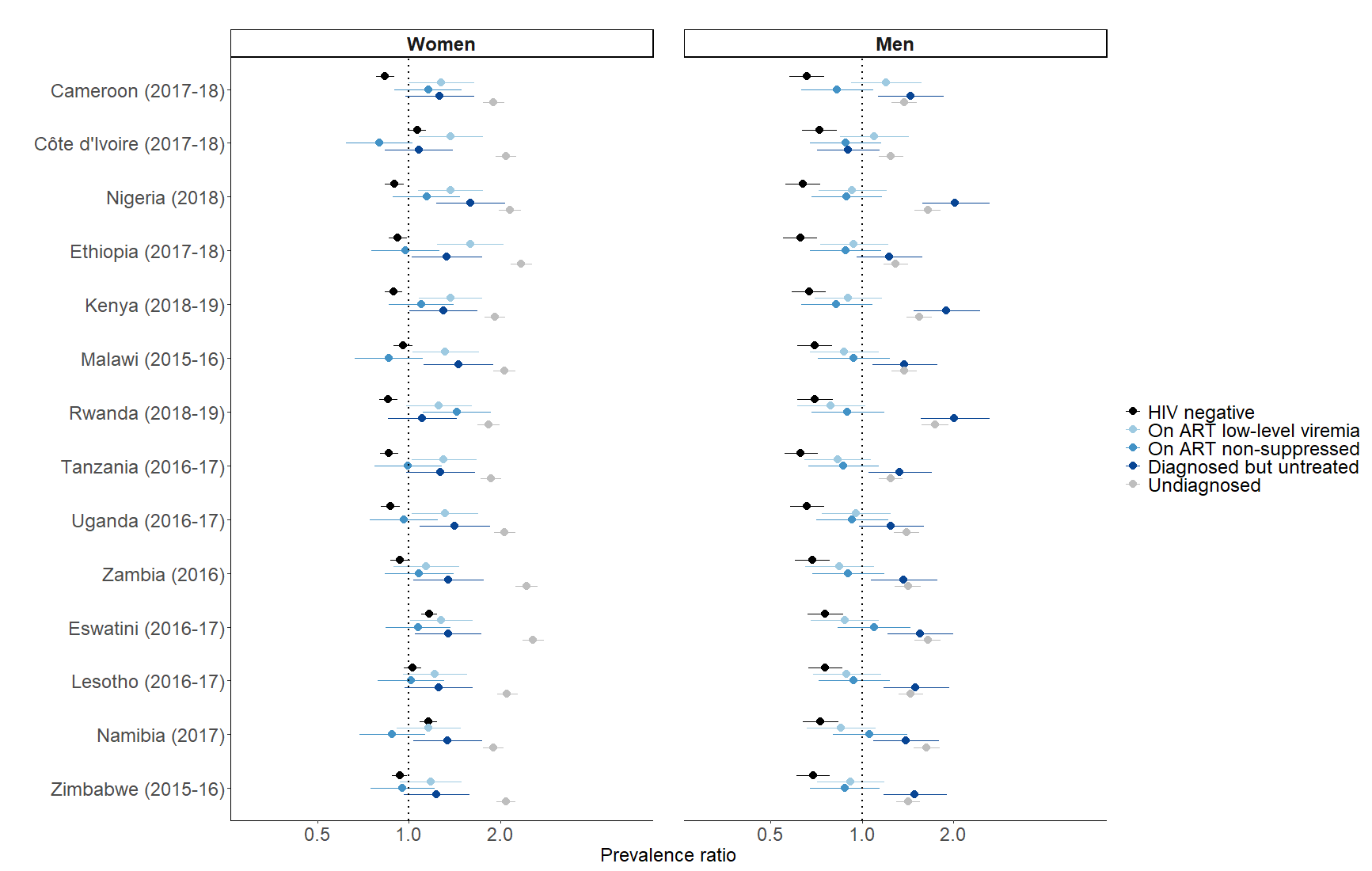


**S4 Fig. Predicted prevalence ratios reporting high-risk sex for each cascade group relative to HIV positive on ART and undetectable (≤50 copies/mL) adults.** Predicted results for each of the 14 survey countries by sex weighted according to the observed distribution of covariates in each group in each survey. The pooled GEE regression model was used to predict the probability of self-reporting HIV high-risk sex given the differential distribution of covariates adjusted for in the pooled model for each country. Line ranges represent 95% confidence intervals.
