# Supplementary material for: HIV risk behaviour, viraemia, and transmission across HIV cascade stages including low-level viremia: Analysis of 14 cross-sectional population-based HIV Impact Assessment surveys in sub-Saharan Africa
Source: PLOS Glob Public Health. 2024 Apr 4;4(4):e0003030. doi: 10.1371/journal.pgph.0003030 (PMC10994324; doi:10.1371/journal.pgph.0003030)
Supplement: S5 Fig — (DOCX) [file pgph.0003030.s017.docx]

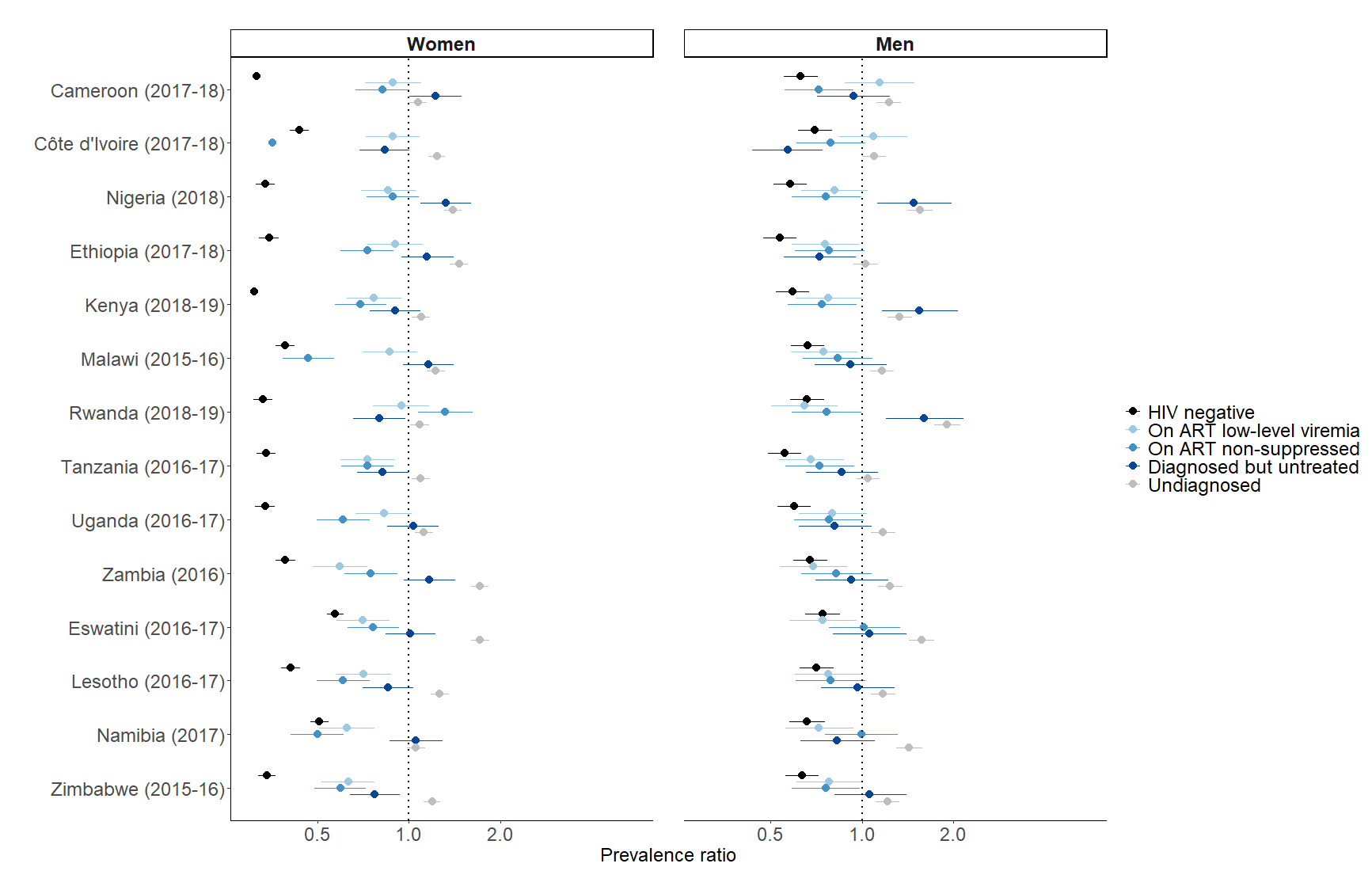


**S5 Fig. Forest plots showing the predicted prevalence ratios and 95% confidence intervals of self-reporting condomless casual partnership for each of the 14 survey countries by sex.** The pooled GEE regression model was used to predict the probability of self-reporting condomless casual partnership given the differential distribution of covariates adjusted for in the pooled model for each country (Note: reference group is “On ART undetectable” group).
