# Supplementary material for: HIV risk behaviour, viraemia, and transmission across HIV cascade stages including low-level viremia: Analysis of 14 cross-sectional population-based HIV Impact Assessment surveys in sub-Saharan Africa
Source: PLOS Glob Public Health. 2024 Apr 4;4(4):e0003030. doi: 10.1371/journal.pgph.0003030 (PMC10994324; doi:10.1371/journal.pgph.0003030)
Supplement: S9 Fig — (DOCX) [file pgph.0003030.s021.docx]

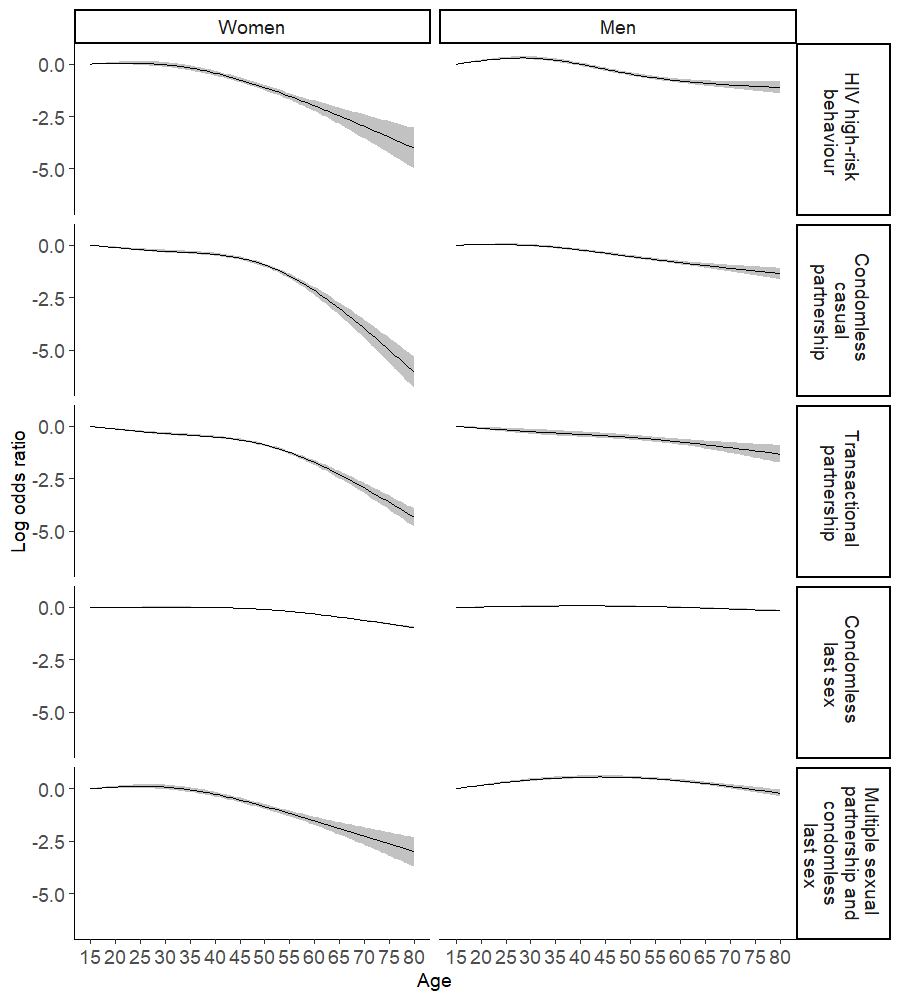


**S9 Fig. Estimated log-odds ratio and 95% confidence intervals of HIV high-risk behaviour, condomless casual partnership, transactional partnership, condomless last sex and self-reporting both multiple sexual partnership and condomless last sex by age predicted by the spline effects by sex and risk behaviour.**
