# Supplementary material for: HIV risk behaviour, viraemia, and transmission across HIV cascade stages including low-level viremia: Analysis of 14 cross-sectional population-based HIV Impact Assessment surveys in sub-Saharan Africa
Source: PLOS Glob Public Health. 2024 Apr 4;4(4):e0003030. doi: 10.1371/journal.pgph.0003030 (PMC10994324; doi:10.1371/journal.pgph.0003030)
Supplement: S10 Fig — (A) Boxplots of the log10 viral load distribution and (B) Mean annual transmission rates and 95% confidence intervals for each PLHIV population sub-group estimated using the Hill function by sex. (DOCX) [file pgph.0003030.s022.docx]

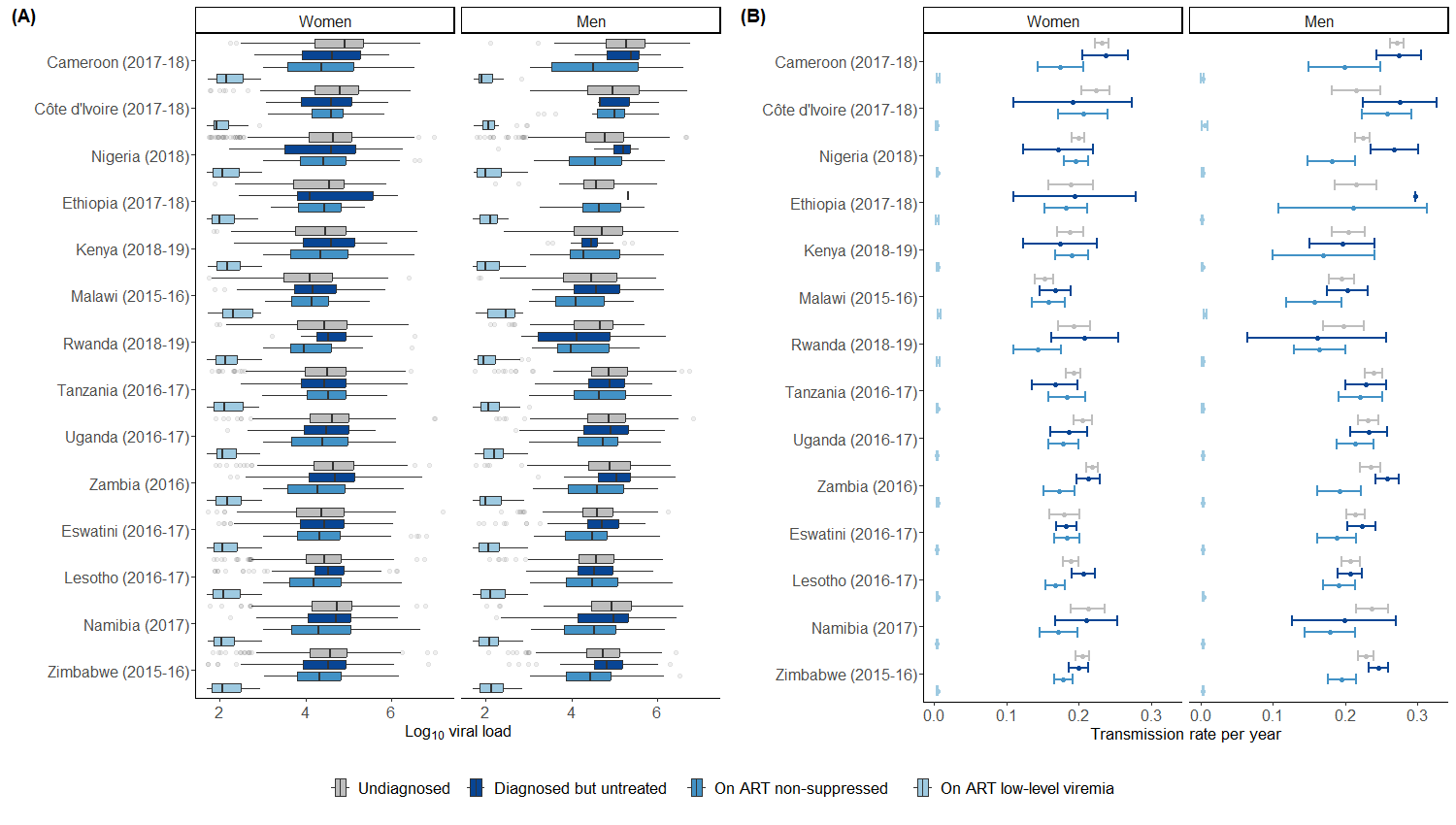


**S10 Fig. (A) Boxplots of the log_10_ viral load distribution and (B) Mean annual transmission rates (estimated using the Hill function from Fraser et al.) and 95% confidence intervals for each PLHIV population sub-group estimated using the Hill function by sex.** Estimates are weighted to account for survey sampling weights. Note: unable to reliably estimate distribution and confidence interval due to small number of men diagnosed but untreated in the Ethiopia PHIA survey.
