# Supplementary material for: HIV risk behaviour, viraemia, and transmission across HIV cascade stages including low-level viremia: Analysis of 14 cross-sectional population-based HIV Impact Assessment surveys in sub-Saharan Africa
Source: PLOS Glob Public Health. 2024 Apr 4;4(4):e0003030. doi: 10.1371/journal.pgph.0003030 (PMC10994324; doi:10.1371/journal.pgph.0003030)
Supplement: S11 Fig — (A) Plot showing the transmission rate as a function of viral load using the Hill function. (B) Distribution of viral load for each PLHIV subgroup using data from Lesotho 2016–2017 survey. (DOCX) [file pgph.0003030.s023.docx]

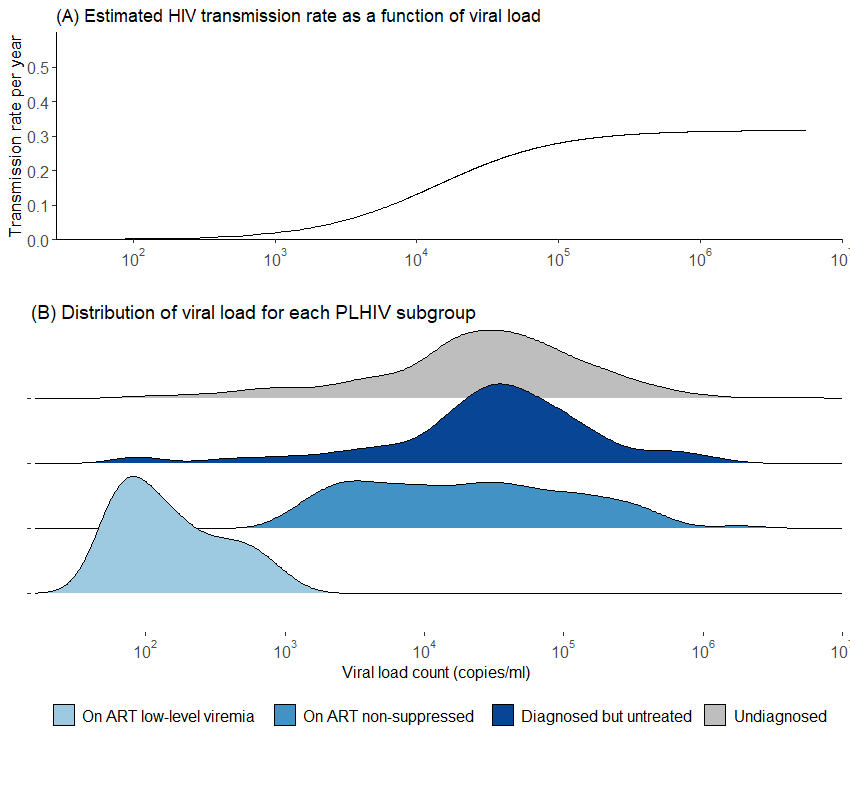


**S11 Fig. (A) Plot showing the transmission rate as a function of viral load using the Hill function. (B) Distribution of viral load for each PLHIV subgroup using data from Lesotho 2016-2017 survey.** Hill function is described as $\beta\left( V \right)= \frac{\beta_{max} \times V^{\beta_{k}}}{V^{\beta_{k}}+ {(\beta_{50})}^{\beta_{k}}}$ ; where $\beta_{max}$is the maximum infection rate per annum, $\beta_{50}$ is the viral load at which infectiousness is half its maximum, and $\beta_{k}$ is the steepness of the increase in infectiousness as a function of viral load. Parameter values for the Hill function are $\beta_{max}$= 0.317 per year, $\beta_{50}$= 13,938 HIV RNA copies/mL and $\beta_{k}$ = 1.02. (Fraser et al., 2007)
