# Supplementary material for: HIV risk behaviour, viraemia, and transmission across HIV cascade stages including low-level viremia: Analysis of 14 cross-sectional population-based HIV Impact Assessment surveys in sub-Saharan Africa
Source: PLOS Glob Public Health. 2024 Apr 4;4(4):e0003030. doi: 10.1371/journal.pgph.0003030 (PMC10994324; doi:10.1371/journal.pgph.0003030)
Supplement: S12 Fig — (DOCX) [file pgph.0003030.s024.docx]

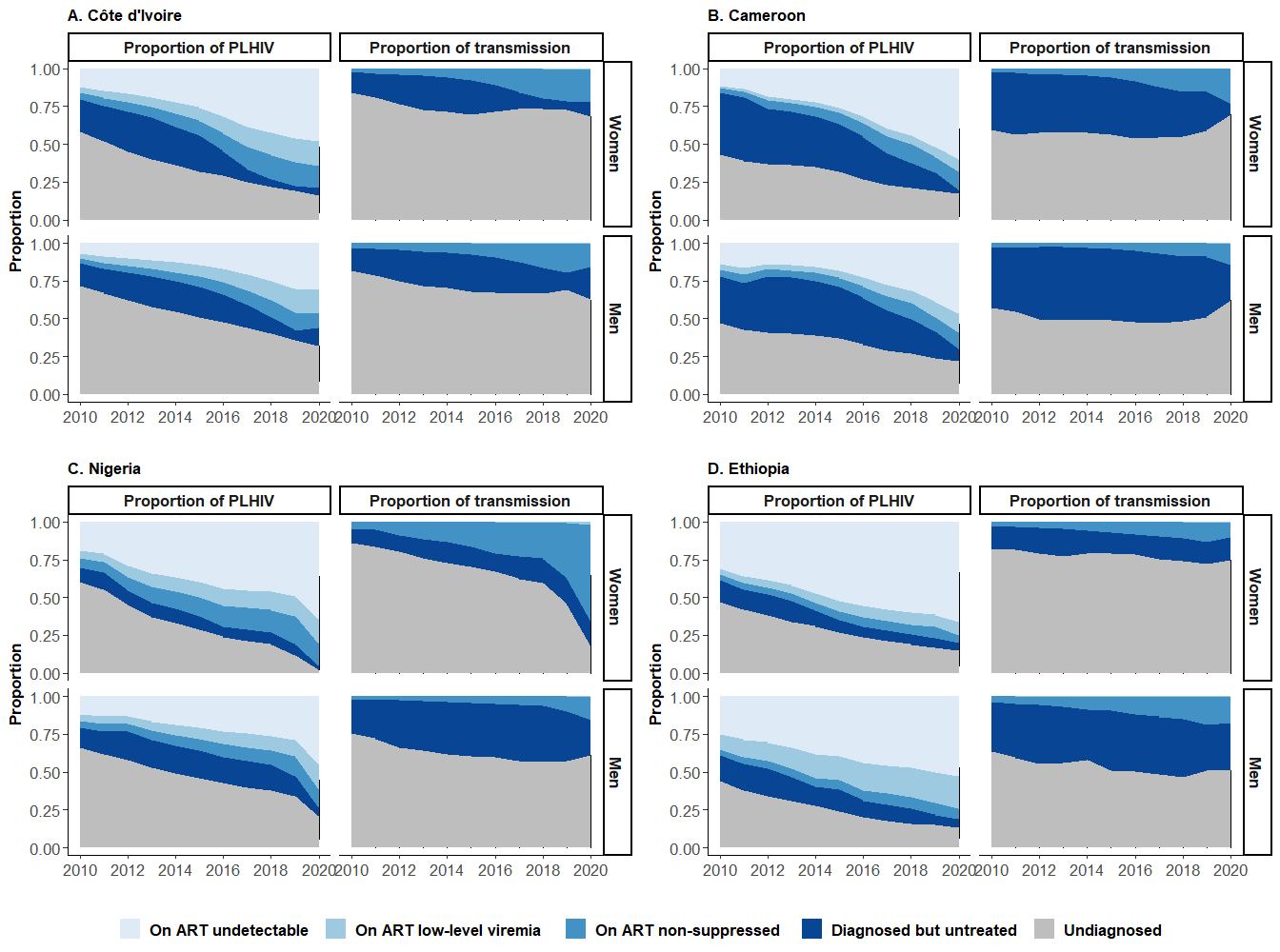


**S12 Fig. Estimated proportion of PLHIV in each sub-group and proportion of transmission attributed to each PLHIV sub-group from 2010-2020 in (A) Côte d’Ivoire (B) Cameroon (C) Nigeria and (D) Ethiopia.**


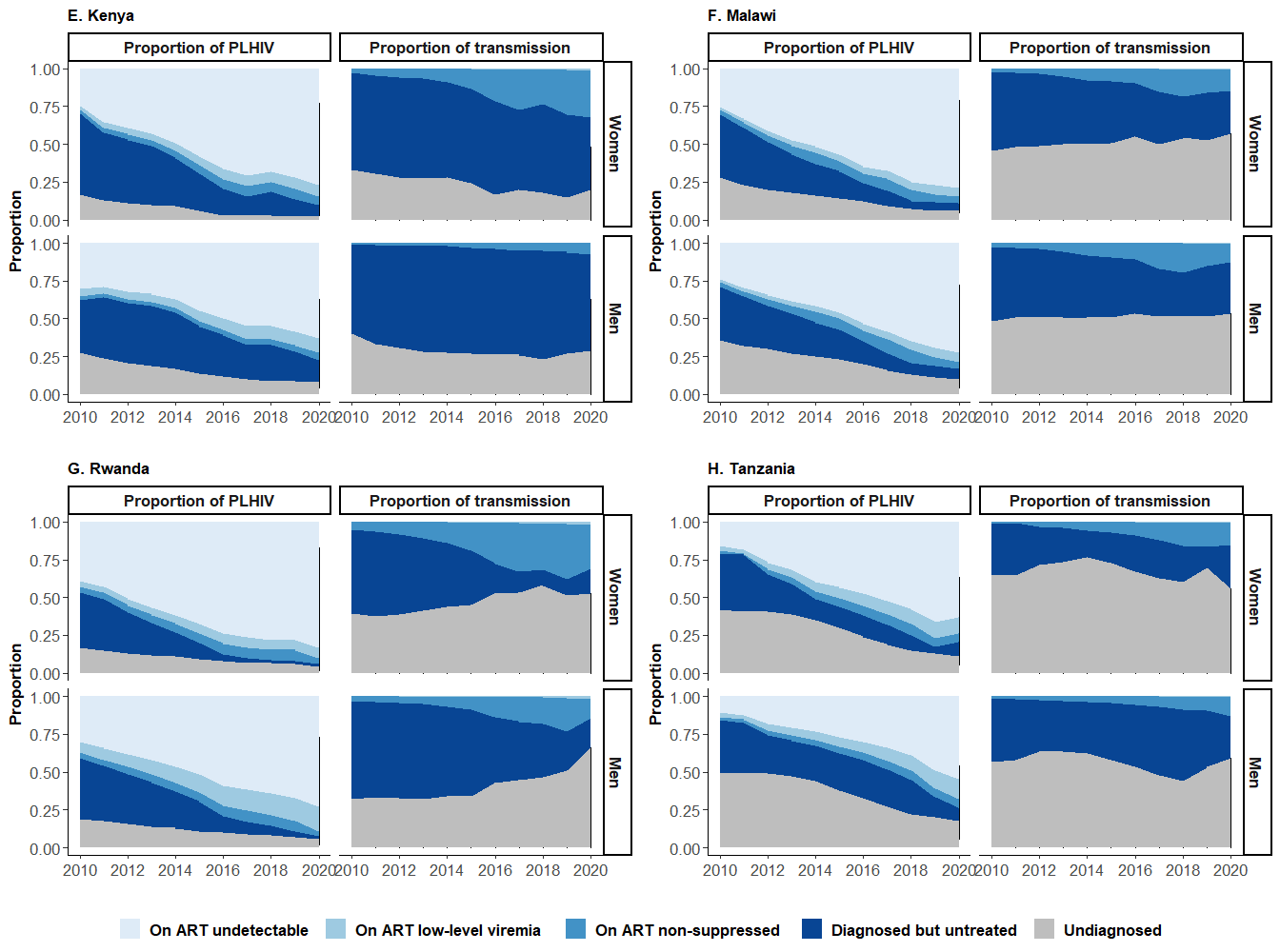


**S12 Fig continued. Estimated proportion of PLHIV in each sub-group and proportion of transmission attributed to each PLHIV sub-group from 2010-2020 in (E) Kenya (F) Malawi (G) Rwanda and (H) Tanzania.**


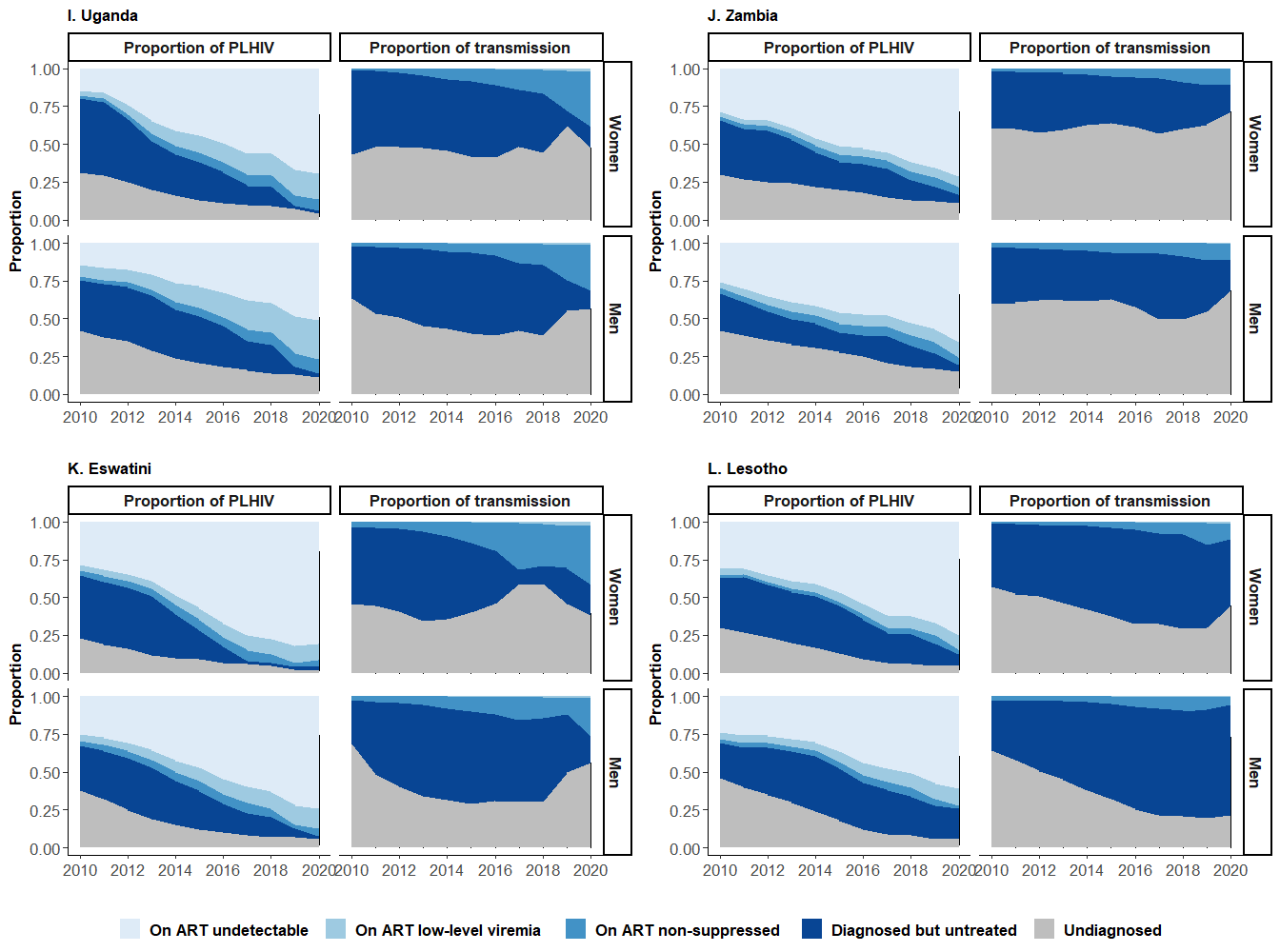


**S12 Fig continued. Estimated proportion of PLHIV in each sub-group and proportion of transmission attributed to each PLHIV sub-group from 2010-2020 in (I) Uganda (J) Zambia (K) Eswatini and (L) Lesotho.**


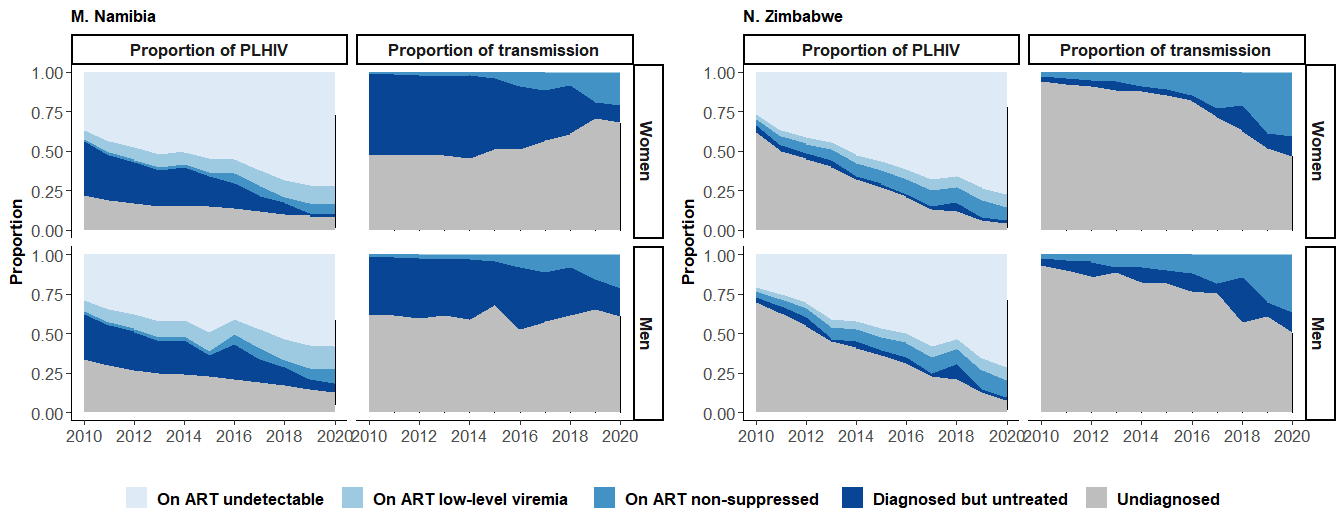


**S12 Fig continued. Estimated proportion of PLHIV in each sub-group and proportion of transmission attributed to each PLHIV sub-group from 2010-2020 in (M) Namibia and (N) Zimbabwe.**
