# Supplementary material for: HIV risk behaviour, viraemia, and transmission across HIV cascade stages including low-level viremia: Analysis of 14 cross-sectional population-based HIV Impact Assessment surveys in sub-Saharan Africa
Source: PLOS Glob Public Health. 2024 Apr 4;4(4):e0003030. doi: 10.1371/journal.pgph.0003030 (PMC10994324; doi:10.1371/journal.pgph.0003030)
Supplement: S14 Fig — (DOCX) [file pgph.0003030.s026.docx]

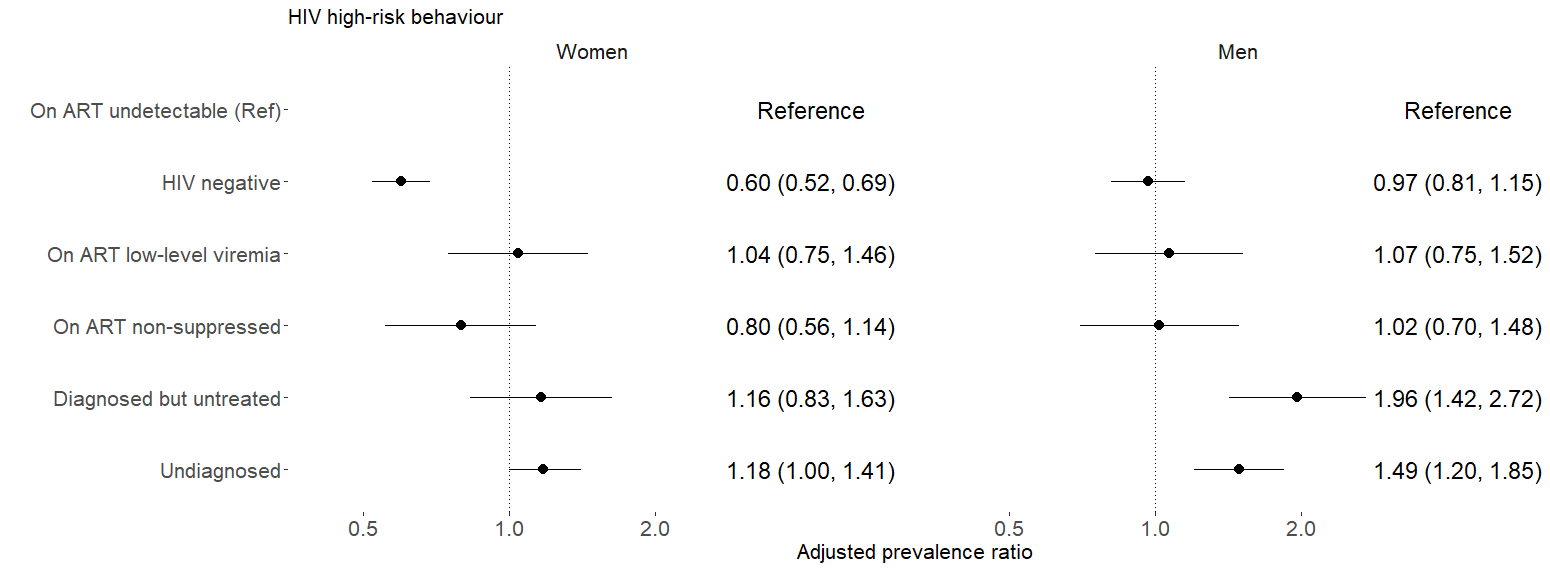


**S14 Fig.** **Forest plots showing the adjusted prevalence ratios of self-reporting high HIV high-risk behaviour by sub-groups (including normalized survey weight in regression mode).** Results are stratified by sex and models were adjusted for age, level of education, wealth quintile, marital status, urban/rural dwelling or urbanicity size, and pregnancy status in women.
