# Supplementary material for: HIV risk behaviour, viraemia, and transmission across HIV cascade stages including low-level viremia: Analysis of 14 cross-sectional population-based HIV Impact Assessment surveys in sub-Saharan Africa
Source: PLOS Glob Public Health. 2024 Apr 4;4(4):e0003030. doi: 10.1371/journal.pgph.0003030 (PMC10994324; doi:10.1371/journal.pgph.0003030)
Supplement: S15 Fig — (DOCX) [file pgph.0003030.s027.docx]

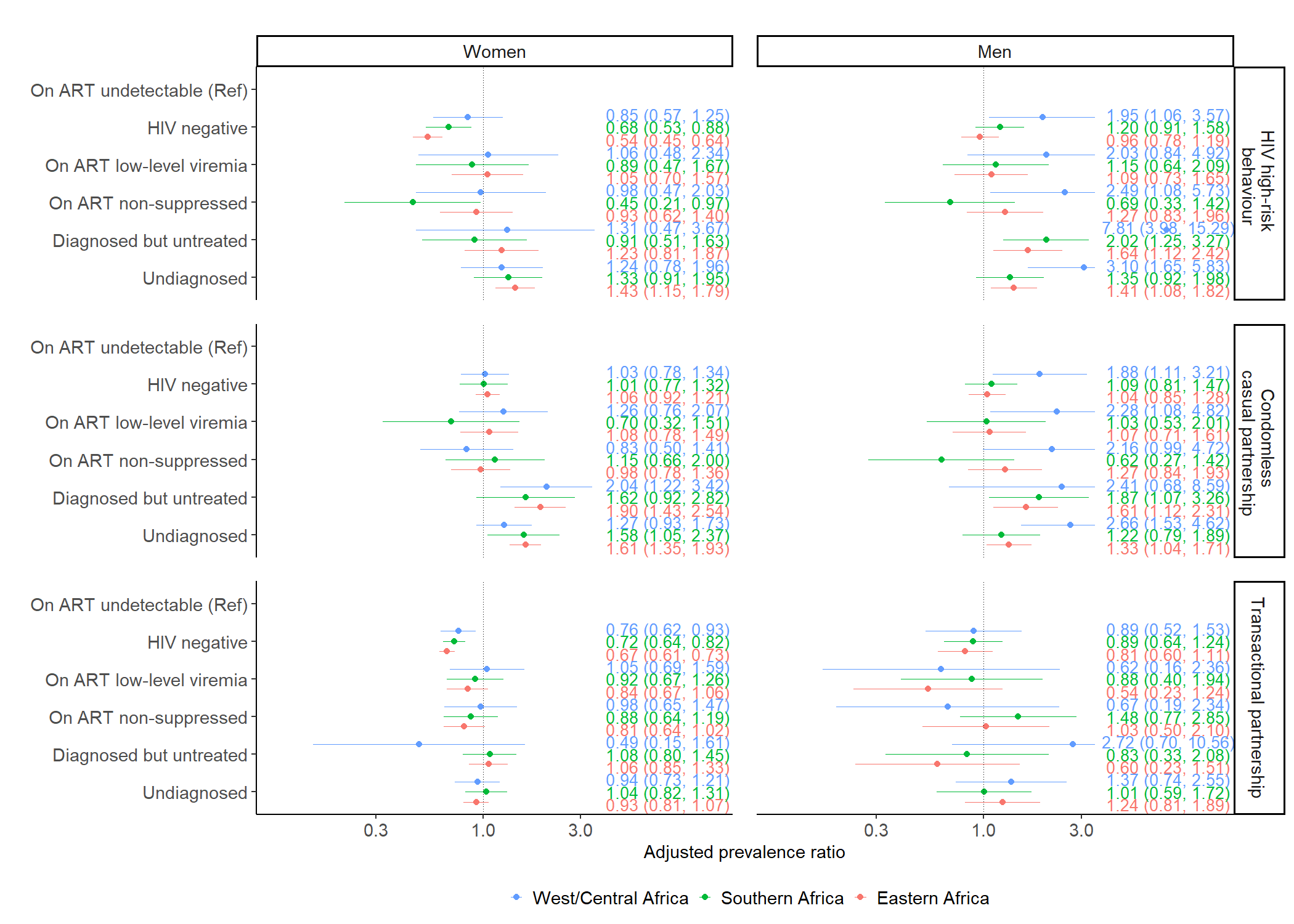


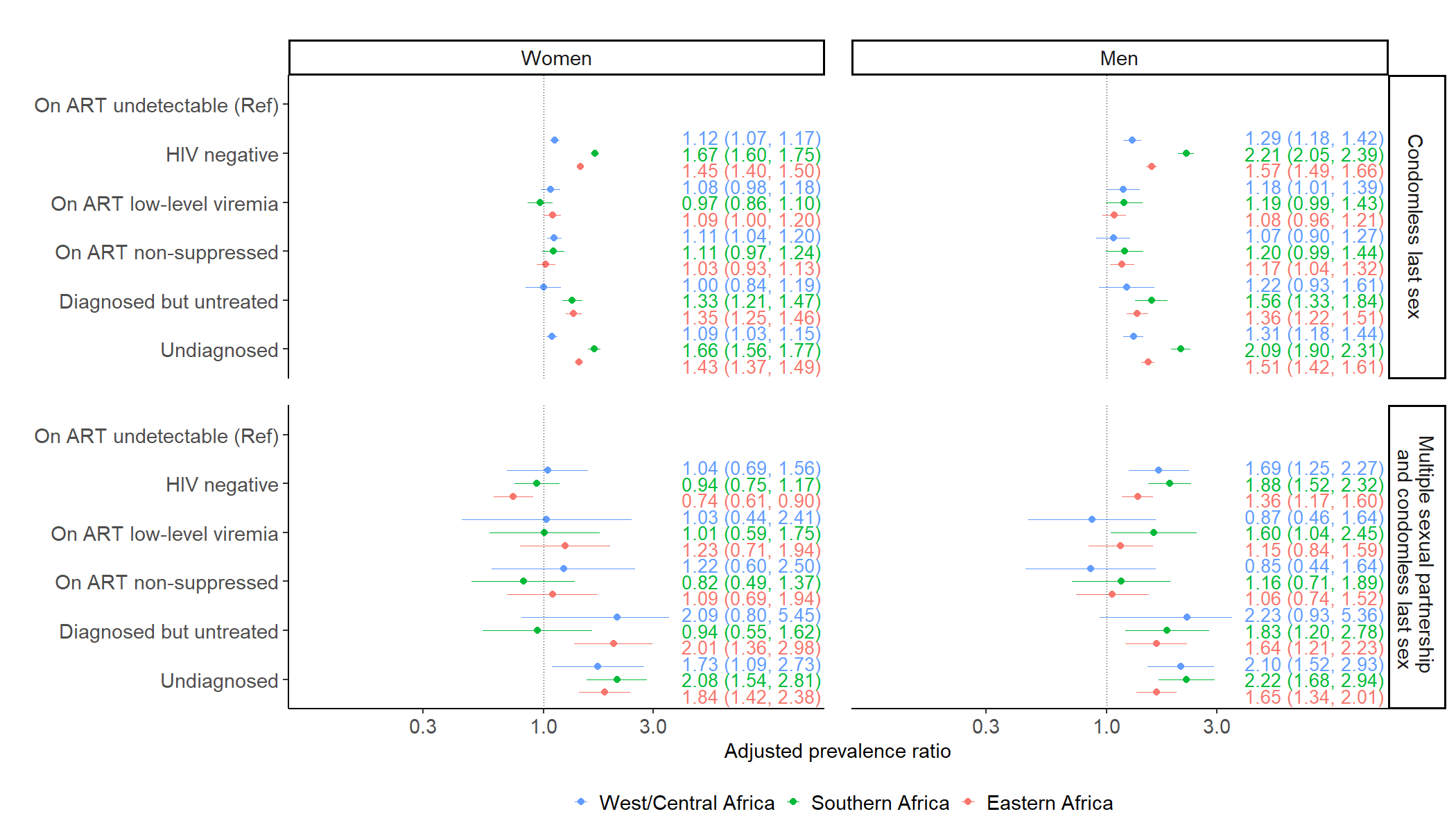


**S15 Fig. Forest plots showing the region-specific adjusted prevalence ratios of self-reporting HIV high-risk behaviour, condomless casual partnerships, transactional partnerships, condomless last sex and self-reporting both multiple sexual partnership and condomless last sex for each subgroup.** Results are stratified by region and sex and models were adjusted for age, level of education, wealth quintile, marital status, urban/rural dwelling or urbanicity size, and pregnancy status in women. Note: x-axis is log_10_ scaled and prevalence ratios are truncated at 3.5 in figure.
