# Supplementary material for: HIV risk behaviour, viraemia, and transmission across HIV cascade stages including low-level viremia: Analysis of 14 cross-sectional population-based HIV Impact Assessment surveys in sub-Saharan Africa
Source: PLOS Glob Public Health. 2024 Apr 4;4(4):e0003030. doi: 10.1371/journal.pgph.0003030 (PMC10994324; doi:10.1371/journal.pgph.0003030)
Supplement: S17 Fig — (DOCX) [file pgph.0003030.s029.docx]

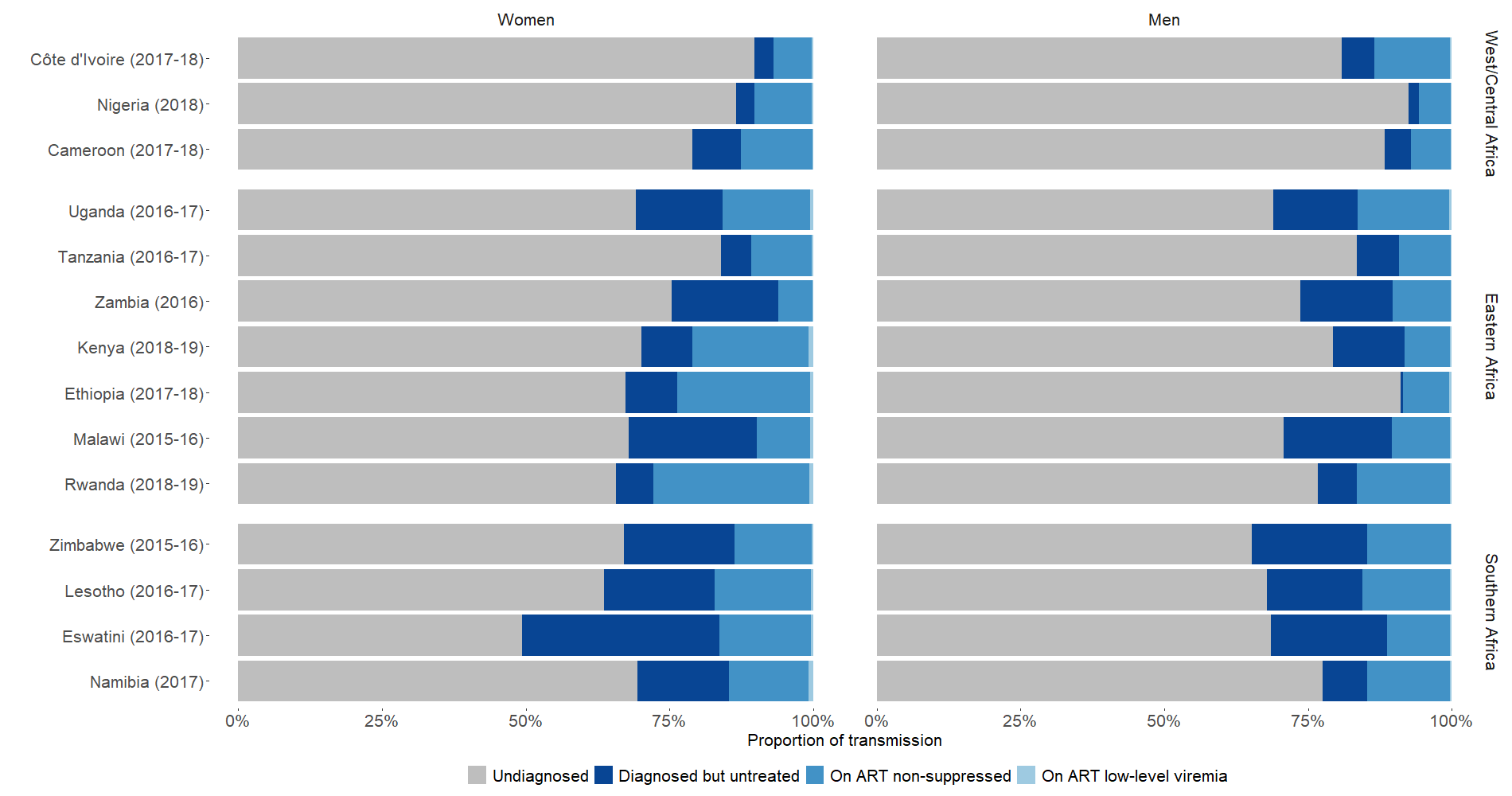


**S17 Fig. Proportion of transmission attributed to PLHIV sub-group by sex across all 14 PHIA surveys** (sensitivity analysis using Hill function of transmission and viremia relationship, and adjusted prevalence ratio of self-reporting condomless casual partnerships in transmission equation).
