# Supplementary material for: HIV risk behaviour, viraemia, and transmission across HIV cascade stages including low-level viremia: Analysis of 14 cross-sectional population-based HIV Impact Assessment surveys in sub-Saharan Africa
Source: PLOS Glob Public Health. 2024 Apr 4;4(4):e0003030. doi: 10.1371/journal.pgph.0003030 (PMC10994324; doi:10.1371/journal.pgph.0003030)
Supplement: S23 Fig — Plot showing the transmission rate as a function of viral load using the linear function for (A) Women-to-Men and (B) Men-to-Women, and distribution of viral load for each PLHIV subgroup using data from Lesotho 2016–2017 survey for (C) women and (D) men. (DOCX) [file pgph.0003030.s035.docx]

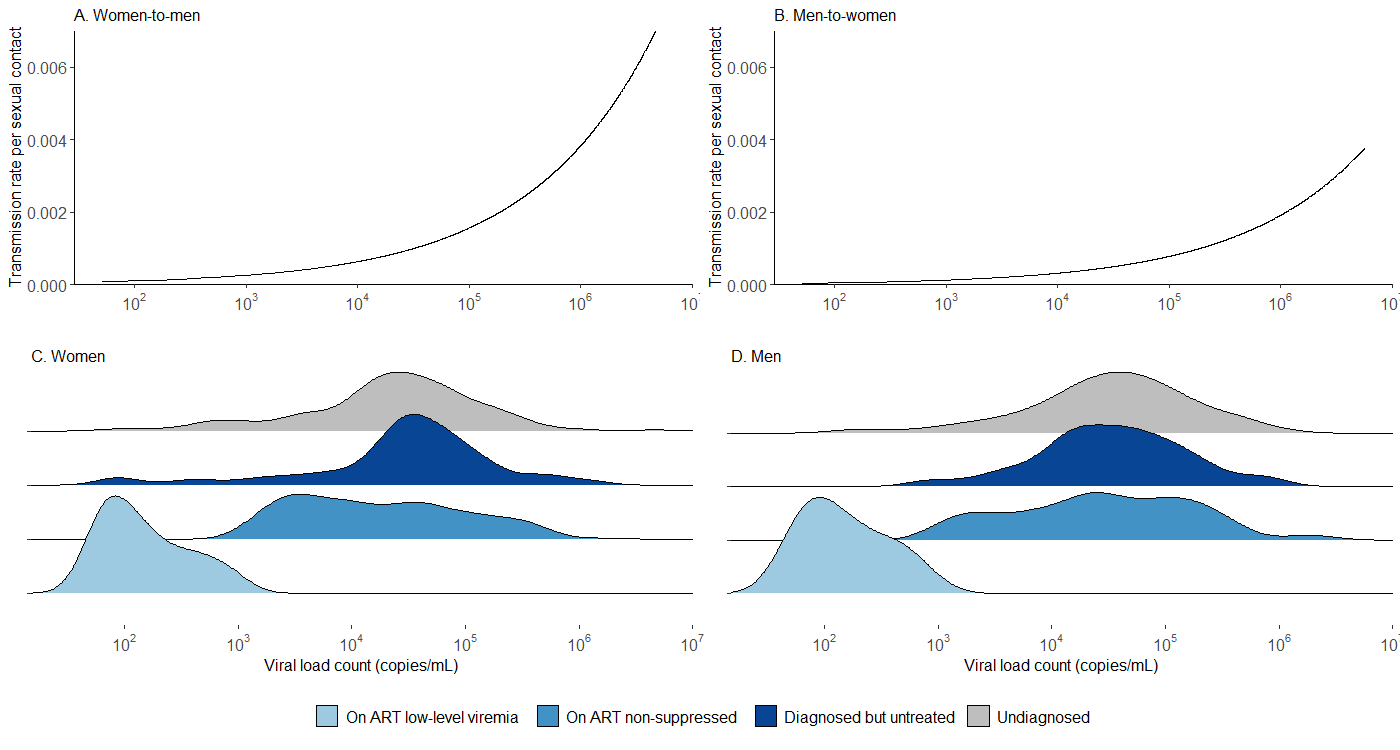


**S23 Fig. Plot showing the transmission rate as a function of viral load using the linear function for (A) Women-to-Men and (B) Men-to-Women, and distribution of viral load for each PLHIV subgroup using data from Lesotho 2016-2017 survey for (C) women and (D) men.** Linear function: $\beta_{1}= {2.45}^{\log_{10} (V_{1}/V_{0})}\beta_{0}$, where $\beta_{0}$ is the probability of HIV transmission from a person with a baseline viral load $V_{0}$ and $\beta_{1}$is the transmission probability corresponding to any other viral load V₁. Parameter values ($\beta_{0}$= 0.0005 for Men-to-Women and 0.001 for Women-to-Men transmission and $V_{0}$ = 10^4.5^ copies per mL from Wilson et al., 2008.
