# Supplementary material for: HIV risk behaviour, viraemia, and transmission across HIV cascade stages including low-level viremia: Analysis of 14 cross-sectional population-based HIV Impact Assessment surveys in sub-Saharan Africa
Source: PLOS Glob Public Health. 2024 Apr 4;4(4):e0003030. doi: 10.1371/journal.pgph.0003030 (PMC10994324; doi:10.1371/journal.pgph.0003030)
Supplement: S24 Fig — (DOCX) [file pgph.0003030.s036.docx]

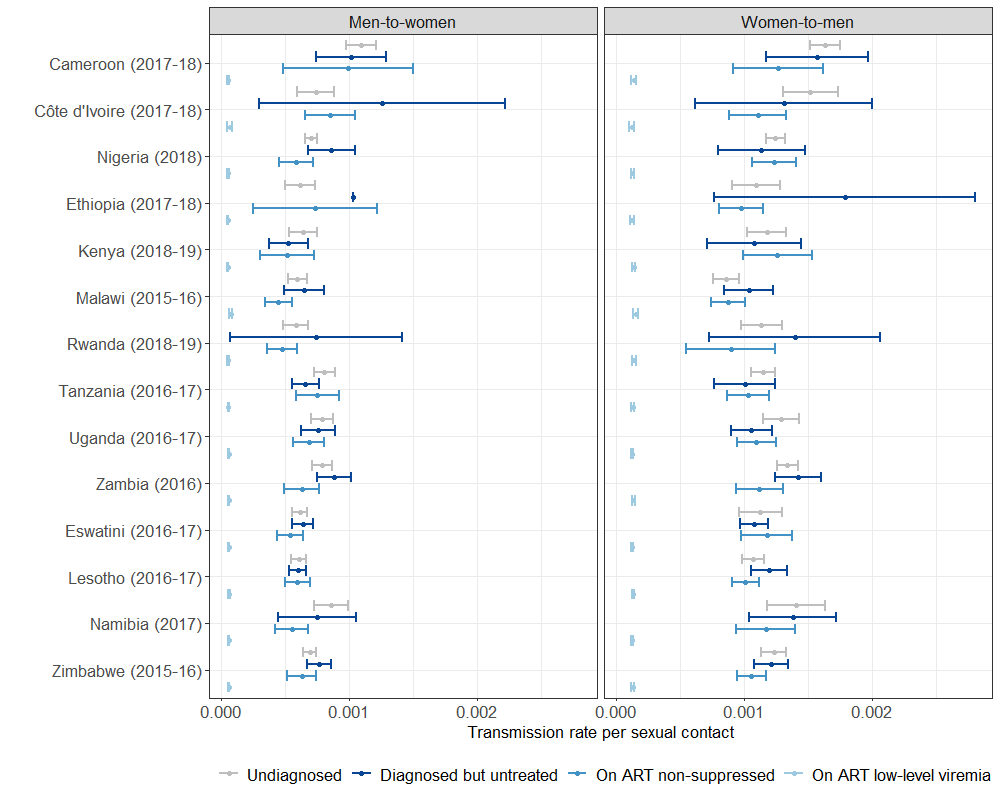


**S24 Fig. Sex-stratified weighted average transmission rates per sexual contact and 95% confidence intervals for each PLHIV population sub-group estimated using the linear function for each of the 14 PHIA surveys.** Note: the linear function assumes different baseline probability of transmission for women-to-men and men-to-women transmission (Wilson et al.).
